# Evaluating the accuracy, reliability, and readability of AI chatbots in delivering postpartum depression information

**DOI:** 10.3389/fpsyt.2026.1881536

**Published:** 2026-08-12

**Authors:** Man Yang, Hui Liu, Shuyan Lin, Linyan Liu, Yunxia Wang, Xiuling Qiu, Ling Wei, Li Zhou, Xinxin Wang

**Affiliations:** Department of Obstetrics, Shenzhen Nanshan Maternity and Child Healthcare Hospital, Shenzhen, China

**Keywords:** artificial intelligence, health information, large language models, patient education, postpartum depression

## Abstract

**Background:**

Postpartum depression (PPD) is a common perinatal psychiatric disorder with significant implications for maternal and infant health. Artificial intelligence (AI) chatbots have emerged as widely accessible tools for health information, but their performance in providing accurate, reliable, and readable PPD related information remains underexplored.

**Methods:**

We evaluated six AI chatbots, ChatGPT-5, ChatGPT-4o, Claude Sonnet 4.5, DeepSeek-V3.2, DeepSeek-R1, and Gemini 2.5 Pro, using 200 standardized multiple choice questions (MCQs) on PPD to assess validity. ChatGPT-4o and DeepSeek-R1 were included only in the MCQ based validity analysis as earlier version comparators. Reliability and readability were further assessed using 20 core public education questions in the four latest models: ChatGPT-5, Claude Sonnet 4.5, DeepSeek-V3.2, and Gemini 2.5 Pro. Chatbot performance was assessed across three dimensions: validity, reliability, and readability. Each MCQ was presented three times independently, and each core public education question was assessed once per model.

**Results:**

In the six model MCQ based validity analysis, ChatGPT-5 achieved the highest overall accuracy on MCQs (97.50% ± 0.50%). In the four models reliability and readability analyses, ChatGPT-5 obtained the highest DISCERN, EQIP, and GQS scores, suggesting relatively better content quality and user oriented usefulness. However, JAMA benchmark scores were low across all models, including ChatGPT-5, indicating limited transparency, source attribution, currency, and disclosure. All models produced outputs exceeding the recommended sixth grade reading level, although ChatGPT-5 and Gemini 2.5 Pro were relatively more accessible.

**Conclusion:**

AI chatbots, particularly ChatGPT-5, showed potential as supplementary tools for providing postpartum depression related information, especially in standardized MCQ based assessment. However, this study did not evaluate clinical safety, patient comprehension, user behavior, or real world effectiveness, and suboptimal readability may limit accessibility for users with lower health or digital literacy. Inadequate transparency, limited source attribution, and suboptimal readability indicate that AI chatbots should not be used as autonomous sources of postpartum mental health guidance and should not replace professional assessment or care.

## 1 Introduction

Postpartum depression (PPD) is one of the most common perinatal psychiatric disorders, affecting approximately 10–15% of women worldwide, with an average prevalence of 14.7% in China (1). Recurrence in subsequent pregnancies ranges from 20–30% (2). PPD arises from a combination of hormonal fluctuations, genetic predisposition, psychosocial stressors, and insufficient social support (3, 4). Despite its prevalence, PPD is often underrecognized and undertreated, particularly in younger mothers, high risk pregnancies, or women lacking family support (5, 6). Untreated PPD can result in sleep disturbances, emotional instability, impaired mother-infant bonding, increased risk of self-harm, and delayed cognitive and emotional development in infants, imposing substantial burdens on families and society (7–9).

Artificial intelligence (AI) chatbots have recently emerged as accessible tools for health information. Patients increasingly rely on AI conversational agents such as ChatGPT and Claude for medical and psychological advice (10). While these systems can generate coherent and informative responses, the accuracy, reliability, and readability of AI generated content in the context of PPD remain largely unexamined (11).

Prior research on AI in healthcare has primarily focused on general medical knowledge or other patient education topics, often using limited metrics for evaluation (12). Given the growing use of AI for health guidance, systematically assessing whether AI chatbots provide evidence-based, reliable, and comprehensible information is essential. Such evaluation is critical for safe and effective integration of AI into public health education, particularly for vulnerable populations such as postpartum women.

In this study, we evaluated AI chatbots in two analysis modules. First, six AI chatbots, ChatGPT-5, ChatGPT-4o, Claude Sonnet 4.5, DeepSeek-V3.2, DeepSeek-R1, and Gemini 2.5 Pro, were assessed for MCQ based validity in answering standardized PPD questions. ChatGPT-4o and DeepSeek-R1 were included only in this validity analysis as earlier version comparators. Second, four latest models, ChatGPT-5, Claude Sonnet 4.5, DeepSeek-V3.2, and Gemini 2.5 Pro, were evaluated for reliability and readability using public education questions on PPD. This systematic analysis provides insight into the potential and limitations of AI driven patient education for postpartum mental health.

## 2 Methods

### 2.1 Study design

**Figure 1:**
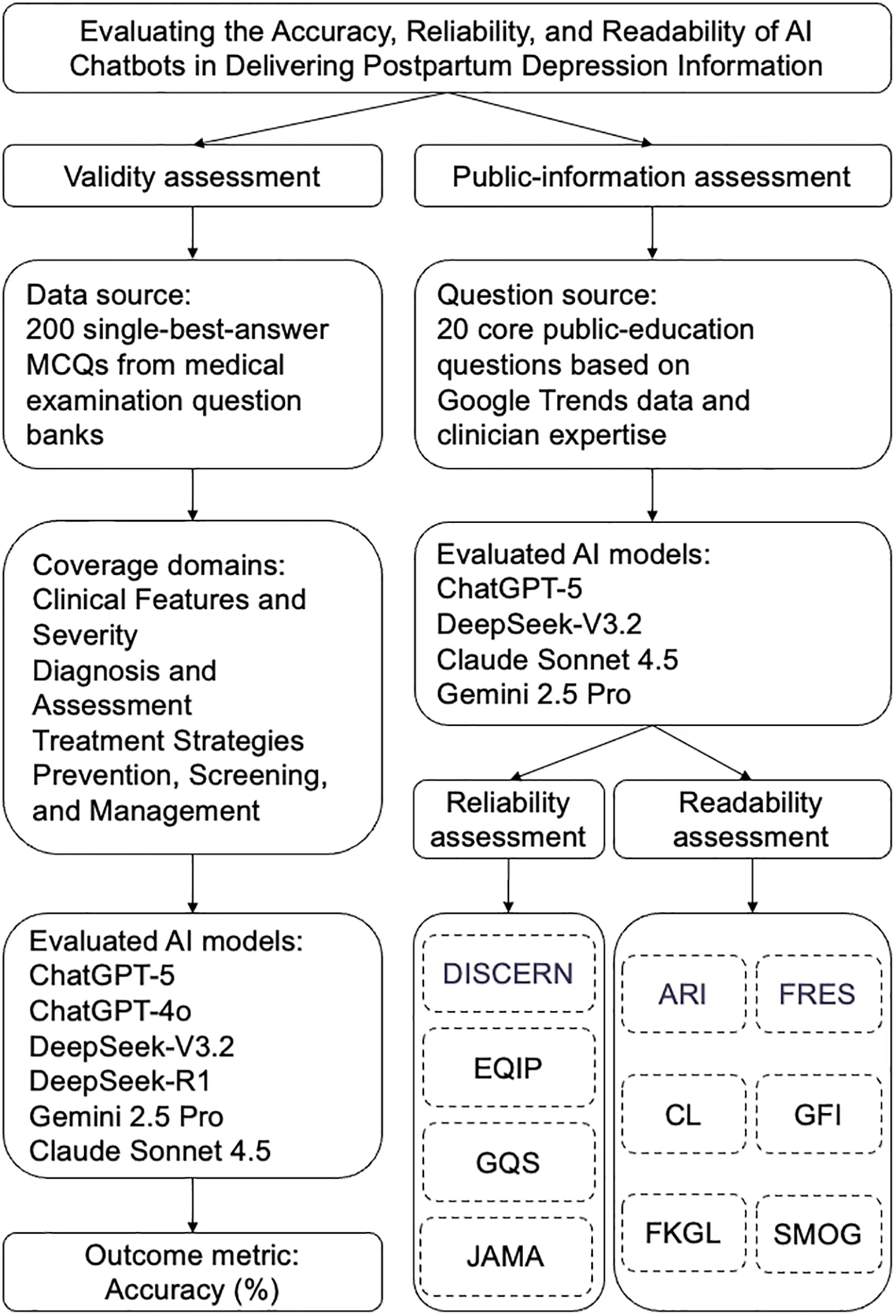
ChatGPT-4o and DeepSeek-R1 were included only in the MCQ based validity analysis as earlier version comparators. Reliability and readability analyses were conducted only for the four latest models: ChatGPT-5, Claude Sonnet 4.5, DeepSeek-V3.2, and Gemini 2.5 Pro.

This study was designed to evaluate the performance of AI chatbots in responding to standardized questions on postpartum depression. The evaluation included two analysis modules. In the MCQ based validity analysis, six AI chatbots were evaluated: ChatGPT-5, ChatGPT-4o, Claude Sonnet 4.5, DeepSeek-V3.2, DeepSeek-R1, and Gemini 2.5 Pro. ChatGPT-4o and DeepSeek-R1 were included only in this validity analysis as earlier version comparators to explore model iteration effects. In the reliability and readability analyses, four latest models, ChatGPT-5, Claude Sonnet 4.5, DeepSeek-V3.2, and Gemini 2.5 Pro, were evaluated using 20 core public education questions. The selected models were included because of their broad applicability, public accessibility, and capacity to provide responses to medical and health related queries. The study workflow is shown schematically in **Figure 1**. 

To improve methodological transparency and reproducibility, we documented the access route, platform or provider, access date, language, account or subscription status, generation settings, and conversation-history handling for each evaluated AI model (**Supplementary Table 1**). Different access routes were used for different analyses. For the MCQ based validity analysis, responses were obtained through the official APIs of the corresponding model providers. The six models included in this analysis were ChatGPT-5, ChatGPT-4o, Claude Sonnet 4.5, DeepSeek-V3.2, DeepSeek-R1, and Gemini 2.5 Pro. API based MCQ queries were conducted on Jan 10, 2026, using paid API access, and the MCQs were submitted in their original language. Temperature, top-p, and maximum output tokens were not explicitly specified in the API requests and were therefore governed by each provider’s default settings at the time of access. For the reliability and readability analyses, responses to the 20 public education questions were collected through the web interfaces of ChatGPT-5, Claude Sonnet 4.5, DeepSeek-V3.2, and Gemini 2.5 Pro to approximate typical lay-user interactions. All reliability and readability prompts and responses were in English. For web interface access, temperature, top-p, and maximum output length were not directly user-adjustable and therefore remained at platform default settings. For both API based and web based queries, identical question wording was used across models within each analysis. No additional role prompts, system prompts, hyperlinks, uploaded files, images, or supplementary context were provided. Conversation history was cleared before each independent query or query session, and the first complete response generated by each model was recorded for analysis.

### 2.2 Data collection and analysis of validity

A dataset of 200 single best answer multiple choice questions (MCQs) on postpartum depression was curated by experienced obstetricians from MedQA, MedMCQA, and Chinese National Medical Examination resources (13). The questions covered four domains: presentation and severity stratification, diagnosis and clinical assessment, therapeutic approaches, and prevention, screening, and continuum of care management. Each question had one pre-specified correct answer based on the original source key.

For the MCQ based validity analysis, each MCQ was presented in the same order to six AI chatbots. All questions were presented in their original language to avoid translation related ambiguity, and identical wording and answer choices were used for all models. These MCQ responses were used only for the validity analysis based on correctness and were not included in the reliability or readability analyses. Each MCQ was presented in the same order to six AI chatbots: ChatGPT-5, ChatGPT-4o, DeepSeek-V3.2, DeepSeek-R1, Gemini 2.5 Pro, and Claude Sonnet 4.5. A standardized prompting protocol was used, with no additional context, hyperlinks, or images.

To account for stochastic variability, each model was queried three independent times for each question, with the conversation history cleared before each query. The primary outcome was accuracy, defined as the proportion of correctly answered questions.

### 2.3 Core public-education questions on PPD

To capture common public information needs related to postpartum depression (PPD), we developed a set of core public education questions using Google Trends data and structured expert review. The initial search terms were derived from the MeSH term “postpartum depression” and its entry terms, including “postnatal depression”, “postnatal dysphoria”, and “postpartum dysphoria”. These terms were searched in Google Trends under the unified topic “postpartum depression”, which aggregates conceptually equivalent search terms across languages to reflect global search interest (14). The search was set to worldwide coverage from 2020 to 2025, and the data were accessed in January 2026, and the exported query list is provided in **Supplementary Table 2**.

The 25 most popular related Google Trends queries were exported for screening. The raw queries were processed in three steps. First, exact duplicates, spelling variants, and semantically overlapping terms were merged. Second, broad or nonspecific terms were not used verbatim; they were retained only when they reflected common public concerns that could be clearly reformulated into PPD specific patient education questions. For example, the broad query “anxiety” was incorporated into a PPD specific question on whether a prior history of depression or anxiety increases PPD risk, rather than being treated as a general anxiety question. Similarly, “signs of depression” was reformulated into questions on typical PPD symptoms, early warning signs, and family recognition of PPD. The term “pregnancy depression” was considered in the perinatal context and mapped to questions addressing prior depression or anxiety and recurrence or prevention in future pregnancies. Third, to address clinically important gaps not captured by search popularity, additional questions were introduced during expert review, including definition, symptoms, risks, diagnosis, differentiation from related postpartum conditions, effects on breastfeeding and infant development, treatment, prevention, family involvement, and indications for professional help.

A panel of senior obstetricians reviewed the screened queries and drafted the final public education questions (**Table 1**). The selection criteria were: (i) prominence in Google Trends; (ii) relevance to PPD specific public information needs; (iii) clinical importance and safety relevance; (iv) clarity for lay readers; and (v) avoidance of redundancy. Disagreements regarding inclusion, exclusion, or wording were resolved through consensus discussion among the reviewers. No formal Delphi or nominal group consensus process was used. The final set consisted of 20 patient oriented core questions. The raw Google Trends queries, screening decisions, rationales, and corresponding final questions are provided in **Supplementary Table 2**.

**Table 1:** Twenty core public education questions on postpartum depression.

Number	Question
1	What is postpartum depression?
2	What are the typical symptoms of postpartum depression?
3	What are the hazards of postpartum depression?
4	When does postpartum depression usually start? How long does postpartum depression last?
5	What are the risk factors for postpartum depression?
6	How do you tell postpartum blues from postpartum depression?
7	Does a prior history of depression or anxiety increase PPD risk?
8	What are early warning signs of postpartum depression?
9	How can partners/family recognize signs of postpartum depression?
10	Can fathers/partners experience postpartum depression too?
11	Can postpartum depression affect lactation or breastfeeding success?
12	Can postpartum depression affect a baby’s development?
13	What treatments are available for postpartum depression (therapy/meds/combined)?
14	Will I get PPD with a future pregnancy, and how can I prevent it?
15	Do exercise, sunlight, diet, and social support help with PPD?
16	What can family do most effectively to prevent and treat postpartum depression?
17	When should a patient with postpartum depression seek professional help?
18	Can postpartum depression go away without treatment?
19	Can postpartum depression relapse after stopping meds, and what’s the recurrence risk?
20	How do you differentiate postpartum depression from postpartum PTSD—and can they co-occur?

The reliability and readability analyses were restricted to the four latest models listed above. Each question was submitted verbatim to four AI chatbots: ChatGPT-5, Claude Sonnet 4.5, DeepSeek-V3.2, and Gemini 2.5 Pro. No additional instructions, role prompts, or supplementary context were provided. Full text responses were collected verbatim for subsequent reliability and readability analyses. The complete chatbot outputs used for these analyses, including responses from ChatGPT-5, Claude Sonnet 4.5, DeepSeek-V3.2, and Gemini 2.5 Pro to all 20 public education questions, are provided in Supplementary File. Each question was queried once per model to approximate a typical single user interaction, and browser data were cleared before each query session to minimize personalization or prior interaction bias. The 20 public education questions were formulated and submitted in English. All chatbot responses collected for the reliability and readability analyses were also in English. No non-English responses were included, translated, or analyzed separately for readability assessment.

### 2.4 Analysis of reliability

The reliability of the chatbot generated responses was assessed using four established instruments: DISCERN, the Ensuring Quality Information for Patients (EQIP) tool, the Global Quality Scale (GQS), and the Journal of the American Medical Association (JAMA) benchmark criteria.

DISCERN is a validated instrument for evaluating the quality of written health information, particularly information related to treatment options (15). Although DISCERN was originally developed to evaluate written consumer health information on treatment choices, we applied it to all 20 public education responses for two reasons. First, several PPD public education questions were directly or indirectly related to management, treatment, prevention, relapse, help seeking, and family involvement, and even responses to broader questions such as symptoms, risk factors, and harms often included guidance on when to seek professional care or how to manage PPD related concerns. Second, applying the same instrument across all questions allowed standardized comparison of response quality between models. However, because not all questions were specifically treatment focused, DISCERN scores were interpreted as comparative indicators of health information quality within this study rather than as definitive measures of treatment information quality for every individual question. Consistent with previous studies, total DISCERN scores were interpreted using commonly adopted categories: 63–75, excellent; 51–62, good; 39–50, fair; 27–38, poor; and 16–26, very poor quality (16).

EQIP is designed to assess patient information materials, focusing on clarity, completeness, and writing quality (17). The instrument contains 20 items rated as “yes,” “partly,” “no,” or “not applicable.” For analysis, “yes,” “partly,” and “no” responses were assigned values of 1, 0.5, and 0, respectively. The summed score was converted into a percentage after adjustment for items marked as “not applicable” (17). In accordance with the original validation study, mean EQIP percentages were reported for each response and classified as follows: 76–100%, well written/excellent; 51–75%, good with minor limitations; 26–50%, substantial quality concerns; and 0–25%, severe quality deficiencies (18).

GQS provides an overall user oriented assessment of online health information, considering usefulness, coherence, readability, and practical value for end users (19). It uses a 5 points Likert scale, with scores of 1, 2, 3, 4, and 5 corresponding to very poor, poor, fair, good, and excellent quality, respectively.

JAMA benchmark criteria evaluate basic transparency and accountability of health information, including authorship, attribution or references, currency, and disclosure of ownership or sponsorship (20). Each criterion was scored as 0 if absent and 1 if present, producing a total score from 0 to 4.

Before scoring, all chatbot responses to the 20 public education questions were anonymized by removing chatbot names and any platform identifying information. Each response was assigned a coded identifier that did not reveal the chatbot identity. The anonymized responses were then randomized before being provided to the two independent obstetrician raters. The raters were blinded to chatbot identity during the initial scoring process. Each rater independently evaluated all responses using DISCERN, EQIP, GQS, and JAMA criteria. After completion of the independent ratings, discrepancies were identified by the study coordinator. The two raters then discussed discrepant items to reach consensus. If consensus could not be reached, a third senior obstetrician reviewed the anonymized response and the two initial ratings and made the final adjudication. Inter rater reliability was calculated using the initial independent ratings before consensus discussion or adjudication.

For total scores from DISCERN, EQIP, GQS, and JAMA, intraclass correlation coefficients were computed using a two ways random effects model for absolute agreement, ICC(A,1) (13, 21). For the four binary JAMA items, Cohen’s kappa was also calculated. When a JAMA item showed no variability across responses, kappa could not be estimated, and observed agreement was reported instead. Inter rater reliability was high for all instruments: DISCERN ICC = 0.977, 95% CI 0.958–0.989; EQIP ICC = 0.881, 95% CI 0.773–0.953; GQS ICC = 0.873, 95% CI 0.754–0.960; and JAMA ICC = 1.000, 95% CI 1.000–1.000. For JAMA items with identical ratings across all responses, Cohen’s kappa was not estimable, but observed agreement was 100%.

### 2.5 Analysis of readability

The readability of chatbot generated responses was evaluated using six established readability indices: the Automated Readability Index (ARI), Coleman–Liau Index (CL), Flesch–Kincaid Grade Level (FKGL), Flesch Reading Ease score (FRES), Gunning Fog Index (GFI), and Simple Measure of Gobbledygook (SMOG) index. All readability scores were calculated using an online readability tool. Because the readability indices used in this study are primarily designed and validated for English language text, the readability analysis was restricted to English chatbot responses generated from the 20 public education questions. MCQ responses in their original languages were not assessed for readability.

FRES is scored from 0 to 100, with higher values indicating greater ease of reading. By contrast, FKGL, ARI, CL, GFI, and SMOG estimate the approximate U.S. school grade level required to understand the text. Using multiple readability metrics allowed comparison of linguistic complexity across chatbot outputs and assessment of their accessibility for lay individuals seeking health information.

To interpret readability, we referred to the sixth grade reading level commonly recommended by the American Medical Association and the National Institutes of Health for patient facing health materials. Accordingly, responses were considered adequately readable if they achieved a FRES score of ≥80.0 and had grade level scores ≤ 6 on all five grade-based indices: FKGL, ARI, CL, GFI, and SMOG.

### 2.6 Statistical analysis

For the MCQ based validity analysis, each chatbot response was classified as correct or incorrect according to the prespecified answer key. Accuracy was summarized descriptively as the percentage of correctly answered items for each model across the three independent attempts. Because the MCQ outcome was binary and the same set of questions was answered repeatedly by multiple models, between model differences in MCQ accuracy were assessed using mixed effects logistic regression rather than one way ANOVA. Response correctness was specified as the binary dependent variable, chatbot model and attempt number were included as fixed effects, and question ID was included as a random intercept to account for repeated evaluation of the same questions across models and differences in item difficulty. Overall model effects were assessed using likelihood ratio tests comparing the full model with a reduced model without the chatbot model term. When pairwise comparisons were performed, estimated marginal means were compared using Tukey’s multiple comparison adjustment. The same modeling strategy was applied to the overall MCQ dataset and to each domain specific subset.

For reliability outcomes, including DISCERN, EQIP, GQS, and JAMA scores, and readability outcomes, including ARI, CL, FKGL, FRES, GFI, and SMOG, means and standard deviations were calculated for each chatbot. The Shapiro–Wilk test was used to examine whether score distributions followed normality assumptions. As several variables were nonnormally distributed, between model comparisons for these outcomes were performed using the Kruskal–Wallis test. When the overall test was significant, *post hoc* pairwise comparisons between chatbots were performed using Dunn’s multiple comparison test.

To assess whether chatbot generated responses met the sixth grade readability level recommended by the American Medical Association and the National Institutes of Health, each readability index was compared with its corresponding benchmark using the Wilcoxon signed rank test. The predefined thresholds were FRES ≥ 80.0 and scores ≤ 6 for the five grade level indices. Six tests were conducted within each model, one for each readability metric, and the Holm method was applied to adjust for family wise error.

For inter-rater reliability, ICC(A,1) was calculated for each total score, and 95% confidence intervals were estimated using bootstrap resampling with 5,000 iterations. All analyses were performed in R version 4.5.0. Two-sided p values < 0.05 were considered statistically significant.

## 3 Results

### 3.1 Validity of AI chatbots on PPD multiple-choice questions

**Figure 2:**
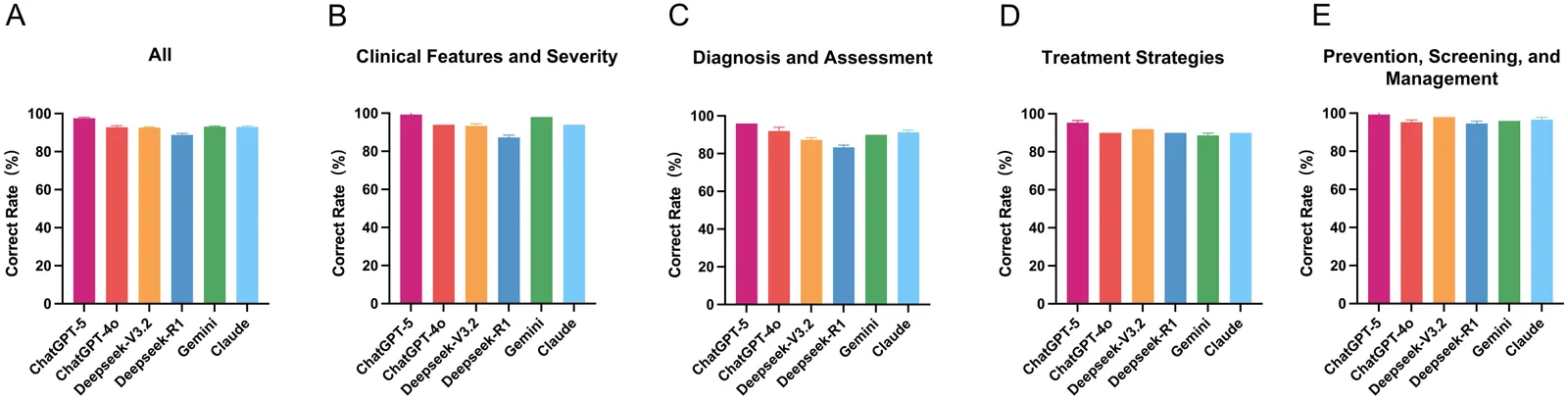
Accuracy of six AI chatbots in the MCQ-based validity analysis of postpartum depression questions. **(A)** Overall accuracy across all 200 questions for six chatbots. **(B)** Accuracy for Clinical Features and Severity (50 questions). **(C)** Accuracy for Diagnosis and Assessment (50 questions). **(D)** Accuracy for Treatment Strategies (50 questions). **(E)** Accuracy for Prevention, Screening, and Continuum of Care Management (50 questions). Data represent mean ± SD across three independent attempts per question. Bars represent the mean proportion of correctly answered questions (%) across three independent runs per item for each model; error bars indicate standard deviation. P values were derived from mixed effects logistic regression models with response correctness as the binary outcome, chatbot model and attempt number as fixed effects, and question ID as a random intercept. Significance levels are denoted as *p < 0.05, **p < 0.01, ***p < 0.001, and ****p < 0.0001.

In the MCQ based validity analysis, six AI chatbots were assessed using 200 PPD related MCQs across four domains, with 50 questions in each domain and three independent attempts per question. Overall, ChatGPT-5 achieved the highest accuracy at 97.50% ± 0.50%, followed by Gemini 2.5 Pro, Claude Sonnet 4.5, ChatGPT-4o, DeepSeek-V3.2, and DeepSeek-R1, with accuracies of 93.17% ± 0.29%, 93.00% ± 0.50%, 92.83% ± 0.76%, 92.67% ± 0.29%, and 88.83% ± 0.76%, respectively. Mixed-effects logistic regression showed a significant overall difference in MCQ accuracy among models (p < 0.0001) (**Table 2**, **Figure 2A**).

**Table 2:** Accuracy of AI chatbots in questions on postpartum depression.

Program	ChatGPT-5	ChatGPT-4o	DeepSeek-V3.2	DeepSeek-R1	Gemini	Claude	P-value
All	97.50 ± 0.50	92.83 ± 0.76	92.67 ± 0.29	88.83 ± 0.76	93.17 ± 0.29	93.00 ± 0.50	<0.0001
Clinical Features and Severity	99.33 ± 1.15	94.00 ± 0.00	93.33 ± 1.15	87.33 ± 1.15	98.00 ± 0.00	94.00 ± 0.00	<0.0001
Diagnosis and Assessment	96.00 ± 0.00	92.00 ± 2.00	87.33 ± 1.15	83.33 ± 1.15	90.00 ± 0.00	91.33 ± 1.15	<0.0001
Treatment Strategies	95.33 ± 1.15	90.00 ± 0.00	92.00 ± 0.00	90.00 ± 0.00	88.67 ± 1.15	90.00 ± 0.00	0.0928
Prevention, Screening, and Management	99.33 ± 1.15	95.33 ± 1.15	98.00 ± 0.00	94.67 ± 1.15	96.00 ± 0.00	96.67 ± 1.15	0.0357

Across the four domains, ChatGPT-5 showed the highest accuracy in clinical features and severity (99.33% ± 1.15%), diagnosis and assessment (96.00% ± 0.00%), treatment strategies (95.33% ± 1.15%), and prevention, screening, and management (99.33% ± 1.15%). Significant between model differences were observed in clinical features and severity (p < 0.0001), diagnosis and assessment (p < 0.0001), and prevention, screening, and continuum of care management (p = 0.0357). However, the between model difference in treatment strategies did not reach statistical significance (p = 0.0928) (**Table 2**, **Figures 2B–E**). The marginal means of pairwise comparisons of MCQ accuracy estimated by six AI chatbots based on the mixed-effects logistic regression model are shown in **Supplementary Table 3**.

When comparing successive model versions, ChatGPT-5 outperformed ChatGPT-4o in overall accuracy by 4.67 percentage points, and DeepSeek-V3.2 outperformed DeepSeek-R1 by 3.83 percentage points. Similar improvements were observed across most, but not all, domain specific analyses. Overall, the six chatbots demonstrated high accuracy in answering standardized PPD related MCQs, with ChatGPT-5 showing the most accurate and consistent performance in this constrained task format. However, MCQ performance should be interpreted as a measure of factual recognition under standardized conditions rather than as evidence of safe or appropriate patient facing counseling.

### 3.2 Reliability analysis

**Figure 3:**
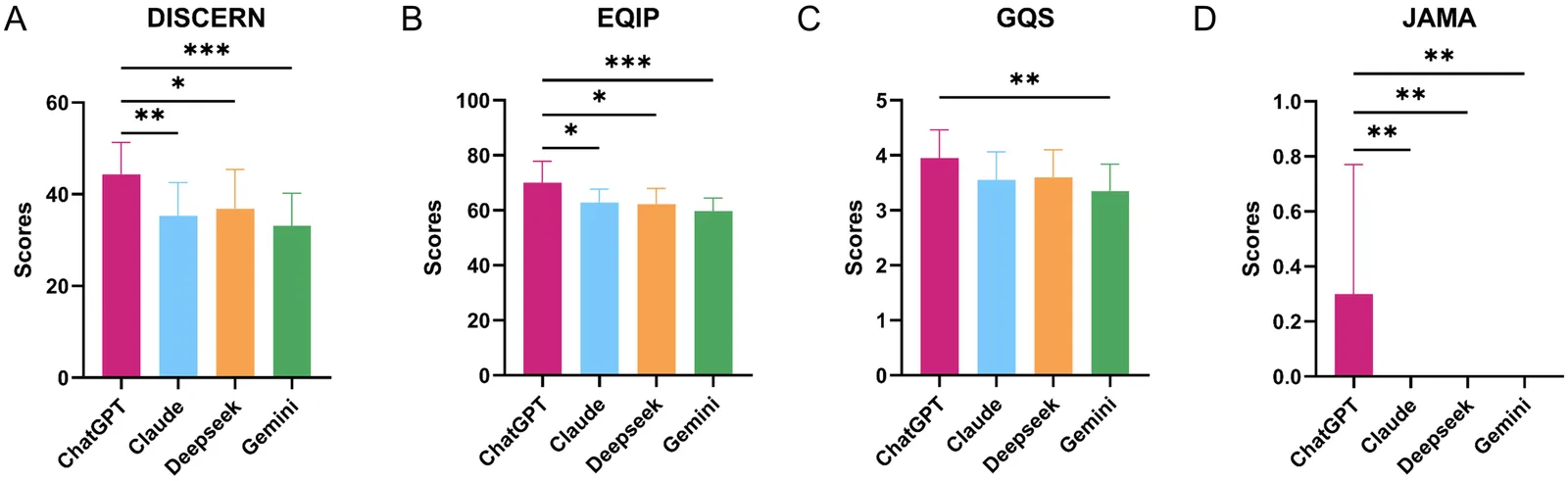
Reliability scores of the four latest AI chatbots evaluated using the 20 core public education questions. DISCERN, EQIP, GQS, and JAMA scores are shown for ChatGPT-5, Claude Sonnet 4.5, DeepSeek-V3.2, and Gemini 2.5 Pro. Bars represent mean ± SD, and adjusted p-values indicate statistical comparisons. Higher scores denote greater reliability and quality of responses. Bars represent mean scores across the 20 questions for each chatbot; error bars indicate standard deviation. Overall p values are derived from Kruskal–Wallis tests comparing the four chatbots for each instrument. When the overall test was significant, *post hoc* pairwise comparisons were performed using Dunn’s multiple-comparison test. Statistically significant pairwise differences are indicated in the figure. And statistically significant differences are indicated by asterisks: *p < 0.05, **p < 0.01, ***p < 0.001 and ****p < 0.0001.

The reliability of responses generated by four AI chatbots, ChatGPT-5, Claude Sonnet 4.5, DeepSeek-V3.2, and Gemini 2.5 Pro, was evaluated using four established instruments: DISCERN, EQIP, GQS, and JAMA benchmarks (**Table 3**, **Figure 3**). Overall, ChatGPT-5 achieved the highest mean scores across all metrics (DISCERN 44.35 ± 6.98; EQIP 70.00 ± 7.78; GQS 3.95 ± 0.51; JAMA 0.30 ± 0.47), while Gemini 2.5 Pro had the lowest scores (DISCERN 33.15 ± 7.06; EQIP 59.75 ± 4.72; GQS 3.35 ± 0.49; JAMA 0.00 ± 0.00). Statistically significant differences were observed among the four models for DISCERN, EQIP, and JAMA (p < 0.001) and for GQS (p = 0.007).

**Table 3:** Reliability scores across AI chatbots.

Program	DISCERN (mean ± SD)	EQIP (mean ± SD)	GQS (mean ± SD)	JAMA (mean ± SD)
ChatGPT	44.35 ± 6.98	70.00 ± 7.78	3.95 ± 0.51	0.30 ± 0.47
Claude Sonnet	35.30 ± 7.25	62.75 ± 4.99	3.55 ± 0.51	0.00 ± 0.00
DeepSeek	36.85 ± 8.51	62.25 ± 5.73	3.60 ± 0.50	0.00 ± 0.00
Gemini	33.15 ± 7.06	59.75 ± 4.72	3.35 ± 0.49	0.00 ± 0.00
P-value	<0.001	<0.001	0.007	<0.001

Pairwise comparisons indicated that ChatGPT-5 significantly outperformed Claude Sonnet 4.5, DeepSeek-V3.2, and Gemini 2.5 Pro on DISCERN and EQIP scores (adjusted p ≤ 0.0411), and achieved higher GQS scores compared with all three models (p = 0.0021 for each). For JAMA, ChatGPT-5 only showed a significant advantage over Gemini 2.5 Pro (p = 0.0036), while differences with Claude Sonnet 4.5 and DeepSeek-V3.2 were not statistically significant. No significant differences were found among Claude Sonnet 4.5, DeepSeek-V3.2, and Gemini 2.5 Pro across any reliability metric.

**Figure 4:**
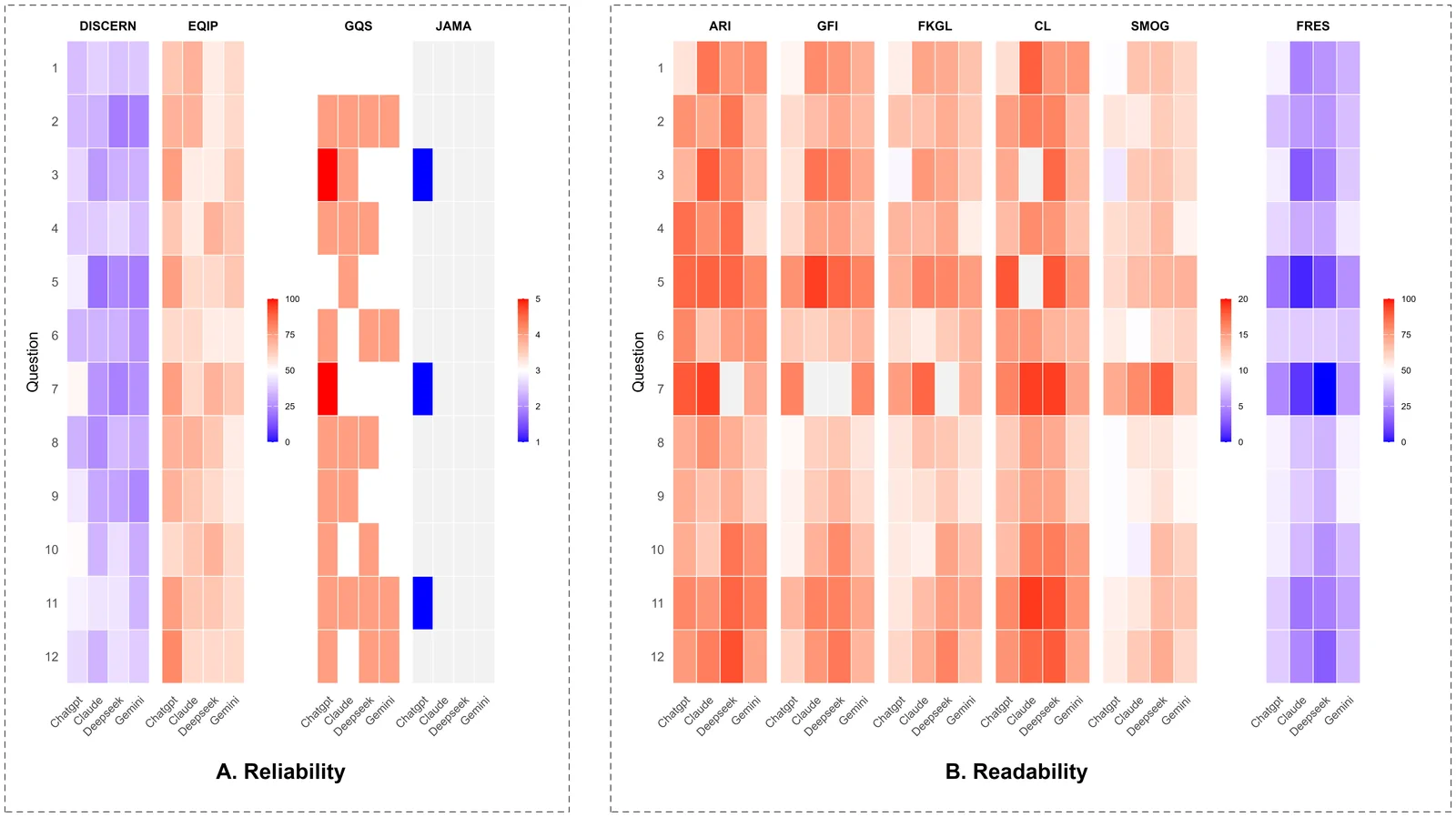
Item-level heatmaps for reliability **(A)** and readability **(B)** across the 20 cores public education questions in the four latest AI chatbots. For each question (rows Q1–Q20), tiles display clinician-assigned scores for DISCERN, EQIP, GQS, and JAMA for four chatbots (x-axis order within each block: ChatGPT-5, Claude Sonnet 4.5, DeepSeek-V3.2, Gemini 2.5 Pro). Colors correspond to the native range of each instrument as indicated by the side color bars, with warmer colors representing higher scores. Ratings were performed independently by two obstetric clinicians, and discrepancies were resolved through discussion and adjudication. **(B)** Readability. For the same questions, tiles show ARI, CL, FKGL, FRES, GFI, and SMOG metrics computed from each chatbot’s response (x-axis order as above). Warmer colors indicate higher numeric values. Interpretation: for ARI, CL, FKGL, GFI, and SMOG, higher values indicate a higher reading grade level and lower accessibility, whereas for FRES, higher values indicate easier-to-read text (0–100 scale). These heatmaps illustrate per-item variability and patterns that contribute to the overall reliability and readability findings presented in the bar plots.

Item level analysis, visualized in **Figure 4A**, revealed that ChatGPT-5 consistently received higher reliability scores across nearly all 20 cores public-education questions, particularly in DISCERN and EQIP metrics, indicating more uniform quality in patient-oriented information. In contrast, Claude Sonnet 4.5, DeepSeek-V3.2, and Gemini 2.5 Pro exhibited greater variability across individual questions, with several items scoring relatively low, suggesting inconsistent reliability in addressing certain topics.

Overall, ChatGPT-5 achieved the highest scores on DISCERN, EQIP, and GQS, suggesting relatively better content quality and user oriented usefulness within the selected evaluation framework. However, response quality varied by model and topic; therefore, higher average scores should not be interpreted as uniformly reliable performance across all PPD related questions, particularly in the absence of adequate source attribution and transparency. In addition, because DISCERN was applied across both treatment related and non-treatment specific questions, DISCERN results should be interpreted as comparative indicators of response quality rather than definitive measures of treatment information quality for every question. The JAMA benchmark results further highlight the need for cautious interpretation. Claude Sonnet 4.5, DeepSeek-V3.2, and Gemini 2.5 Pro scored 0.00 on the JAMA criteria, while ChatGPT-5 also showed a very low JAMA score. These results do not indicate complete failure in terms of factual content or practical usefulness; rather, they indicate that the generated responses generally did not satisfy the four JAMA transparency criteria: authorship, attribution or references, currency, and disclosure. Thus, the low JAMA scores suggest inadequate transparency and accountability rather than complete absence of informational value. Taken together, the reliability findings should be interpreted cautiously.

### 3.3 Readability analysis

**Figure 5:**
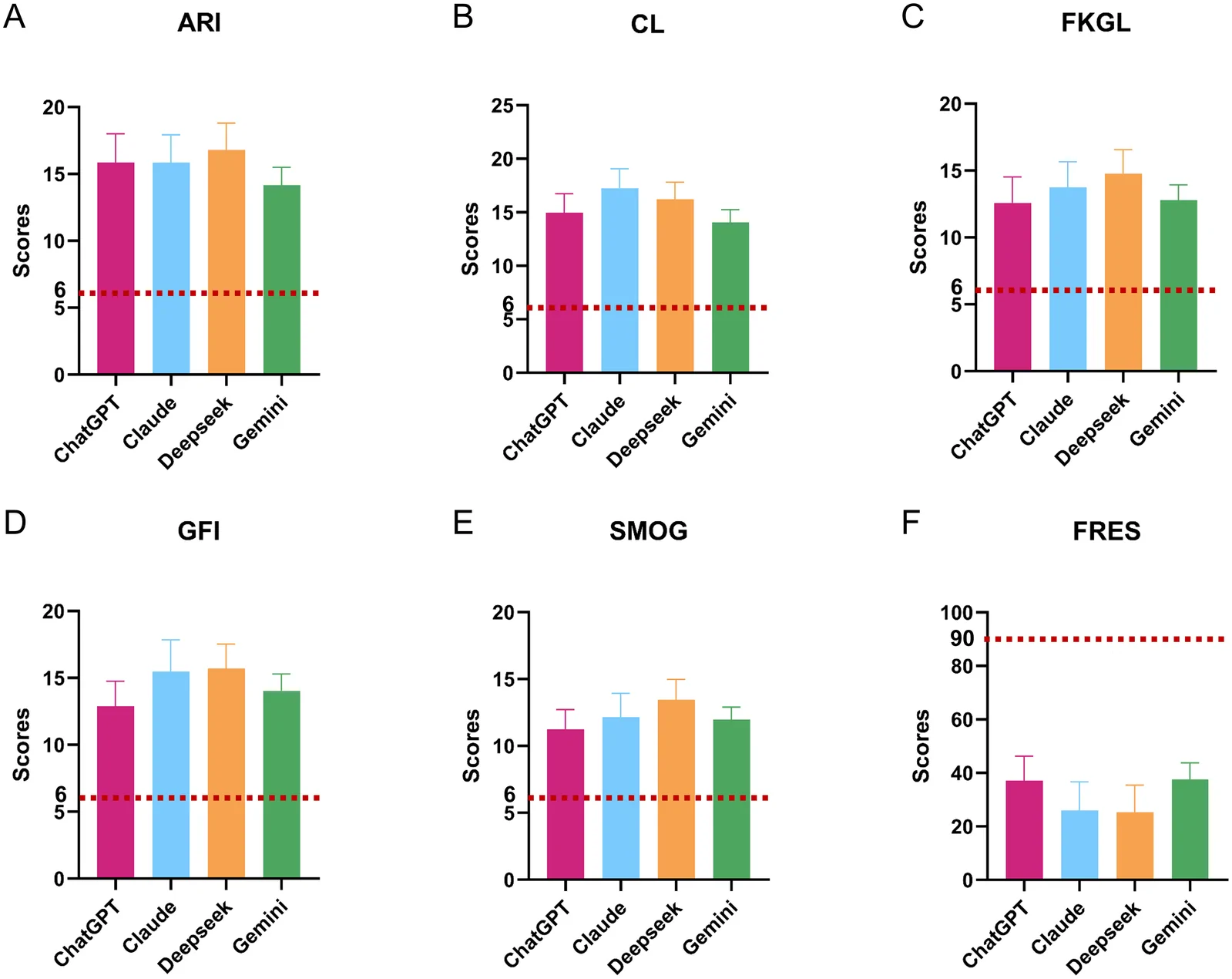
Readability comparison of the four latest AI chatbots across six metrics (ARI, CL, FKGL, FRES, GFI, SMOG) for postpartum depression content. Scores are derived from responses to the 20 core PPD public education questions. Bars represent mean values for each chatbot; error bars indicate standard deviation. For ARI, CL, FKGL, GFI and SMOG, higher values indicate a higher estimated reading grade level and lower accessibility, whereas for FRES, higher values indicate easier text (0–100scale). Overall differences among models were assessed using the Kruskal–Wallis test. When the overall test was significant, *post hoc* pairwise comparisons were performed using Dunn’s multiple comparison test. Statistically significant pairwise differences are indicated in the figure.

The readability of responses from four AI chatbots, ChatGPT-5, Claude Sonnet 4.5, DeepSeek-V3.2, and Gemini 2.5 Pro, was assessed using six metrics: ARI, CL, FKGL, FRES, GFI, and SMOG (**Table 4**, **Figure 5**). Across all models, mean readability scores exceeded the recommended sixth-grade reading level for patient-facing materials (FRES ≥ 80; grade-level indices ≤ 6), indicating that outputs were substantially more complex than advised for lay audiences.

**Table 4:** Readability scores across AI chatbots.

Program	ARI (mean ± SD)	CL (mean ± SD)	FKGL (mean ± SD)	FRES (mean ± SD)	GFI (mean ± SD)	SMOG (mean ± SD)
ChatGPT-5	15.85 ± 2.16	14.96 ± 1.78	12.59 ± 1.93	37.10 ± 9.17	12.89 ± 1.88	11.26 ± 1.47
Claude Sonnet 4.5	15.84 ± 2.07	17.26 ± 1.82	13.75 ± 1.91	26.00 ± 10.71	15.50 ± 2.35	12.15 ± 1.79
DeepSeek -V3.2	16.80 ± 1.99	16.21 ± 1.62	14.77 ± 1.81	25.30 ± 10.18	15.71 ± 1.83	13.46 ± 1.51
Gemini 2.5 Pro	14.15 ± 1.35	14.05 ± 1.19	12.79 ± 1.15	37.50 ± 6.30	14.04 ± 1.26	11.98 ± 0.94
6th grade level score	6	6	6	≥80	6	6

ChatGPT-5 and Gemini 2.5 Pro produced comparatively more readable text (FRES 37.10 ± 9.17 and 37.50 ± 6.30), whereas Claude Sonnet 4.5 and DeepSeek-V3.2 generated more complex outputs (FRES 26.00 ± 10.71 and 25.30 ± 10.18). Significant differences were observed across all metrics (Kruskal–Wallis test, p < 0.001). Overall p values were derived from Kruskal–Wallis tests comparing the four chatbots for each readability metric. When the overall test was significant, *post hoc* pairwise comparisons were performed using Dunn’s multiple-comparison test.

Item level analysis (**Figure 4B**) showed that ChatGPT-5 and Gemini 2.5 Pro maintained more consistent readability across the 20 core questions, while Claude Sonnet 4.5 and DeepSeek-V3.2 displayed greater variability, with some questions producing particularly complex text. Overall, none of the models met the sixth grade readability benchmark recommended by the AMA and NIH, although ChatGPT-5 and Gemini 2.5 Pro were relatively more accessible than Claude Sonnet 4.5 and DeepSeek-V3.2. Importantly, “relatively more accessible” should not be interpreted as adequately readable for patient centered education, because all models still generated text above the recommended reading level.

## 4 Discussion

In this study, we systematically evaluated AI chatbots answering PPD related questions across validity, reliability, and readability. Six models were included in the MCQ based validity analysis, while ChatGPT 5, Claude Sonnet 4.5, DeepSeek V3.2, and Gemini 2.5 Pro were evaluated for reliability and readability. Consistent with findings in other medical domains, ChatGPT 5 demonstrated higher MCQ accuracy and higher scores on several content quality measures than earlier or less optimized models. Similar patterns have been observed in previous assessments of AI chatbots providing sexual health and STD information, where certain models outperformed others in reliability and content quality metrics, highlighting the influence of model architecture and training data on output quality (22–24).

Our reliability results showed that ChatGPT-5 achieved higher DISCERN, EQIP, and GQS scores than the other models, whereas the other models exhibited greater variability across items (23, 25, 26). However, these findings should be interpreted cautiously. The JAMA benchmark results highlight an important limitation: JAMA criteria primarily assess transparency and accountability, including authorship, attribution or references, currency, and disclosure. In our study, JAMA scores were very low across all models, indicating that chatbot generated responses generally did not provide sufficient source attribution, update information, or disclosure statements. Therefore, higher scores on DISCERN, EQIP, or GQS should not be interpreted as evidence that the responses were fully reliable or transparent. Rather, ChatGPT-5 showed relatively better content quality and practical usefulness within the selected evaluation framework, while all evaluated models remained limited in transparency and traceability. This limitation is particularly important in postpartum mental health education, where users may need evidence based, up to date, and clinically accountable information.

The finding that Claude Sonnet 4.5, DeepSeek-V3.2, and Gemini 2.5 Pro scored 0.00 on the JAMA benchmark should not be interpreted as complete failure across all evaluation dimensions. JAMA criteria evaluate transparency and accountability rather than the factual accuracy, completeness, or readability of the response itself. A score of 0 indicates that the response did not explicitly provide authorship information, cite sources or references, state the currency or update date of the information, or disclose ownership, sponsorship, or related accountability information. In contrast, the same models achieved nonzero DISCERN, EQIP, and GQS scores, indicating that they still provided some clinically relevant content and user oriented information. Thus, the main implication of the JAMA findings is not that these chatbots produced no useful information, but that their responses lacked traceability and source accountability. This distinction is important because users may be unable to verify where the information came from, whether it reflects current evidence, or whether professional clinical guidance is needed. In postpartum depression education, this limitation is clinically relevant because outdated, unsourced, or non-transparent information may affect help seeking, medication related decisions, and recognition of urgent mental health risks.

The topic dependent variability observed across models also has implications for user trust. The finding that some models produced lower or more variable quality scores across individual questions does not mean that all chatbot generated information is unusable. However, it does mean that users should not fully rely on chatbot responses as autonomous or definitive sources of postpartum mental health information. These tools may be cautiously used as supplementary starting points for low risk, general educational purposes, such as learning about the broad definition of postpartum depression, common symptoms, general risk factors, and the importance of seeking help. Even in these situations, information should ideally be checked against clinician reviewed materials or discussed with healthcare professionals.

In contrast, chatbot responses should not be considered sufficiently trustworthy for individualized clinical decision making, diagnosis, treatment selection, medication use or discontinuation, breastfeeding related medication safety, assessment of relapse risk, or evaluation of suicidal ideation, self-harm risk, harm to the infant, psychosis, or severe functional impairment. These situations require professional assessment, individualized risk evaluation, and timely clinical intervention. Therefore, trust in chatbot generated PPD information should be conditional and context specific: chatbots may assist with general awareness and question preparation, but they should not replace professional assessment, especially when symptoms are severe, ambiguous, urgent, or personally specific.

Readability analysis in our study revealed that none of the evaluated chatbots met the sixth grade reading level recommended for patient education by health organizations such as the AMA and NIH. This finding echoes results from other evaluations of AI chatbots in healthcare, where generated text often exceeded recommended readability thresholds, potentially limiting accessibility for lay audiences. For example, analyses of AI chatbot outputs in gastroenterological and dental health information reported complexity levels above recommended standards, suggesting that current models may not yet fully optimize for patient-friendly language without specific prompt engineering or simplification strategies (27, 28).

The failure of all evaluated models to meet the sixth grade readability benchmark has important implications for accessibility. Although ChatGPT-5 and Gemini 2.5 Pro were relatively more readable than Claude Sonnet 4.5 and DeepSeek-V3.2, their outputs still corresponded approximately to upper secondary or college level reading demands and therefore remained more complex than recommended for patient centered materials. This may substantially limit accessibility for users with basic education, limited health literacy, non-native English proficiency, or reduced digital literacy. The impact may be particularly important for postpartum women and family members facing a digital divide, because these users may have less ability to judge medical uncertainty, identify missing safety information, recognize when professional help is needed, or request simplified follow-up explanations. In the context of postpartum depression, overly complex AI generated responses may reduce comprehension, increase misunderstanding, and delay appropriate help seeking, especially when users are experiencing fatigue, emotional distress, anxiety, or crisis related concerns. Therefore, current chatbot outputs should not be assumed to be accessible to all lay users without explicit simplification strategies, readability optimization, or clinician reviewed patient education materials.

Our results reinforce the broader literature indicating that, although modern AI chatbots can provide accurate medical information, the quality and readability of this information vary widely by model and domain. Notably, studies evaluating AI chatbot responses to common patient questions about zirconia crowns found disparities in accuracy, reliability, and readability across systems, similar to the patterns we observed in PPD related content (23, 24, 29). Taken together, these findings suggest that AI chatbots hold promise as supplementary tools for disseminating health information but should be used with caution and ideally complemented by expert review or content tailoring for specific patient populations.

Several methodological limitations should be acknowledged. First, although mixed effects logistic regression accounted for the binary and repeated measures structure of the MCQ data, MCQ based validity testing remains a constrained assessment of factual recognition. High performance on single best answer questions does not necessarily indicate that a chatbot can provide safe, appropriate, or individualized patient facing counseling. Real world PPD queries may involve clinical ambiguity, emotional distress, medication concerns, or crisis risk, which require individualized assessment, empathy, triage, and professional judgment. Therefore, the MCQ findings should be interpreted as performance on structured knowledge assessment rather than evidence of clinical safety or real world effectiveness. Second, the development of the 20 public education questions involved potential selection bias. Google Trends reflects public search interest rather than clinical priority or content validity, and some broad queries were reformulated into PPD specific questions through expert review. Although this improved clinical relevance, it may have introduced subjective judgment. In addition, no formal Delphi process or patient and public involvement was used. Future studies should incorporate structured consensus methods and patient representative input. Third, DISCERN was applied across all 20 questions, although it was originally designed to assess consumer health information on treatment choices (15). Because some questions focused on definitions, symptoms, onset, and risk factors rather than treatment, DISCERN scores for these items should be interpreted cautiously. We therefore used DISCERN as one component of a broader evaluation framework alongside EQIP, GQS, and JAMA. Readability was also assessed only for English language responses, which limits generalizability to Chinese and other languages. Finally, this study did not include formal usability analysis or direct patient comprehension testing. Readability indices estimate textual complexity but do not determine whether users can understand, trust, or appropriately act on AI generated information. Accordingly, the finding that all models exceeded the recommended sixth grade reading level should be interpreted as an accessibility concern rather than direct evidence of user misunderstanding. Future studies should include users with diverse educational, health literacy, and digital literacy backgrounds and evaluate whether simplification prompts, plain language editing, clinician review, or patient co design can improve the accessibility and safety of AI generated postpartum mental health information.

In summary, our study extends existing evidence by demonstrating that AI chatbots differ meaningfully in performance when responding to PPD questions. While the most advanced model (ChatGPT-5) showed relatively strong performance, all models produced content that varied in reliability and was linguistically complex. These insights underscore the need for ongoing evaluation and development to ensure that AI-generated health information is both accurate and accessible.

## 5 Conclusion

This study evaluated AI chatbots on postpartum depression related questions in terms of MCQ based validity, content quality, transparency, and readability. In the six models validity analysis, ChatGPT-5 achieved the highest accuracy on standardized MCQs. In the four models public education analysis, ChatGPT-5 showed relatively better content quality scores, while the low JAMA scores of all models indicate that current chatbot responses remain limited in transparency, source attribution, currency, and disclosure, even when they provide clinically relevant information on some content quality measures.

These findings suggest that AI chatbots may have potential as supplementary tools for postpartum depression education, but they should not be considered autonomous sources of medical or mental health information. This study did not evaluate clinical safety, patient comprehension, usability, user behavior, or real-world effectiveness. In addition, the consistently high reading level of chatbot outputs indicates that default AI generated responses may not be sufficiently accessible for patient facing use without explicit simplification strategies, readability optimization, plain language editing, and expert review. Therefore, AI chatbots should not replace professional assessment, diagnosis, or treatment, particularly in the sensitive context of postpartum mental health.

Given the topic dependent variability in response quality, chatbot generated information may be useful for general awareness and question preparation but should be verified against professional or clinician reviewed sources before being used for postpartum mental health decisions.

## Data Availability

The original contributions presented in the study are included in the article/**Supplementary Material**. Further inquiries can be directed to the corresponding authors.

## References

[1] O'Hara MW McCabe JE . Postpartum depression: current status and future directions. Annu Rev Clin Psychol. (2013) 9:379–407. 23394227 10.1146/annurev-clinpsy-050212-185612

[2] Vesga-López O Blanco C Keyes K Olfson M Grant BF Hasin DS . Psychiatric disorders in pregnant and postpartum women in the United States. Arch Gen Psychiatry. (2008) 65:805–15. doi: 10.1001/archpsyc.65.7.805 18606953 PMC2669282

[3] Slomian J Honvo G Emonts P Reginster JY Bruyère O . Consequences of maternal postpartum depression: a systematic review of maternal and infant outcomes. Womens Health (Lond). (2019) 15:1745506519844044. doi: 10.1177/1745506519844044 31035856 PMC6492376

[4] Stewart DE Vigod SN . Postpartum depression: pathophysiology, treatment, and emerging therapeutics. Annu Rev Med. (2019) 70:183–96. doi: 10.1146/annurev-med-041217-011106 30691372

[5] Dennis CL Falah-Hassani K Shiri R . Prevalence of antenatal and postnatal anxiety: systematic review and meta-analysis. Br J Psychiatry. (2017) 210:315–23. doi: 10.1192/bjp.bp.116.187179 28302701

[6] Goodman JH . Women's attitudes, preferences, and perceived barriers to treatment for perinatal depression. Birth. (2009) 36:60–9. doi: 10.1111/j.1523-536x.2008.00296.x 19278385

[7] Grace SL Evindar A Stewart DE . The effect of postpartum depression on child cognitive development and behavior: a review and critical analysis of the literature. Arch Womens Ment Health. (2003) 6:263–74. doi: 10.1007/s00737-003-0024-6 14628179

[8] Cooper PJ De Pascalis L Woolgar M Romaniuk H Murray L . Attempting to prevent postnatal depression by targeting the mother-infant relationship: a randomised controlled trial. Prim Health Care Res Dev. (2015) 16:383–97. doi: 10.1017/s1463423614000401 25381790

[9] Murray L Arteche A Fearon P Halligan S Goodyer I Cooper P . Maternal postnatal depression and the development of depression in offspring up to 16 years of age. J Am Acad Child Adolesc Psychiatry. (2011) 50:460–70. doi: 10.1016/j.jaac.2011.02.001 21515195

[10] Miner AS Laranjo L Kocaballi AB . Chatbots in the fight against the COVID-19 pandemic. NPJ Digit Med. (2020) 3:65. doi: 10.1038/s41746-020-0280-0 32377576 PMC7198587

[11] Jeblick K Schachtner B Dexl J Mittermeier A Stüber AT Topalis J . ChatGPT makes medicine easy to swallow: an exploratory case study on simplified radiology reports. Eur Radiol. (2024) 34:2817–25. doi: 10.1007/s00330-023-10213-1 37794249 PMC11126432

[12] Kung TH Cheatham M Medenilla A Sillos C De Leon L Elepaño C . Performance of ChatGPT on USMLE: potential for AI-assisted medical education using large language models. PloS Digit Health. (2023) 2:e0000198. doi: 10.1371/journal.pdig.0000198 36812645 PMC9931230

[13] Koo TK Li MY . A guideline of selecting and reporting intraclass correlation coefficients for reliability research. J Chiropr Med. (2016) 15:155–63. doi: 10.1016/j.jcm.2016.02.012 27330520 PMC4913118

[14] Mavragani A Ochoa G . Google Trends in infodemiology and infoveillance: methodology framework. JMIR Public Health Surveill. (2019) 5:e13439. doi: 10.2196/13439 31144671 PMC6660120

[15] Charnock D Shepperd S Needham G Gann R . DISCERN: an instrument for judging the quality of written consumer health information on treatment choices. J Epidemiol Community Health. (1999) 53:105–11. doi: 10.1136/jech.53.2.105 10396471 PMC1756830

[16] Weil AG Bojanowski MW Jamart J Gustin T Lévêque M . Evaluation of the quality of information on the Internet available to patients undergoing cervical spine surgery. World Neurosurg. (2014) 82:e31–9. doi: 10.1016/j.wneu.2012.11.003 23142585

[17] Moult B Franck LS Brady H . Ensuring quality information for patients: development and preliminary validation of a new instrument to improve the quality of written health care information. Health Expect. (2004) 7:165–75. doi: 10.1111/j.1369-7625.2004.00273.x 15117391 PMC5060233

[18] Hain T . Improving the quality of health information: the contribution of C-H-i-Q. Health Expect. (2002) 5:270–3. doi: 10.1046/j.1369-6513.2002.00189.x 12199665 PMC5060154

[19] Mohammad-Rahimi H Ourang SA Pourhoseingholi MA Dianat O Dummer PMH Nosrat A . Validity and reliability of artificial intelligence chatbots as public sources of information on endodontics. Int Endod J. (2024) 57:305–14. doi: 10.1111/iej.14014 38117284

[20] Silberg WM Lundberg GD Musacchio RA . Assessing, controlling, and assuring the quality of medical information on the Internet: caveant lector et viewor--let the reader and viewer beware. JAMA. (1997) 277:1244–5. doi: 10.1001/jama.1997.03540390074039 9103351

[21] Shrout PE Fleiss JL . Intraclass correlations: uses in assessing rater reliability. Psychol Bull. (1979) 86:420–8. doi: 10.1002/0471234311.ch4 18839484

[22] Yildiz HA Sogutdelen E . AI chatbots as sources of STD information: a study on reliability and readability. J Med Syst. (2025) 49:43. 40178771 10.1007/s10916-025-02178-z PMC11968469

[23] Li R Zhao A Peng L Shi H Zhao J Li Z . Evaluating the clinical competence of large language models in prostate cancer management: a comparative study of DeepSeek-R1 and ChatGPT. Ann Surg Oncol. (2026) 33:1858–69. doi: 10.1245/s10434-025-18492-2 41094286

[24] Wang X Lin S Liu H Li C Zhou L Li R . Evaluation of validity, reliability, and readability of AI chatbots for gestational diabetes mellitus: a multi-model comparative study. Front Public Health. (2026) 14:1760871. doi: 10.3389/fpubh.2026.1760871 41717624 PMC12913397

[25] Demir S . Evaluation of the reliability and readability of answers given by chatbots to frequently asked questions about endophthalmitis: a cross-sectional study on chatbots. Health Inf J. (2024) 30:14604582241304679. doi: 10.1177/14604582241304679 39612357

[26] Buker M Mercan G . Readability, accuracy and appropriateness and quality of AI chatbot responses as a patient information source on root canal retreatment: a comparative assessment. Int J Med Inform. (2025) 201:105948. doi: 10.1016/j.ijmedinf.2025.105948 40288015

[27] Toiv A Saleh Z Ishak A Alsheik E Venkat D Nandi N . Digesting digital health: a study of appropriateness and readability of ChatGPT-generated gastroenterological information. Clin Transl Gastroenterol. (2024) 15:e00765. doi: 10.14309/ctg.0000000000000765 39212302 PMC11596446

[28] Latt PM Aung ET Htaik K Soe NN Lee D King AJ . Evaluation of artificial intelligence (AI) chatbots for providing sexual health information: a consensus study using real-world clinical queries. BMC Public Health. (2025) 25:1788. doi: 10.21203/rs.3.rs-5190887/v1 40375254 PMC12080182

[29] Acar NK Sengul F Bardakci E Celikel P . Assessing the accuracy, reliability, quality, and readability of artificial intelligence chatbots in patient education: insights from zirconia crowns. BMC Oral Health. (2026) 26(1):502. doi: 10.1186/s12903-026-07884-9 41680781 PMC13005407

