# Supplementary material for: Evaluating the accuracy, reliability, and readability of AI chatbots in delivering postpartum depression information
Source: Front Psychiatry. 2026 Aug 12;17:1881536. doi: 10.3389/fpsyt.2026.1881536 (PMC13506791; doi:10.3389/fpsyt.2026.1881536)
Supplement: Supplementary file 1 [file SupplementaryFile1.docx]

**Supplementary Table S1** Technical details of AI chatbot access and settings.

| Model | Analysis module | Access route | Platform/provider | Access date | Language | Account/subscription or API status | Temperature | Top-p | Maximum output tokens/response length | Conversation history |
| --- | --- | --- | --- | --- | --- | --- | --- | --- | --- | --- |
| ChatGPT-5 | Validity/MCQ | API | OpenAI API | Jan 10, 2026 | original language | paid API | Not explicitly specified; provider default | Not explicitly specified; provider default | Not explicitly specified; provider default | Cleared before each query |
| ChatGPT-4o | Validity/MCQ | API | OpenAI API | Jan 10, 2026 | original language | paid API | Not explicitly specified; provider default | Not explicitly specified; provider default | Not explicitly specified; provider default | Cleared before each query |
| Claude Sonnet 4.5 | Validity/MCQ | API | Anthropic API | Jan 10, 2026 | original language | paid API | Not explicitly specified; provider default | Not explicitly specified; provider default | Not explicitly specified; provider default | Cleared before each query |
| DeepSeek-V3.2 | Validity/MCQ | API | DeepSeek API | Jan 10, 2026 | original language | paid API | Not explicitly specified; provider default | Not explicitly specified; provider default | Not explicitly specified; provider default | Cleared before each query |
| DeepSeek-R1 | Validity/MCQ | API | DeepSeek API | Jan 10, 2026 | original language | paid API | Not explicitly specified; provider default | Not explicitly specified; provider default | Not explicitly specified; provider default | Cleared before each query |
| Gemini 2.5 Pro | Validity/MCQ | API | Google AI Studio API | Jan 10, 2026 | original language | paid API | Not explicitly specified; provider default | Not explicitly specified; provider default | Not explicitly specified; provider default | Cleared before each query |
| ChatGPT-5 | Reliability and readability | Web interface | ChatGPT web interface | Jan 13–16, 2026 | English | Paid subscription | Not user-adjustable; platform default | Not user-adjustable; platform default | Not user-adjustable; no explicit length instruction | Cleared before each query/session |
| Claude Sonnet 4.5 | Reliability and readability | Web interface | Claude.ai web interface | Jan 18-23, 2026 | English | Paid subscription | Not user-adjustable; platform default | Not user-adjustable; platform default | Not user-adjustable; no explicit length instruction | Cleared before each query/session |
| DeepSeek-V3.2 | Reliability and readability | Web interface | DeepSeek web interface | Jan 27-28, 2026 | English | Free account | Not user-adjustable; platform default | Not user-adjustable; platform default | Not user-adjustable; no explicit length instruction | Cleared before each query/session |
| Gemini 2.5 Pro | Reliability and readability | Web interface | Gemini web interface | Jan 28-30, 2026 | English | Paid subscription | Not user-adjustable; platform default | Not user-adjustable; platform default | Not user-adjustable; no explicit length instruction | Cleared before each query/session |

For API-based MCQ testing, temperature, top-p, and maximum output tokens were not explicitly specified in the API requests and were therefore governed by each provider’s default settings at the time of access. For web-interface testing, these parameters were not directly user-adjustable and therefore remained at platform default settings. The language column refers to both the prompt language and the collected response language for each analysis module.

**Supplementary Table S2** Development of the 20 public education questions from Google Trends queries and expert review.

| RANK | TOP | Relevance | **Screening decision** | **Rationale for decision or reformulation** | **Final question(s)** |
| --- | --- | --- | --- | --- | --- |
| 1 | what is postpartum depression | 100 | Retained and standardized | Directly reflects public interest in the definition of PPD | Q1 |
| 2 | what is postpartum | 99 | Reformulated and merged | Too broad and not specific to depression; narrowed to the definition of PPD | Q1 |
| 3 | postpartum depression symptoms | 84 | Retained and standardized | Directly reflects public interest in common symptoms | Q2 |
| 4 | depression symptoms | 83 | Reformulated and merged | Broad and not postpartum specific; incorporated into questions on PPD symptoms and warning signs | Q2, Q8, Q9 |
| 5 | postpartum symptoms | 83 | Reformulated and merged | Broad and potentially includes physical postpartum symptoms; narrowed to PPD symptoms and warning signs | Q2, Q8 |
| 6 | postpartum depression how long | 67 | Merged | Semantically overlaps with other queries on PPD duration | Q4 |
| 7 | postpartum meaning | 52 | Reformulated and merged | Refers broadly to the postpartum period rather than PPD; incorporated into the definition context | Q1 |
| 8 | postpartum depression meaning | 48 | Retained and standardized | Directly reflects interest in the meaning and definition of PPD | Q1 |
| 9 | how long postpartum depression last | 46 | Merged | Duplicate duration related query | Q4 |
| 10 | postpartum depression signs | 41 | Retained and reformulated | Converted into a clinically clearer question on early warning signs | Q8 |
| 11 | depression post partum | 38 | Merged | Spelling and word order variant of PPD related queries | Q1, Q2 |
| 12 | postpartum depression treatment | 37 | Retained and expanded | Directly reflects treatment related information needs; expanded to include major treatment modalities | Q13 |
| 13 | anxiety | 37 | Reformulated | Too broad to use verbatim; incorporated into a PPD specific question on previous depression or anxiety as a risk factor | Q7 |
| 14 | post partum | 36 | Reformulated and merged | Broad spelling variant referring to the postpartum period; not sufficiently specific as a standalone question | Q1 |
| 15 | postpartum depression help | 36 | Reformulated | Converted into a clinically actionable question on indications for seeking professional care | Q17 |
| 16 | postpartum anxiety | 35 | Reformulated and merged | Related to postpartum mental health but not identical to PPD; incorporated into the assessment of anxiety history as a PPD risk factor | Q7 |
| 17 | postpartum anxiety depression | 35 | Reformulated and merged | Overlapping query involving postpartum anxiety and depression; incorporated into the PPD risk context | Q7 |
| 18 | signs of postpartum depression | 35 | Merged and expanded | Overlaps with warning sign queries and was expanded to include recognition by family members | Q8, Q9 |
| 19 | pregnancy depression | 33 | Reformulated | Broad perinatal depression query; reformulated to address recurrence and prevention in future pregnancies | Q14 |
| 20 | signs of depression | 33 | Reformulated and merged | Broad and not postpartum specific; incorporated into PPD warning signs and family recognition | Q8, Q9 |
| 21 | postpartum depression news | 31 | Excluded | Time sensitive and not suitable as a stable core patient education question | Not retained |
| 22 | how long does postpartum depression last | 30 | Merged | Duplicate duration related query | Q4 |
| 23 | postpartum blues | 30 | Retained and expanded | Directly relevant to a common public concern and expanded to distinguish postpartum blues from PPD | Q6 |
| 24 | how long does postpartum last | 30 | Reformulated and merged | Refers broadly to the postpartum period; narrowed to onset and duration of PPD | Q4 |
| 25 | symptoms of postpartum depression | 28 | Merged | Duplicate symptom related query | Q2 |

The 25 Google Trends queries were screened for relevance, specificity, duplication, and suitability for patient education. Queries were retained, merged with semantically overlapping terms, reformulated to improve PPD specificity, or excluded when they were too broad, duplicative, or unsuitable as stable educational topics. Because search popularity does not necessarily capture all clinically important information needs, additional questions were introduced during expert review to address relevant gaps in risk, consequences, family involvement, breastfeeding, infant outcomes, treatment course, relapse, and differential diagnosis. The final 20 questions were agreed upon by expert consensus.

**Supplementary Table 3** Pairwise comparisons of MCQ accuracy among six AI chatbots based on estimated marginal means from mixed-effects logistic regression models.

| Adjusted P Value | Adjusted P Value | | | | |
| --- | --- | --- | --- | --- | --- |
|  | All | Clinical Features and Severity | Diagnosis and Assessment | Treatment Strategies | Prevention, Screening, and Management |
| ChatGPT-5 vs. ChatGPT-4o | <0.0001 | <0.0001 | 0.0113 | <0.0001 | 0.0024 |
| ChatGPT-5 vs. Deepseek-V3.2 | <0.0001 | <0.0001 | <0.0001 | 0.0006 | 0.538 |
| ChatGPT-5 vs. Deepseek-R1 | <0.0001 | <0.0001 | <0.0001 | <0.0001 | 0.0006 |
| ChatGPT-5 vs. Gemini | <0.0001 | 0.3955 | 0.0004 | <0.0001 | 0.0097 |
| ChatGPT-5 vs. Claude | <0.0001 | <0.0001 | 0.0035 | <0.0001 | 0.0419 |
| ChatGPT-4o vs. Deepseek-V3.2 | 0.9989 | 0.9093 | 0.0035 | 0.0294 | 0.0419 |
| ChatGPT-4o vs. Deepseek-R1 | <0.0001 | <0.0001 | <0.0001 | >0.9999 | 0.9478 |
| ChatGPT-4o vs. Gemini | 0.9728 | 0.0007 | 0.3386 | 0.2139 | 0.9478 |
| ChatGPT-4o vs. Claude | 0.9989 | >0.9999 | 0.9774 | >0.9999 | 0.538 |
| Deepseek-V3.2 vs. Deepseek-R1 | <0.0001 | <0.0001 | 0.0113 | 0.0294 | 0.0097 |
| Deepseek-V3.2 vs. Gemini | 0.8691 | 0.0002 | 0.1194 | 0.0006 | 0.1711 |
| Deepseek-V3.2 vs. Claude | 0.9728 | 0.9093 | 0.0113 | 0.0294 | 0.538 |
| Deepseek-R1 vs. Gemini | <0.0001 | <0.0001 | 0.0001 | 0.2139 | 0.538 |
| Deepseek-R1 vs. Claude | <0.0001 | <0.0001 | <0.0001 | >0.9999 | 0.1711 |
| Gemini vs. Claude | 0.9989 | 0.0007 | 0.7189 | 0.2139 | 0.9478 |
