# Supplementary material for: Evaluating the accuracy, reliability, and readability of AI chatbots in delivering postpartum depression information
Source: Front Psychiatry. 2026 Aug 12;17:1881536. doi: 10.3389/fpsyt.2026.1881536 (PMC13506791; doi:10.3389/fpsyt.2026.1881536)
Supplement: Supplementary file 2 [file SupplementaryFile2.docx]

Supplementary File: The complete chatbot outputs includs responses from ChatGPT-5, Claude Sonnet 4.5, DeepSeek-V3.2, and Gemini 2.5 Pro to all 20 public education questions.

ChatGPT

**1.What is postpartum depression?**

Postpartum depression (PPD) is a mood disorder that can happen after having a baby. It goes beyond the common “baby blues,” which many people experience in the first few days after delivery and usually improves on its own within about two weeks. PPD is more intense, lasts longer, and can make it hard to function day to day or to enjoy caring for the baby.

PPD can include emotional, physical, and thinking-related symptoms. People may feel persistently sad, empty, hopeless, irritable, or overwhelmed. Some lose interest in activities, cry often, or feel numb. Anxiety can be a major part of PPD too—constant worry, racing thoughts, or panic. Sleep and appetite may change (even when the baby is sleeping), and energy can feel very low. Many people experience guilt or worthlessness, feel like they’re a “bad parent,” or struggle to bond with the baby. Concentration problems and indecisiveness are common. Symptoms can start within the first few weeks, but they can also begin later in the first year postpartum.

PPD is not a character flaw or a sign of weakness. It is linked to a mix of factors such as hormone shifts after birth, sleep deprivation, physical recovery, stress, a history of depression or anxiety, limited support, complications in pregnancy or delivery, and major life changes.

Treatment is effective. Options include therapy (like cognitive behavioral therapy or interpersonal therapy), social support, practical help with sleep and childcare, and sometimes medication. If someone has thoughts of self-harm or harming the baby, or experiences hallucinations or severe confusion, it’s an emergency and they should seek immediate help.

**2.What are the typical symptoms of postpartum depression?**

Postpartum depression (PPD) is more than the short-lived “baby blues.” It’s a mood disorder that can start within the first few weeks after birth, but it can also begin later (even months postpartum). Symptoms vary by person, and they often overlap with ordinary postpartum exhaustion—so it’s the pattern, severity, and persistence that matter.

Typical symptoms include a persistently low or “empty” mood, frequent crying, feeling hopeless, or feeling numb and disconnected. Many people describe losing interest or pleasure in things they normally enjoy, including bonding activities with the baby. Irritability, anger, or feeling overwhelmed is common, sometimes accompanied by guilt (“I’m a bad parent”), shame, or a sense of inadequacy.

Anxiety symptoms are also frequent: constant worry about the baby’s health, racing thoughts, panic attacks, or feeling on edge. Some people experience intrusive thoughts (unwanted, upsetting thoughts or images), which can be frightening—especially if they involve harm coming to the baby. Sleep can be disrupted beyond typical newborn schedules: either insomnia even when the baby sleeps, or sleeping excessively. Appetite changes, low energy, difficulty concentrating, and slowed thinking can occur.

PPD can affect behavior and relationships: withdrawing from family/friends, avoiding childcare tasks, or having difficulty making decisions. In more severe cases, there may be thoughts of self-harm or suicide, or thoughts of harming the baby—these are medical emergencies.

If these symptoms last more than two weeks, are getting worse, or interfere with daily functioning or caring for yourself/baby, it’s important to seek professional support promptly (OB-GYN, primary care, midwife, or mental health clinician). If you’re in immediate danger or having thoughts of harming yourself or your baby, seek emergency help right away.

**3.What are the hazards of postpartum depression?**

Postpartum depression (PPD) is more than “baby blues.” When it’s untreated or severe, it can create real hazards for the parent, the baby, and the whole family. ([Mayo Clinic][1])

**Hazards for the parent**

Difficulty functioning and self-care:** PPD can cause persistent low mood, loss of interest, severe fatigue, sleep/appetite disruption, guilt/worthlessness, anxiety/irritability, and trouble concentrating—making it hard to manage daily tasks and recovery after birth. ([国家生物技术信息中心][2])

* **Impaired ability to care for the baby:** Symptoms can interfere with feeding routines, attending medical appointments, and responding consistently to the baby’s needs. ([Cleveland Clinic][3])

* **Relationship and social harms:** Depression can strain partner relationships, reduce social support, and increase conflict at home. ([PMC][4])

* **Risk escalation in severe cases:** Some people develop **thoughts of self-harm**; this is an emergency warning sign. ([Mayo Clinic][1])

**Hazards for the baby and child**

* **Bonding and interaction difficulties:** PPD is associated with problems in mother–infant bonding and less sensitive or less responsive interactions, which can ripple into later emotional/behavioral outcomes. ([PMC][4])

* **Health and development impacts:** Public health reviews link perinatal depression with poorer maternal and infant health outcomes overall, and research has found benefits to child development when postnatal depression is effectively treated. ([疾病控制与预防中心][5])

**When to seek help urgently**

If you or someone you know has **thoughts of self-harm, harming the baby, severe confusion, or feels out of control**, seek emergency help immediately. In Japan, call **119** (ambulance) or **110** (police). If you’re in the U.S., you can call/text **988** for the Suicide & Crisis Lifeline.

PPD is treatable—commonly with therapy, medication, or both—so getting help early can reduce risks for both parent and baby. ([Mayo Clinic][6])

[1]:https://www.mayoclinic.org/diseases-conditions/postpartum-depression/symptoms-causes/syc-20376617?utm_source=chatgpt.com "Postpartum depression - Symptoms and causes"

[2]: https://www.ncbi.nlm.nih.gov/books/NBK519070/?utm_source=chatgpt.com "Perinatal Depression - StatPearls - NCBI Bookshelf"

[3]:https://my.clevelandclinic.org/health/diseases/9312-postpartum-depression?utm_source=chatgpt.com "Postpartum Depression: Causes, Symptoms & Treatment"

[4]:https://pmc.ncbi.nlm.nih.gov/articles/PMC6492376/?utm_source=chatgpt.com "Consequences of maternal postpartum depression"

[5]: https://www.cdc.gov/mmwr/volumes/69/wr/mm6919a2.htm?utm_source=chatgpt.com "Vital Signs: Postpartum Depressive Symptoms and ..."

[6]:https://www.mayoclinic.org/diseases-conditions/postpartum-depression/diagnosis-treatment/drc-20376623?utm_source=chatgpt.com "Postpartum depression - Diagnosis and treatment"

**4.When does postpartum depression usually start? How long does postpartum depression last?**

Postpartum depression (PPD) most often begins in the first few weeks after childbirth, but it doesn’t have a single “start date.” Many people notice symptoms emerging **within 1–3 weeks postpartum**, and a large share of cases start **within the first 3 months**. PPD can also begin **during pregnancy** (sometimes called perinatal or antenatal depression) or appear **later in the first year after delivery**—especially as sleep deprivation, feeding challenges, pain, hormonal shifts, or stress accumulate. A key distinction is that “baby blues” are very common in the first days after birth and typically peak around day 4–5; they are usually milder and resolve on their own within about two weeks. If low mood, anxiety, irritability, or hopelessness **lasts beyond two weeks**, gets worse, or interferes with daily functioning or bonding, PPD becomes more likely.

How long PPD lasts varies widely. **With appropriate treatment and support**, many people start to feel meaningful improvement within **weeks**, and substantial recovery often occurs over **several months**. Without treatment, symptoms can persist much longer—**many months** and sometimes **a year or more**—and can become chronic for some. Duration is influenced by factors such as prior depression or anxiety, severity at onset, social support, sleep disruption, relationship stress, medical complications, and access to care.

If you (or someone you care about) are experiencing symptoms, it’s worth reaching out early to an OB-GYN, primary care clinician, midwife, or mental health professional—PPD is common and treatable. **Seek urgent help immediately** if there are thoughts of self-harm, harming the baby, feeling detached from reality, paranoia, or hallucinations (possible postpartum psychosis). If you tell me roughly how many weeks postpartum you’re asking about and what symptoms are showing up, I can help you think through what’s typical and what kind of support to seek.

**5.What are the risk factors for postpartum depression?**

Postpartum depression (PPD) is influenced by a mix of biological, psychological, and social factors. Having one risk factor doesn’t mean someone will develop PPD—but risk rises as factors stack up.

**Strongest and most consistent risk factors**

* **Personal history of depression or anxiety** (including during pregnancy). Prior mood disorders are among the best predictors of PPD.

* **Depression or anxiety during pregnancy** (“antenatal” depression/anxiety). Symptoms before delivery commonly continue or worsen after birth.

* **Previous postpartum depression** after an earlier pregnancy.

**Psychosocial and relationship factors**

* **Low social support** (feeling alone, little practical help, limited emotional support).

* **High stress load**, such as financial strain, housing insecurity, work/immigration stress, or major life events (bereavement, divorce, job loss).

* **Relationship conflict** or intimate partner violence. Feeling unsafe or unsupported at home significantly increases risk.

* **Unplanned or unwanted pregnancy**, or distress about the pregnancy/birth experience.

**Birth- and baby-related factors**

* **Traumatic or complicated delivery**, emergency C-section, severe pain, hemorrhage, ICU admission, or a baby needing NICU care.

* **Feeding difficulties** (e.g., painful breastfeeding, low milk supply concerns) and persistent infant crying can add stress and sleep loss.

* **Preterm birth** or medical complications in the baby.

**Biological and health-related factors**

* **Sleep deprivation** and disrupted sleep (one of the most important modifiable contributors).

* **Hormonal sensitivity** (rapid postpartum shifts in estrogen/progesterone may trigger symptoms in vulnerable individuals).

* **Thyroid problems**, anemia, chronic pain, or other medical issues that mimic or worsen depressive symptoms.

* **Substance use** (alcohol/drugs) can both increase risk and complicate recovery.

**Contextual factors**

* **Young maternal age**, limited access to healthcare, and socioeconomic disadvantage are associated with higher risk, often through stress and reduced support.

If you’d like, tell me whether you want this framed for a patient handout, a school assignment, or a research-style paragraph (and whether you mean “postpartum depression” specifically or “perinatal mood disorders” more broadly).

**6.How do you tell postpartum blues from postpartum depression?**

Postpartum “blues” and postpartum depression can look similar at first (tearfulness, irritability, feeling overwhelmed), but they differ in **timing, intensity, duration, and impact on functioning**.

**Postpartum blues (“baby blues”)**

* **When it starts:** Usually within the first **2–3 days after delivery**.

* **How long it lasts:** Peaks around day 4–5 and typically resolves on its own within **2 weeks**.

* **Symptoms:** Mood swings, crying easily, anxiety, irritability, feeling emotionally “raw,” trouble sleeping (even when the baby sleeps), and feeling overwhelmed—while still having moments of enjoyment and bonding.

* **Functioning:** You may feel fragile, but you can generally still care for yourself and the baby, especially with support.

* **Treatment:** Reassurance, rest, practical help, social support, and monitoring are often enough.

**Postpartum depression (PPD)**

* **When it starts:** Can begin anytime in the first few weeks, often within the **first 1–3 months**, and may also start during pregnancy (perinatal depression).

* **How long it lasts:** **More than 2 weeks**, and doesn’t steadily improve without support.

* **Symptoms:** Persistent sadness or emptiness, loss of interest or pleasure, frequent guilt or worthlessness, feeling disconnected from the baby, hopelessness, significant anxiety or panic, appetite changes, and sleep disruption beyond normal newborn-care patterns. Some people have scary intrusive thoughts (e.g., fears of harm coming to the baby) that feel unwanted and distressing.

* **Functioning:** Symptoms **interfere** with daily life—difficulty caring for the baby or yourself, withdrawing from others, or feeling unable to cope.

* **Treatment:** Often benefits from **therapy** (like CBT or interpersonal therapy), support groups, and sometimes **medication**. Early treatment matters and is very effective.

**Big “seek help now” red flags**

* Thoughts of **self-harm**, suicide, or harming the baby

* Feeling unable to safely care for the baby

* Severe agitation, confusion, hearing voices, or paranoia (possible postpartum psychosis—an emergency)

A practical rule: if symptoms are **getting worse**, last **beyond two weeks**, or **disrupt your ability to function**, it’s more consistent with postpartum depression and worth contacting a healthcare professional promptly.

**7.Does a prior history of depression or anxiety increase PPD risk?**

Yes. A prior personal history of **depression and/or anxiety** is one of the clearest, most consistently reported risk factors for postpartum depression (PPD).

Clinical references that summarize the research routinely list **a personal history of depression and anxiety** among the major psychological risk factors for developing perinatal (pregnancy-to-postpartum) depression. ([国家生物技术信息中心][1]) In patient-facing guidance, the UK NHS similarly notes that you are more likely to experience postnatal depression if you’ve had **mental health problems (especially depression) earlier in life** or **during pregnancy**. ([nhs.uk][2])

Why does this happen? The postpartum period involves rapid hormonal shifts, sleep disruption, and big psychosocial changes. For someone who has previously had depression or anxiety, those stressors can more easily “reactivate” underlying vulnerabilities in mood regulation (for example, stress-response and sleep systems), especially if there are added pressures such as limited support or relationship stress.

It’s also important to distinguish *timing*: symptoms **during pregnancy** strongly predict postpartum symptoms. Research consistently finds that **antenatal depression** is associated with substantially higher odds of PPD, with reported odds ratios in one review ranging from about **1.5 up to 7.5**, depending on the study and how/when PPD was measured. ([Springer][3]) Anxiety in pregnancy is also strongly linked with later PPD; one study found that high antenatal worry predicted postpartum depressive symptoms (odds ratio ~**3.9**), and it also summarizes evidence that antenatal anxiety is associated with PPD across studies. ([PMC][4])

Practical takeaway: if you have a history of depression or anxiety, it doesn’t mean you *will* get PPD—but it’s a signal to plan ahead. Early screening in pregnancy and postpartum, a support plan (sleep protection, help at home), and timely therapy and/or medication when appropriate can meaningfully reduce severity and duration if symptoms emerge. ([国家生物技术信息中心][1])

[1]: https://www.ncbi.nlm.nih.gov/books/NBK519070/ "Perinatal Depression - StatPearls - NCBI Bookshelf"

[2]:https://www.nhs.uk/mental-health/conditions/post-natal-depression/overview/ "Postnatal depression - NHS"

[3]:https://link.springer.com/article/10.1186/s12888-023-05030-1 "Risk factors for the development of postpartum depression in individuals who screened positive for antenatal depression | BMC Psychiatry | Springer Nature Link"

[4]: https://pmc.ncbi.nlm.nih.gov/articles/PMC8442474/ "High Worry in Pregnancy Predicts Postpartum Depression - PMC"

**8.What are early warning signs of postpartum depression?**

Postpartum depression (PPD) is more than the “baby blues.” The baby blues are very common and usually peak around days 3–5 after delivery and improve within about 2 weeks. PPD symptoms tend to be **more intense, last longer than 2 weeks, or start later** (sometimes weeks to months after birth) and can make it hard to function or care for yourself or your baby.

**Early warning signs can include:**

* **Persistent low mood or frequent crying** that doesn’t lift, even when things are going “okay.”

* **Loss of interest or pleasure** in activities you normally enjoy, or feeling emotionally “numb.”

* **Irritability, anger, or feeling overwhelmed** much of the day.

* **Anxiety that feels out of proportion**, constant worry, racing thoughts, or feeling on edge. Some people mainly experience anxiety rather than sadness.

* **Sleep problems beyond typical newborn-related sleep loss**: inability to sleep even when the baby sleeps, or sleeping excessively and still feeling exhausted.

* **Changes in appetite** (not eating, or overeating), low energy, and feeling physically slowed down.

* **Trouble concentrating**, making decisions, or remembering things.

* **Excessive guilt, shame, or feeling like a “bad parent,”** or feeling you’re failing despite trying hard.

* **Difficulty bonding with the baby**, feeling disconnected, or fearing you don’t love your baby “enough.”

* **Withdrawing from others**, avoiding calls/texts, or not wanting help/visitors when you normally would.

* **Intrusive thoughts** (unwanted scary thoughts about harm coming to the baby). These can happen with anxiety or PPD—what matters is how distressing and persistent they are and whether you feel in control.

**Get urgent help right away** (call your local emergency number or go to the ER) if you have thoughts of harming yourself or your baby, feel you might act on scary thoughts, feel confused/disoriented, have hallucinations, or feel extremely “wired,” agitated, and unable to sleep (which can signal postpartum psychosis, a rare emergency).

If you’re noticing several of these signs, it’s a good idea to talk to your OB/GYN, midwife, primary care clinician, or a mental health professional—PPD is treatable, and early support helps a lot.

**9.How can partners/family recognize signs of postpartum depression?**

Partners and family are often the first to notice when “normal” postpartum adjustment has crossed into postpartum depression (PPD). A helpful starting point is the **time course and intensity**. It’s common for many new parents to have “baby blues” in the first 1–2 weeks after birth—tearfulness, irritability, and feeling overwhelmed that comes and goes. **PPD is more likely when symptoms last longer than two weeks, feel worse over time, or interfere with daily functioning and bonding.**

Signs partners/family can watch for include:

* **Persistent low mood or frequent crying** that doesn’t lift, or a sense of numbness/emptiness.

* **Loss of interest or pleasure** in things they usually enjoy, including reduced engagement with the baby or family.

* **Marked irritability, anger, or anxiety**, sometimes presenting more as agitation than sadness.

* **Sleep problems beyond newborn-related sleep loss** (can’t sleep even when the baby sleeps) or sleeping excessively.

* **Appetite changes**, low energy, and difficulty concentrating or making decisions.

* **Excessive guilt, shame, or feelings of being a “bad parent,”** or beliefs that the baby/family would be better off without them.

* **Withdrawal and isolation**, avoiding friends, family, or help.

* **Intrusive, scary thoughts** (e.g., fears of harm coming to the baby) or compulsive checking; these can be part of postpartum anxiety and may overlap with PPD.

Partners can also notice **practical red flags**: missing meals, skipping showers, not leaving bed, being unable to complete simple tasks, or appearing “unreachable” emotionally.

If you suspect PPD, respond with **support, not debate**: “I’m worried because you seem really down and exhausted. You’re not alone—can we talk to your doctor/midwife today?” Offer concrete help (take a night feeding, schedule the appointment, handle chores). **Urgent help is needed immediately** if there are thoughts of self-harm, harming the baby, hearing/seeing things others don’t, extreme confusion, or rapid mood swings. In those cases, contact emergency services or a local crisis line right away.

**10.Can fathers/partners experience postpartum depression too?**

Partners and family are often the first to notice when “normal” postpartum adjustment has crossed into postpartum depression (PPD). A helpful starting point is the **time course and intensity**. It’s common for many new parents to have “baby blues” in the first 1–2 weeks after birth—tearfulness, irritability, and feeling overwhelmed that comes and goes. **PPD is more likely when symptoms last longer than two weeks, feel worse over time, or interfere with daily functioning and bonding.**

Signs partners/family can watch for include:

* **Persistent low mood or frequent crying** that doesn’t lift, or a sense of numbness/emptiness.

* **Loss of interest or pleasure** in things they usually enjoy, including reduced engagement with the baby or family.

* **Marked irritability, anger, or anxiety**, sometimes presenting more as agitation than sadness.

* **Sleep problems beyond newborn-related sleep loss** (can’t sleep even when the baby sleeps) or sleeping excessively.

* **Appetite changes**, low energy, and difficulty concentrating or making decisions.

* **Excessive guilt, shame, or feelings of being a “bad parent,”** or beliefs that the baby/family would be better off without them.

* **Withdrawal and isolation**, avoiding friends, family, or help.

* **Intrusive, scary thoughts** (e.g., fears of harm coming to the baby) or compulsive checking; these can be part of postpartum anxiety and may overlap with PPD.

Partners can also notice **practical red flags**: missing meals, skipping showers, not leaving bed, being unable to complete simple tasks, or appearing “unreachable” emotionally.

If you suspect PPD, respond with **support, not debate**: “I’m worried because you seem really down and exhausted. You’re not alone—can we talk to your doctor/midwife today?” Offer concrete help (take a night feeding, schedule the appointment, handle chores). **Urgent help is needed immediately** if there are thoughts of self-harm, harming the baby, hearing/seeing things others don’t, extreme confusion, or rapid mood swings. In those cases, contact emergency services or a local crisis line right away.

**11.Can postpartum depression affect lactation or breastfeeding success?**

Yes. Postpartum depression (PPD) can affect both lactation physiology and the day-to-day behaviors that make breastfeeding “work,” so it can reduce breastfeeding success for some parents—though many people with PPD are still able to breastfeed with the right support. ([疾病控制与预防中心][1])

**How PPD can interfere with lactation and breastfeeding success**

* **Stress biology can disrupt milk production and let-down.** Depression and psychological distress are associated with higher stress hormones and changes in the hormones involved in lactation (notably oxytocin for milk ejection/let-down and prolactin for milk production). The evidence overall suggests distress can impair lactation and breastfeeding outcomes, even though individual experiences vary. ([PMC][2])

* **Lower energy, motivation, and executive function.** PPD can make it harder to keep up with frequent feeding/pumping, respond to early hunger cues, or persist through common early challenges (pain, latch issues, cluster feeding). ([美国妇产科医师学会][3])

* **Anxiety and reduced confidence.** PPD often comes with worry and low self-efficacy (“I’m not making enough milk,” “I’m doing it wrong”), which can lead to supplementing or stopping earlier than planned. ([SpringerLink][4])

* **Sleep disruption and bonding difficulties.** PPD can affect parent–infant interaction, and breastfeeding is partly a “two-person system”; if either side is struggling (baby fussiness, parent overwhelm), feeds can become more difficult. ([restoredcdc.org][5])

**Also important: the relationship is bidirectional.** Breastfeeding difficulties can increase stress and worsen mood, while improving feeding support can sometimes ease distress. ([PMC][2])

**What helps (practically)**

* Get **both**: mental health care (therapy and/or medication when appropriate) and **lactation support** (IBCLC or a breastfeeding medicine clinic). Treating PPD is part of treating feeding problems. ([美国妇产科医师学会][3])

* If medication is being considered: many antidepressants can be compatible with breastfeeding, and decisions can be individualized with your clinician. ([疾病控制与预防中心][1])

If you tell me whether you’re currently breastfeeding, pumping, combo-feeding, or pregnant and planning, I can tailor tips to your situation.

[1]:https://www.cdc.gov/breastfeeding-special-circumstances/hcp/illnesses-conditions/postpartum-depression.html?utm_source=chatgpt.com"Postpartum Depression | Breastfeeding special ..."

[2]:https://pmc.ncbi.nlm.nih.gov/articles/PMC8960332/?utm_source=chatgpt.com "Maternal psychological distress and lactation and ..."

[3]:https://www.acog.org/womens-health/faqs/postpartum-depression?utm_source=chatgpt.com "Postpartum Depression"

[4]: https://bmcpregnancychildbirth.biomedcentral.com/counter/pdf/10.1186/s12884-024-06465-4.pdf?utm_source=chatgpt.com "Association between postpartum depression and ..."

[5]:https://restoredcdc.org/www.cdc.gov/mmwr/volumes/66/wr/mm6606a1.htm?utm_source=chatgpt.com "Trends in Postpartum Depressive Symptoms — 27 States, ..."

**12.Can postpartum depression affect a baby’s development?**

Yes. Postpartum depression (PPD) can affect a baby’s development—but it’s not “inevitable,” and support and treatment can substantially reduce risks.

Babies develop through a constant back-and-forth with caregivers: eye contact, smiling, soothing, talking, feeding cues, play, and predictable routines. When a parent is experiencing PPD, they may feel withdrawn, numb, anxious, exhausted, or overwhelmed. That can make it harder to respond consistently to the baby’s signals. Over time, this can influence several areas of development:

* **Emotional and social development:** Babies may show more fussiness, difficulty settling, or less positive engagement if they experience fewer warm, responsive interactions. Some studies link ongoing, untreated PPD with higher risk of later anxiety or behavior problems.

* **Language and cognitive development:** Frequent talking, reading, and playful interaction support early learning. If depression reduces these interactions, some children may show delays in language or attention skills—especially when symptoms are severe or long-lasting.

* **Sleep, feeding, and growth:** PPD can disrupt routines. Babies may have more sleep difficulties or feeding challenges, and stress in the household can affect growth indirectly.

* **Stress biology:** Chronic stress in a caregiving environment can influence an infant’s stress response system. This doesn’t mean permanent harm, but it can shape how a child learns to regulate emotions.

The good news: **PPD is treatable**, and many babies do very well when caregivers get help. Protective factors include another supportive adult, stable routines, skin-to-skin contact, and “good-enough” caregiving (not perfection). Treatment options can include therapy (like CBT or interpersonal therapy), support groups, practical help with sleep and meals, and sometimes medication that can be compatible with breastfeeding—decisions best made with a clinician.

If you’re worried about yourself or someone else, it’s worth contacting a healthcare provider. If there are thoughts of self-harm or harming the baby, seek urgent help immediately.

**13.What treatments are available for postpartum depression (therapy/meds/combined)?**

Postpartum depression (PPD) is treatable, and the best option depends on symptom severity, safety considerations (especially if breastfeeding), prior depression history, and how quickly you need relief. In practice, treatments fall into **therapy**, **medications**, and **combined approaches**—with a few additional supports that can meaningfully help.

\### Therapy (often first-line for mild–moderate PPD)

* **Cognitive Behavioral Therapy (CBT):** Focuses on changing unhelpful thoughts (“I’m a bad mom”) and building practical coping skills (sleep strategies, problem-solving, activity scheduling).

* **Interpersonal Therapy (IPT):** Especially well-suited to postpartum issues because it targets role changes, relationship strain, grief/loss (e.g., a difficult birth), and social support.

* **Supportive counseling / peer support groups:** Not a replacement for formal therapy in more severe cases, but can reduce isolation and shame, and provide practical parenting validation.

* **Mother–infant focused approaches:** Some therapists incorporate bonding/attachment work when guilt, anxiety, or emotional “numbness” interferes with connection.

\### Medications (often for moderate–severe PPD or when therapy alone isn’t enough)

* **SSRIs** are commonly used (e.g., sertraline, escitalopram). Many people can take antidepressants while breastfeeding; clinicians choose based on infant exposure data, past response, and side effects.

* **SNRIs** (e.g., venlafaxine, duloxetine) may help when SSRIs haven’t worked or if anxiety/pain symptoms are prominent.

* If there’s **bipolar disorder risk** (history of mania/hypomania), antidepressants alone can worsen symptoms—mood stabilizer planning is important.

**PPD-specific medicines:** In some countries, **brexanolone** (IV infusion) and **zuranolone** (oral) are approved specifically for postpartum depression and can work faster than standard antidepressants. Access, cost, and breastfeeding guidance vary.

\### Combined treatment (often most effective for many people)

Using **therapy + medication** tends to produce better outcomes for moderate–severe depression, recurrent depression, suicidality, or major functional impairment, because meds can reduce biological symptom burden while therapy builds skills and support.

\### Additional supports

* **Sleep protection** (shared night feeds, pumping plan, or family help) can be pivotal.

* **Address medical contributors** (thyroid issues, anemia, pain).

* **Higher levels of care** (intensive outpatient, partial hospitalization, or inpatient) if symptoms are severe, psychosis occurs, or safety is a concern.

If you tell me whether you’re breastfeeding and how severe symptoms feel (mild/moderate/severe), I can outline what a typical stepwise plan looks like. If you’re having thoughts of harming yourself or your baby, seek urgent help immediately (ER/local emergency number or a crisis line).

**14.Will I get PPD with a future pregnancy, and how can I prevent it?**

Postpartum depression (PPD) can happen after any pregnancy, but having it once **does increase the chance** of experiencing it again. That said, it’s not a guarantee—many people with a prior episode do *not* have PPD in later pregnancies, especially when they plan ahead and get support early. Risk is influenced by a mix of biology and life context: a personal or family history of depression/anxiety, severe PMS/PMDD, depression during pregnancy, sleep deprivation, limited support, relationship stress, birth complications, feeding difficulties, financial strain, and major life events can all raise risk.

The most effective “prevention” is really **early identification + early treatment**, often started *during pregnancy* (not after delivery).

**What you can do now (practical prevention plan):**

* **Tell your OB/midwife early** (first prenatal visit) about any past depression, anxiety, trauma, or PPD. Ask for a prevention plan and routine screening in pregnancy and postpartum.

* **Start therapy in pregnancy** if you have risk factors. CBT and interpersonal therapy have strong evidence for preventing or reducing perinatal depression, especially when begun before symptoms escalate.

* **Build a postpartum support map:** who will do nights, meals, errands, and baby care. Lack of sleep is a major trigger. Plan for protected sleep blocks (even 4–5 uninterrupted hours can help).

* **Make a “watch list” of your early warning signs** (e.g., irritability, crying, dread at bedtime, intrusive guilt, feeling detached). Share it with your partner/support person so they can notice changes too.

* **Consider medication planning** if you have had moderate–severe depression/PPD or multiple episodes. For some people, continuing an antidepressant during pregnancy or starting immediately postpartum meaningfully lowers relapse risk. This is an individualized decision with your clinician, balancing benefits and pregnancy/lactation considerations.

* **Schedule early postpartum check-ins:** not just the routine 6-week visit—aim for 1–2 weeks postpartum and again at 4–6 weeks.

**Get urgent help immediately** if you have thoughts of harming yourself or your baby, feel out of touch with reality, or have severe agitation/insomnia—those can signal a psychiatric emergency (including postpartum psychosis).

If you tell me whether you’ve had PPD before, and how severe it was (and whether you’re currently pregnant), I can help you sketch a prevention plan you can bring to your clinician.

**15.Do exercise, sunlight, diet, and social support help with PPD?**

Yes—those factors can help **reduce symptoms and support recovery** from postpartum depression (PPD), but they’re best thought of as **adjuncts** to evidence-based care (therapy and/or medication) when PPD is moderate–severe or not improving. PPD is a **treatable medical condition**, and getting help early matters. ([美国妇产科医师学会][1])

\### Exercise

Regular physical activity has fairly consistent evidence for improving postpartum depressive symptoms, with benefits depending on the program type, intensity, and how supported/realistic it is for new parents. Systematic reviews and meta-analyses of exercise interventions in the postpartum period generally find **small-to-moderate symptom improvement**. ([PubMed][2])

Practical takeaway: gentle, doable movement (walking, postpartum-safe strengthening) is often a good starting point—especially if it also gets you out of the house.

\### Sunlight / light exposure

“Sunlight” specifically is hard to study, but **bright light therapy** (structured, high-lux morning light) has been investigated for **perinatal depression**, with reviews suggesting it *may* help some people and is generally well tolerated, though evidence is not as extensive as for psychotherapy/meds. ([PMC][3])

Practical takeaway: morning outdoor light + consistent sleep/wake routines may support circadian rhythm; for persistent symptoms, discuss whether light therapy is appropriate.

\### Diet

Diet quality is associated with depression risk in general, and some perinatal studies suggest links between dietary patterns and PPD symptoms—though results can be mixed and not always causal. ([PMC][4])

Omega-3s (especially higher-EPA formulations) have shown **possible benefit** in some meta-analyses for perinatal depressive symptoms, but other reviews conclude evidence is **insufficient or inconsistent**, so it’s not a guaranteed treatment. ([Nature][5])

\### Social support

Strong social support is a major protective factor, and structured interventions like **peer support** have evidence for reducing postpartum depressive symptoms in some studies. ([PMC][6])

If you (or someone you care about) might have PPD—especially with thoughts of self-harm or harming the baby—seek urgent help right away (local emergency services or a crisis line), and contact an OB-GYN/primary care clinician for evaluation and treatment options. ([美国妇产科医师学会][1])

[1]:https://www.acog.org/womens-health/faqs/postpartum-depression?utm_source=chatgpt.com "Postpartum Depression - ACOG"

[2]:https://pubmed.ncbi.nlm.nih.gov/29882074/?utm_source=chatgpt.com"Theeffectiveness of exercise-based interventions for ..."

[3]:https://pmc.ncbi.nlm.nih.gov/articles/PMC3518242/?utm_source=chatgpt.com "Efficacy of light therapy for perinatal depression: a review - PMC"

[4]:https://pmc.ncbi.nlm.nih.gov/articles/PMC10947382/?utm_source=chatgpt.com "Antenatal diet quality and perinatal depression"

[5]: https://www.nature.com/articles/s41398-020-00886-3?utm_source=chatgpt.com "The efficacy and safety of omega-3 fatty acids on ..."

[6]:https://pmc.ncbi.nlm.nih.gov/articles/PMC4134152/?utm_source=chatgpt.com"The Effect of Peers Support on Postpartum Depression - PMC"

**16.What can family do most effectively to prevent and treat postpartum depression?**

Family support can make a big difference in both **preventing** postpartum depression (PPD) and **helping recovery** if it occurs. The most effective actions are practical, consistent, and centered on reducing stress while increasing safety and connection.

**1) Learn the signs and take them seriously.**

PPD is more than “baby blues.” Watch for persistent sadness, irritability, anxiety or panic, guilt, hopelessness, loss of interest, sleep disruption beyond baby-related sleep loss, appetite changes, trouble bonding, or scary intrusive thoughts. If these last **more than 2 weeks**, get help promptly. Family can be the “early warning system” when the parent is too exhausted or ashamed to speak up.

**2) Protect sleep—this is often the highest-impact help.**

Sleep deprivation strongly worsens mood. Families can schedule a nightly “protected sleep block” (for example 4–6 uninterrupted hours) by taking over feeding (pumped milk/formula), diapering, and soothing. Even a few nights of improved sleep can noticeably reduce symptoms.

**3) Do concrete tasks, not just encouragement.**

Instead of “Let me know if you need anything,” offer specifics: cook, clean, do laundry, manage older children, handle errands, coordinate visitors, and set up meal trains. Lowering daily burdens reduces overwhelm and supports healing.

**4) Provide emotional support without judgment.**

Listen, validate, and avoid minimizing (“You should be happy”). Use simple language: “I’m here. You’re not failing. This is common and treatable.” Ask what feels hardest today and what would help in the next hour.

**5) Support professional care and follow-through.**

Help arrange appointments, childcare, transportation, and insurance paperwork. Evidence-based treatments include therapy (like CBT or IPT) and, when appropriate, medication. Family support improves the odds of sticking with care.

**6) Build safety plans and know emergency signs.**

Seek urgent help if there are thoughts of self-harm, harming the baby, hallucinations, paranoia, or severe agitation (possible postpartum psychosis). In emergencies, call local emergency services or go to the ER.

**7) Reduce isolation and increase gentle connection.**

Short walks together, brief visits with trusted friends, and parent support groups can help—only if the parent finds them soothing, not draining.

The most effective family approach is: **protect sleep, reduce workload, validate feelings, and actively connect the parent to professional help—quickly.**

**17.When should a patient with postpartum depression seek professional help?**

A patient with postpartum depression (PPD) should seek professional help as soon as symptoms begin to interfere with daily life, bonding with the baby, or personal safety. While “baby blues” are common in the first 1–2 weeks after delivery and usually improve on their own, PPD symptoms are more intense, last longer than two weeks, or worsen over time. Early treatment matters because PPD is highly treatable, and prompt support can reduce suffering and protect both parent and infant well-being.

Professional help is especially important if the patient notices persistent sadness, emptiness, irritability, frequent crying, or a sense of hopelessness; loss of interest in activities; severe anxiety or panic; or overwhelming guilt and feelings of worthlessness. Difficulty sleeping even when the baby sleeps, major appetite changes, fatigue that feels disabling, or trouble concentrating can also signal that support is needed. Patients should reach out if they feel emotionally numb, detached from the baby, or unable to experience pleasure, or if they find themselves avoiding caregiving tasks because they feel incapable or frightened.

Immediate, urgent help is needed if there are thoughts of self-harm, suicide, harming the baby, or if the patient experiences hallucinations, paranoia, severe confusion, or extreme agitation—symptoms that may indicate postpartum psychosis, a medical emergency. In these cases, contacting emergency services or going to the nearest emergency department is appropriate, and a trusted person should stay with the patient.

Even without crisis symptoms, a patient should seek help if they feel they’re “not themselves,” if symptoms persist beyond two weeks postpartum, or if functioning at home, work, or in relationships is deteriorating. Starting with an obstetrician, primary care clinician, or mental health professional is common; many also benefit from therapy, support groups, medication, and practical supports like sleep planning and help with feeding/care routines. If the patient is unsure, it’s still worth reaching out—getting assessed early is safer than waiting.

**18.Can postpartum depression go away without treatment?**

Yes—postpartum depression (PPD) *can* improve and even go away without formal treatment for some people, but it’s unpredictable, can last a long time, and can be risky to “wait it out.”

PPD isn’t the same as the “baby blues.” Baby blues are very common, usually start a few days after delivery, and tend to resolve on their own within about 2 weeks. PPD is more intense (persistent sadness, numbness, irritability, anxiety or panic, sleep/appetite changes beyond what’s expected with a newborn, guilt or feeling like a “bad parent,” difficulty bonding, trouble functioning). Without treatment, PPD may gradually lessen as hormones stabilize, sleep improves, and support increases—but it can also persist for months, recur, or worsen. Untreated PPD is associated with greater strain on relationships, difficulties in day-to-day functioning, and can affect infant bonding and development.

If symptoms are mild and you have strong support, some people recover using “informal” help: more sleep protection, practical support with feeding/chores, regular nutrition, gentle movement, sunlight, and talking openly with trusted people. Still, it’s worth looping in a healthcare professional early, because treatment doesn’t have to mean medication—therapy, support groups, and structured check-ins can be very effective and low-risk.

Get urgent help now if there are thoughts of self-harm, harming the baby, feeling out of control, hallucinations/paranoia, or rapidly worsening mood—these can signal emergencies (including postpartum psychosis) and need immediate care.

If you tell me how long it’s been since delivery and what symptoms you’re noticing, I can help you gauge whether it sounds more like baby blues, PPD, or something that needs urgent attention.

**19.Can postpartum depression relapse after stopping meds, and what’s the recurrence risk?**

Yes. Postpartum depression (PPD) can **return (relapse)** after you stop antidepressants—especially if medication is stopped **too early**, **too quickly**, or if the underlying depression is **recurrent/severe**. It’s also easy to confuse **antidepressant withdrawal/discontinuation symptoms** (e.g., dizziness, “brain zaps,” anxiety, insomnia, flu-like feelings) with relapse, which is one reason clinicians usually recommend a **gradual taper** and close follow-up.

How big is the relapse/recurrence risk?

It depends on *what you mean by recurrence*:

**1) Relapse after stopping meds (same episode coming back).**

Postpartum-specific relapse numbers vary by study, but in depression generally, relapse after discontinuation is common—one large population-based analysis found **about 1 in 3 people relapsed within a year of stopping antidepressants**, and shorter treatment duration was linked with higher relapse risk (with findings suggesting **~10–12 months** of treatment may be preferable when relapse is a concern). ([PMC][1]) Clinical guidance for major depression also describes a **continuation phase of ~4–9 months after remission** to reduce relapse risk, with longer maintenance for people at higher risk. ([AAFP][2])

**2) Recurrence in a later pregnancy/postpartum period (a new postpartum episode).**

A large Danish registry study (women with no prior psychiatric history before their first birth) found that among those who had a postpartum affective episode after the first birth, **about 15%** (if treated with postpartum antidepressants) and **about 21%** (if severe enough to involve hospital contact) had a **recurrent postpartum episode after a second birth**. ([PLOS][3]) Risk can be higher in people with a longer or more recurrent depression history than this “first-episode” cohort.

\### What raises your risk?

Common risk boosters include: prior depressive episodes (especially multiple), severe symptoms, comorbid anxiety, bipolar disorder history (important because treatment differs), major sleep deprivation, poor support, and high stress. PPD can also be longer-lasting than many expect—ACOG notes **about one quarter** of people with perinatal depression may have symptoms for **up to 3 years postpartum**. ([Project Teach NY][4])

\### Practical takeaways

* Don’t stop abruptly—**taper with your prescriber** and set a monitoring plan (often weekly/biweekly check-ins early on).

* Have a relapse plan: therapy/CBT, sleep protection, partner/family support, and clear “early warning signs.”

* Seek urgent help immediately for suicidal thoughts, thoughts of harming the baby, or symptoms of postpartum psychosis.

If you tell me **how long you’ve been well**, **how long you’ve been on meds**, and whether you’ve had **depression before pregnancy**, I can help you think through what “higher vs lower” recurrence risk looks like in your situation.

[1]:https://pmc.ncbi.nlm.nih.gov/articles/PMC8739188/?utm_source=chatgpt.com "Discontinuation of antidepressants: Is there a minimum time ..."

[2]:https://www.aafp.org/pubs/afp/issues/2023/0200/pharmacologic-treatment-of-depression.html?utm_source=chatgpt.com "Pharmacologic Treatment of Depression"

[3]:https://journals.plos.org/plosmedicine/article?id=10.1371%2Fjournal.pmed.1002392 "Risk, treatment duration, and recurrence risk of postpartum affective disorder in women with no prior psychiatric history: A population-based cohort study | PLOS Medicine"

[4]:https://projectteachny.org/app/uploads/2024/04/ACOG-clin-guidelines-treatment_and_management_of_mental_health.2023.pdf "ONG-23-580 1262..1288"

**20.How do you differentiate postpartum depression from postpartum PTSD—and can they co-occur?**

Postpartum depression (PPD) and postpartum PTSD can look similar on the surface (sleep problems, irritability, anxiety, “not feeling like myself”), but they’re driven by different core symptom patterns—and yes, they can absolutely co-occur.

\### How postpartum depression typically presents

PPD is essentially a **major depressive episode** happening in the perinatal period. The *center of gravity* is **persistent low mood and/or loss of interest/pleasure** (anhedonia), plus symptoms like guilt/worthlessness, appetite and sleep changes, low energy, poor concentration, and sometimes thoughts of self-harm. Clinically, “peripartum onset” in DSM terminology is often anchored to onset during pregnancy or within the first weeks after delivery, though in practice many clinicians and guidelines consider depressive episodes across the broader postpartum year. ([国家生物技术信息中心][1])

How postpartum PTSD typically presents

Postpartum PTSD is anchored to a **traumatic stress response** related to childbirth or perinatal events (e.g., feeling intense fear/helplessness during delivery, emergency procedures, severe complications, neonatal ICU events). The hallmark is **trauma-pattern symptoms**, typically clustered as:

* **Intrusions/re-experiencing** (flashbacks, nightmares, distress when reminded)

* **Avoidance** (avoiding hospital reminders, birth conversations, medical settings)

* **Negative changes in mood/cognition** (persistent fear, shame, self-blame, emotional numbing)

* **Hyperarousal** (startle, hypervigilance, irritability, sleep disruption) ([DIGIBUG][2])

A practical clue: if distress **spikes with trauma reminders** (baby’s cry, OB visits, birth story, hospital smells/sounds), PTSD moves up the list.

\### Can they co-occur?

Yes—commonly. Research on childbirth-related PTSD notes substantial overlap with postpartum depressive symptoms, and studies specifically examining comorbidity find that many people who screen positive for postpartum PTSD also report significant depressive symptoms. ([PMC][3])

\### What to do clinically

Because symptoms overlap, many professional bodies encourage **routine screening** for perinatal mental health concerns and follow-up assessment when screens are positive. ([美国妇产科医师学会][4]) If you’re asking for yourself or someone close to you and there are any thoughts of self-harm, it’s important to seek urgent, real-time help from local emergency services or a crisis line.

[1]: https://www.ncbi.nlm.nih.gov/books/NBK519070/?utm_source=chatgpt.com "Perinatal Depression - StatPearls - NCBI Bookshelf"

[2]:https://digibug.ugr.es/bitstream/10481/71014/1/ACCEPTED%20VERSION.pdf?utm_source=chatgpt.com "Abstract Postpartum post-traumatic stress disorder (PP- ..."

[3]:https://pmc.ncbi.nlm.nih.gov/articles/PMC7182486/?utm_source=chatgpt.com "Beyond Postpartum Depression: Posttraumatic Stress ..."

[4]:https://www.acog.org/programs/perinatal-mental-health/patient-screening?utm_source=chatgpt.com "Patient Screening"

DeepSeek

**1.What is postpartum depression?**

Postpartum depression (PPD) is a serious and complex mood disorder that affects individuals after childbirth, characterized by persistent feelings of extreme sadness, anxiety, and exhaustion that interfere with a mother's ability to care for herself and her family. It is a clinical depression, distinct from the more common and short-lived "baby blues," which affect up to 80% of new mothers and typically resolve within two weeks. PPD, however, is more severe, longer-lasting, and requires medical attention.

Symptoms usually develop within the first few weeks after delivery but can begin up to a year postpartum. They encompass emotional, physical, and behavioral changes. Key symptoms include a persistently depressed mood or severe mood swings, excessive crying, intense irritability or anger, and difficulty bonding with the baby. Mothers with PPD often experience overwhelming fatigue yet may have insomnia, even when the baby is sleeping. They may withdraw from family and friends, lose interest in activities they once enjoyed, and suffer from intense anxiety or panic attacks. Feelings of worthlessness, shame, guilt, or inadequacy—particularly about not being a "good mother"—are common. In severe cases, there may be thoughts of harming oneself or the baby, which constitutes a medical emergency.

The causes of PPD are not attributed to a single factor but are understood to be a combination of physical, emotional, and lifestyle influences. The dramatic hormonal shifts following delivery, particularly drops in estrogen and progesterone, are significant physical triggers. Emotional factors include the stress of new responsibilities, sleep deprivation, feelings of lost identity, and worries about parenting ability. Lifestyle stressors such as a lack of support, financial pressures, and a difficult birth experience also contribute. Additionally, a personal or family history of depression or anxiety increases vulnerability.

Critically, PPD is not a character flaw or a weakness; it is a medical condition with effective treatments. The first step is seeking help from a healthcare provider. Treatment typically involves psychotherapy (talk therapy, such as cognitive-behavioral therapy) and, in many cases, antidepressant medications that are safe for breastfeeding. Support groups, partner and family support, and self-care strategies like rest, nutrition, and moderate exercise are vital components of recovery.

Left untreated, postpartum depression can last for months or longer and can have profound negative effects on the mother’s health, the infant’s development, and the entire family dynamic. With proper diagnosis and treatment, however, the vast majority of women make a full recovery, allowing them to fully engage in the joys of motherhood and family life. Recognizing the signs and seeking help without stigma is paramount.

**2.What are the typical symptoms of postpartum depression?**

**Typical Symptoms of Postpartum Depression**

Postpartum depression (PPD) is a serious mood disorder that can affect individuals after childbirth, characterized by a persistent and intense combination of emotional, physical, and behavioral symptoms. It is crucial to distinguish PPD from the more common and transient "baby blues," as PPD symptoms are more severe, last longer (typically beginning within the first few weeks after delivery but can start up to a year postpartum), and significantly interfere with daily functioning and caregiving.

The emotional and psychological symptoms are often the most prominent. These include a **persistent feeling of sadness, emptiness, or hopelessness** that lasts most of the day, nearly every day. Individuals may experience **severe mood swings**, ranging from irritability and intense anger to overwhelming despair. A marked **loss of interest or pleasure in activities once enjoyed**, including bonding with the baby, is a key symptom (anhedonia). Feelings of **worthlessness, guilt, or shame** are common, often with excessive self-criticism about not being a "good enough" parent. **Anxiety** is frequently intertwined with depression, manifesting as constant worry, racing thoughts, or even panic attacks, often centered on the baby's health or one's own inadequacy. In severe cases, there may be **thoughts of death, suicide, or harming oneself or the baby**, which require immediate medical attention.

Behavioral changes are clear indicators. This can include **withdrawing from family and friends**, avoiding social contact. There may be **difficulty bonding or forming an emotional attachment with the newborn**, which often exacerbates feelings of guilt. Changes in **sleep patterns** are paradoxical; despite extreme fatigue, individuals may suffer from insomnia or, conversely, sleep excessively. **Appetite disturbances**—either eating too little or too much—are common. There may be a noticeable **loss of energy or debilitating fatigue** that makes even small tasks feel impossible, and **restlessness or slowed movements and speech**.

Cognitive symptoms involve a **diminished ability to think clearly, concentrate, or make decisions**. This "brain fog" can make following conversations or remembering tasks very challenging. Physical symptoms are also prevalent and are not merely "in the head." These include persistent **headaches, stomachaches, muscle pains**, and general **aches and pains without a clear cause**.

A critical and less discussed symptom is **intense irritability and rage**, which can be directed at partners, other children, or the baby. Furthermore, some individuals experience **intrusive, frightening thoughts** about accidental harm coming to the infant, which are distressing and not acted upon but contribute significantly to anxiety.

In summary, postpartum depression presents a complex constellation of symptoms that extend far beyond simple sadness. It is a whole-body illness affecting emotional regulation, physical well-being, cognitive function, and behavior. Recognizing these signs is the first step toward seeking help, as PPD is highly treatable with professional intervention such as psychotherapy, support groups, and, when appropriate, medication.

**3.What are the hazards of postpartum depression?**

Postpartum depression (PPD) is a serious mental health condition that extends far beyond the "baby blues," posing significant hazards to the mother, the infant, the partner, and the entire family system. Its dangers are multifaceted, impacting emotional, physical, and social well-being.

For the mother, the most immediate hazard is profound suffering. PPD is characterized by persistent sadness, anxiety, hopelessness, and anhedonia (loss of pleasure), which can severely impair her ability to function daily. This emotional anguish often manifests physically as extreme fatigue, changes in appetite, and psychosomatic pains. Crucially, PPD erodes the maternal-infant bond, a critical component of early development. A mother may feel detached, resentful, or numb toward her baby, leading to inconsistent or minimal interaction. This can disrupt the secure attachment necessary for the child’s emotional security. In severe cases, PPD escalates into suicidal ideation or, more rarely, postpartum psychosis—a medical emergency characterized by delusions or hallucinations that may involve harming oneself or the infant.

The infant’s development is directly jeopardized. Research indicates that children of mothers with untreated PPD are at higher risk for cognitive, emotional, and behavioral difficulties. The lack of responsive, nurturing interaction can affect language acquisition, social skills, and emotional regulation. These children may exhibit more crying, sleep problems, and later, higher rates of anxiety, depression, and attentional issues. The foundational sense of security and trust is compromised, with potential long-term consequences.

The hazards ripple through the family. Partners often experience stress, confusion, and helplessness, which can strain the relationship and increase their own risk of depression. Siblings may feel neglected or anxious in the tense household atmosphere. The family’s overall dynamic becomes disrupted, potentially affecting parenting cohesion and the home environment.

Furthermore, PPD can lead to the neglect of maternal self-care and infant care routines, including adherence to pediatric appointments and feeding schedules. The mother may withdraw from social support networks precisely when she needs them most, leading to isolation and exacerbating the illness.

In conclusion, the hazards of postpartum depression are systemic and severe. It is not a personal failing but a medical condition that, untreated, can alter developmental trajectories, damage relationships, and endanger lives. Recognizing these extensive risks underscores the critical importance of screening, early intervention, and comprehensive treatment—including therapy, social support, and sometimes medication—to ensure the health and safety of the entire family unit.

**4.When does postpartum depression usually start? How long does postpartum depression last?**

Postpartum depression (PPD) is a complex and serious mood disorder that affects many individuals after childbirth. Its onset and duration are not uniform, but follow general patterns that are crucial for recognition and treatment.

**Onset: When Does Postpartum Depression Usually Start?**

Postpartum depression typically begins within the first **1 to 3 weeks after delivery**, making it distinct from the common "baby blues," which emerge within the first 2-3 days postpartum and usually resolve within two weeks. However, it is essential to understand that PPD can start much later. The Diagnostic and Statistical Manual of Mental Disorders (DSM-5) specifies that symptoms must begin within **four weeks after childbirth** for a diagnosis of "postpartum onset." Yet, in clinical practice and broader understanding, many healthcare professionals recognize that PPD can manifest anytime **within the first year** after giving birth. This delayed onset can sometimes be linked to transitions such as returning to work, weaning from breastfeeding, or when social support diminishes. Therefore, while the highest risk period is in the initial weeks, vigilance for symptoms should be maintained throughout the first year.

**Duration: How Long Does Postpartum Depression Last?**

The duration of postpartum depression is highly variable and heavily dependent on several factors, including the severity of symptoms, the presence of a support system, and most critically, whether professional treatment is sought. Without any intervention, PPD can persist for **several months to a year or more**. In some cases, if left untreated, it can develop into a chronic depressive condition.

With appropriate treatment—which often includes psychotherapy (such as cognitive-behavioral therapy or interpersonal therapy), medication (like antidepressants), support groups, and lifestyle adjustments—significant improvement can often be seen within **3 to 6 months**. Many individuals experience a noticeable reduction in symptoms within weeks of starting treatment, but a full course of treatment is typically recommended for at least 6-12 months to prevent relapse. It's important to note that recovery is not always linear; there may be periods of improvement followed by difficult days. Complete remission is the goal, but the journey can take time.

**Key Factors Influencing Duration:**

1. **Timeliness of Intervention:** Early diagnosis and treatment are the strongest predictors of a shorter duration. The longer PPD goes untreated, the more entrenched it can become.

2. **Severity:** Mild cases may resolve more quickly with support and therapy, while moderate to severe episodes often require more comprehensive, longer-term treatment plans.

3. **Support System:** A strong, understanding network of partners, family, and friends can alleviate isolation and practical burdens, aiding recovery.

4. **History of Mental Illness:** Individuals with a prior history of depression or anxiety may have a longer or more complex recovery path.

5. **Additional Stressors:** Financial stress, marital problems, or a medically fragile infant can prolong the episode.

It is also vital to distinguish PPD from the rarer but more severe **postpartum psychosis**, which has a sudden onset within the first 2-3 weeks postpartum and constitutes a medical emergency requiring immediate intervention.

In conclusion, postpartum depression most commonly begins in the first few weeks after childbirth but can appear up to a year later. Its duration, while often measured in months, is not predetermined and can be significantly shortened with professional help. No parent should suffer in silence or feel shame; postpartum depression is a medical condition, not a failure of character or parenting. Seeking help from a healthcare provider—an obstetrician, midwife, or mental health specialist—is the most critical step toward recovery, ensuring both the parent's well-being and the healthy development of the child.

**5.What are the risk factors for postpartum depression?**

Postpartum depression (PPD) is a complex and multifactorial condition, with risk factors spanning biological, psychological, and social domains. Understanding these risks is crucial for early identification, intervention, and support.

The most significant predictive factor is a **personal or family history of depression or anxiety**. A woman with a prior episode of depression, particularly during pregnancy, is at substantially higher risk. Similarly, a family history of mood disorders suggests a genetic vulnerability. **Hormonal fluctuations** play a key biological role; after delivery, the rapid drop in estrogen and progesterone can disrupt neurotransmitter systems, particularly in those already biologically sensitive to such shifts.

Psychological and personality factors are strongly implicated. **High levels of prenatal anxiety** and **perfectionistic or self-critical personality traits** increase vulnerability. Individuals with a history of **premenstrual dysphoric disorder (PMDD)** may also be more sensitive to hormonal changes. A **difficult or traumatic birth experience**, including unplanned C-sections, emergency interventions, or feelings of loss of control, is a notable risk factor.

Crucially, psychosocial and environmental stressors are often the precipitating elements. **Inadequate social support**—whether from a partner, family, or community—is one of the most consistent predictors. This is compounded by **marital or relationship dissatisfaction** and **financial strain**. **Life stressors** coinciding with the postpartum period, such as bereavement, job loss, or housing instability, significantly elevate risk. **Infant-related factors** also contribute, including **premature birth, infant illness or disability, and difficult infant temperament** (e.g., excessive crying, feeding problems), which can exacerbate parental stress and exhaustion.

Additional demographic and situational factors include **young maternal age, low socioeconomic status, and unintended pregnancy**. **Sleep deprivation**, which is nearly universal with a newborn, becomes a severe risk when chronic and severe, eroding emotional resilience. **Breastfeeding difficulties**, often intertwined with pain, guilt, and feelings of inadequacy, can also be a significant stressor.

It is vital to recognize that PPD is not caused by a single factor but by the **cumulative interaction** of these vulnerabilities and stressors. A person with several minor risks may develop PPD, while another with a major biological risk but a supremely supportive environment may not. Furthermore, risk factors do not imply causation or fault; they are indicators for heightened vigilance.

Ultimately, identifying these risks allows healthcare providers to screen proactively and offer targeted support. It also empowers expecting and new parents to build robust support systems, manage expectations, and seek help without shame at the earliest signs of struggle. Postpartum depression is a treatable medical condition, and awareness of its risk factors is a critical step toward ensuring better outcomes for parents and infants alike.

**6.How do you tell postpartum blues from postpartum depression?**

Distinguishing between postpartum blues and postpartum depression is crucial for the well-being of a new parent, as they differ significantly in severity, duration, and required intervention. While both occur after childbirth and share some emotional symptoms, understanding the key differences can guide appropriate support and treatment.

**Postpartum "Baby Blues"** is a very common, transient condition, affecting an estimated 50-80% of new mothers. It is not considered a mental illness but rather a normal adjustment period driven by the dramatic hormonal shifts (especially in estrogen and progesterone), physical exhaustion, and the massive life change that accompanies a new baby.

*	**Symptoms:** These are typically mild and include mood swings, tearfulness, irritability, anxiety, feeling overwhelmed, and sadness. A mother with the blues might cry easily but can also experience moments of joy.

*	**Timeline:** Symptoms usually begin within the first 2-3 days after delivery, peak around days 4-5, and **typically subside on their own within two weeks.**

*	**Functional Impact:** Crucially, the blues do not severely interfere with a mother's ability to care for her baby or function in daily life. She may feel shaky and emotional, but she can still experience pleasure and bond with her infant.

**Postpartum Depression (PPD)**, in contrast, is a clinically diagnosed mood disorder and a major form of depression. It is more severe, persistent, and debilitating, affecting approximately 1 in 7 mothers.

*	**Symptoms:** PPD includes the symptoms of the blues but at a more intense and persistent level. Key indicators are a pervasive sense of sadness, hopelessness, worthlessness, or guilt. Intense irritability or anger is common. A hallmark is a **loss of interest or pleasure in activities once enjoyed, including the baby** (anhedonia). Other severe symptoms can include severe anxiety or panic attacks, difficulty bonding with the baby, intrusive thoughts (which can be about harming oneself or the baby), and significant changes in appetite or sleep unrelated to the baby's needs.

*	**Timeline:** It can begin anytime within the first year postpartum, often emerging within the first few weeks. Crucially, **symptoms last for at least two weeks and often much longer without treatment.**

*	**Functional Impact:** PPD significantly impairs a mother's ability to function. She may struggle to get out of bed, care for herself or her baby, perform daily tasks, or maintain relationships. The feelings are not fleeting but are constant and overwhelming.

**The Core Differences Summarized:**

The blues are **mild, short-lived, and non-disabling**. PPD is **severe, persistent, and disabling**. The most critical distinguishing factors are: 1) **Duration** (blues resolve within two weeks; PPD persists), 2) **Intensity** (PPD involves deeper despair and anhedonia), and 3) **Impact on Functioning** (PPD seriously compromises daily life and caregiving).

**When to Seek Help:**

Any depressive symptoms that are severe, last longer than two weeks, or include thoughts of harming oneself or the baby require **immediate professional attention**. PPD is not a sign of weakness or poor character; it is a medical condition with effective treatments, including therapy and medication. Consulting a healthcare provider—an OB/GYN, midwife, or mental health professional—is essential for diagnosis and a path to recovery. Early intervention is key for the health of both the parent and the child.

**7.Does a prior history of depression or anxiety increase PPD risk?**

Yes, a prior history of depression or anxiety is one of the most significant and well-established risk factors for developing Postpartum Depression (PPD). Extensive research consistently demonstrates that a woman with a history of mood or anxiety disorders is at a substantially elevated risk, with estimates suggesting she is three to five times more likely to experience PPD compared to a woman without such a history. This relationship is complex, involving biological, psychological, and social pathways.

**Prior Depression as a Primary Predictor**

A history of major depressive disorder prior to or during pregnancy is considered the single strongest predictor of PPD. This is often explained through the **"kindling hypothesis"** or **sensitization model**, which posits that each depressive episode lowers the threshold for future episodes, making the brain's stress-response systems more reactive. The monumental physiological, hormonal (particularly the rapid withdrawal of estrogen and progesterone postpartum), and social changes of the perinatal period act as a potent trigger for those with a pre-existing vulnerability. The depression may not be new in content, but the postpartum context shapes its expression, often intertwining with feelings of guilt, inadequacy, or fear about motherhood.

**Prior Anxiety Disorders and Their Specific Role**

A history of anxiety disorders—including Generalized Anxiety Disorder (GAD), Panic Disorder, Social Anxiety, and especially **Obsessive-Compulsive Disorder (OCD)**—is equally significant. Anxiety and depression are frequently comorbid, sharing underlying neurobiological vulnerabilities related to neurotransmitter systems (like serotonin) and hypothalamic-pituitary-adrenal (HPA) axis dysregulation. For some women, pre-existing anxiety may morph into PPD, as relentless worries about the baby's health, one's competence as a mother, or catastrophic thoughts become overwhelming and draining, leading to depressive symptomatology. Importantly, intrusive, distressing thoughts (common in postpartum OCD) are a often-misunderstood symptom that can be severe and are more likely in those with an anxiety predisposition.

**Shared Underlying Mechanisms**

The link is not merely about past diagnosis, but often reflects enduring vulnerabilities:

1. **Biological Sensitivity:** Genetic predispositions and a dysregulated stress response system (HPA axis) may underlie both prior disorders and susceptibility to PPD.

2. **Cognitive and Emotional Styles:** Pre-existing tendencies toward negative cognitive patterns (e.g., rumination, self-criticism, catastrophic thinking) and emotional dysregulation can be powerfully reactivated by the stressors of new motherhood.

3. **Psychosocial Continuity:** Many risk factors for depression and anxiety in general—such as a history of trauma, lack of social support, or chronic stress—persist into the postpartum period, amplifying its challenges.

**Crucial Nuances and Implications**

It is vital to note that a prior history is a **risk factor, not a destiny**. Many women with anxiety or depression histories navigate the postpartum period without developing clinical PPD. However, this known association has critical clinical implications:

*	**Screening and Prevention:** It identifies a population for targeted, proactive care. Enhanced screening during pregnancy and postpartum is essential.

*	**Preconception Counseling:** Women with these histories can benefit from planning, including discussions about medication management, strengthening support systems, and developing relapse prevention plans with mental health professionals.

*	**Differentiated Diagnosis:** It helps clinicians distinguish between a recurrence of a pre-existing condition, a new episode of PPD, or postpartum adjustment difficulties.

In conclusion, a prior history of depression or anxiety substantially increases the risk of PPD by highlighting a pre-existing psychological and biological vulnerability that is often activated by the unique demands of the perinatal period. Recognizing this link is not about stigmatizing mothers but about empowering them and healthcare providers to implement vigilant monitoring, supportive interventions, and timely treatment, thereby safeguarding maternal and infant well-being.

**8.What are early warning signs of postpartum depression?**

Postpartum depression (PPD) is a serious mood disorder that affects many individuals after childbirth, distinct from the more common and short-lived "baby blues." Recognizing the early warning signs is crucial for timely intervention and support. These signs typically emerge within the first few weeks after delivery, but can appear anytime within the first year. They often persist for longer than two weeks and intensify, interfering with daily care and bonding.

**Emotional and Psychological Signs:** One of the earliest and most telling signs is a profound and persistent sadness, emptiness, or hopelessness that doesn’t lift. This may manifest as excessive crying, often without a clear trigger. Intense irritability, anger, or rage—directed at the partner, other children, or even the newborn—is another key indicator. Individuals may experience severe mood swings, far beyond typical hormonal fluctuations. A marked loss of interest or pleasure in activities once enjoyed, including sex, is a core symptom. Overwhelming anxiety or panic attacks are also common, often accompanied by debilitating worries about the baby's health or one's own inadequacy as a parent. Feelings of worthlessness, guilt, or shame, particularly about not being a "good enough" mother or not feeling the expected instant bond, are pervasive. Disturbingly, there may be recurrent thoughts of death, suicide, or harming oneself or the baby, which require immediate emergency attention.

**Cognitive and Behavioral Signs:** Significant changes in thinking and behavior are red flags. Difficulties with concentration, memory, and decision-making—often described as "brain fog"—make simple tasks feel insurmountable. Withdrawal from family, friends, and social support is common, leading to isolation. There may be a persistent lack of energy and extreme fatigue that doesn't improve with rest. Changes in sleep patterns are paradoxical: despite exhaustion, individuals may suffer from insomnia (even when the baby is sleeping), or conversely, they may sleep excessively. Appetite changes are also frequent, leading to significant weight loss or gain unrelated to postpartum recovery.

**Physical and Relational Signs:** PPD often manifests physically. Unexplained aches and pains, headaches, or stomach issues may occur. Most critically, there is a noticeable disruption in the ability to bond with or care for the newborn. This can feel like emotional numbness or detachment, and may be accompanied by excessive worry about the baby or, conversely, a disinterest in their needs. Relationship strain with a partner is almost universal, fueled by irritability, withdrawal, and feelings of being misunderstood.

**Key Differentiators from "Baby Blues":** While the "baby blues" involve mood swings, anxiety, and crying, these symptoms are milder, peak around 4-5 days postpartum, and typically resolve within two weeks without intervention. PPD symptoms are more severe, persistent, and debilitating. A critical warning sign is when these feelings *do not improve* or *worsen* after the first two weeks.

**Risk Factors and Action:** Certain factors increase vulnerability, including a personal or family history of depression or PPD, a difficult pregnancy or birth, lack of support, or a baby with special needs. Recognizing these signs in oneself or a loved one is the first step. It is imperative to seek help without shame or delay. PPD is not a character flaw or weakness; it is a medical complication of childbirth. Treatment—through therapy, support groups, medication, or a combination—is highly effective. Consulting an obstetrician, midwife, or mental health professional can lead to a proper diagnosis and a path toward recovery, allowing the individual to regain their well-being and fully engage in the parenting journey.

**9.How can partners/family recognize signs of postpartum depression?**

Partners and family members are often the first line of defense in recognizing postpartum depression (PPD), as the person experiencing it may be unable to identify or articulate their struggle. Recognition requires attentive observation of persistent changes that go beyond typical "baby blues," which usually fade within two weeks. Key signs to watch for fall into emotional, behavioral, and physical categories.

**Emotional and Behavioral Signs:**

The most telling signs are profound and persistent shifts in mood and outlook. This includes intense sadness, frequent crying spells, or severe mood swings that seem uncharacteristic. Partners may notice a pervasive sense of hopelessness, worthlessness, or overwhelming guilt, with the new parent making statements like "I'm a terrible mother" or "The baby would be better off without me." Extreme irritability or anger, often directed at the partner or other children, is a common but overlooked symptom. A marked loss of interest or pleasure in activities once enjoyed, including intimacy, is a red flag. Excessive anxiety or panic attacks, particularly about the baby's health or one's own parenting abilities, are also significant.

**Functional and Relational Changes:**

Observe how the individual is managing daily life. A clear sign is **withdrawal**—pulling away from partner, family, friends, and even the baby. They may seem emotionally disconnected or numb. Difficulties with bonding with the infant, such as expressing no affection or being unable to soothe the baby, can be symptomatic. Changes in sleep patterns are critical; while sleep deprivation is universal with a newborn, someone with PPD may be unable to sleep when the baby sleeps (insomnia) or may sleep excessively. Appetite changes—eating too little or too much—are also notable. A decline in personal hygiene and an inability to complete basic tasks can signal that things are amiss.

**Critical Warning Signs:**

Certain symptoms require **immediate professional intervention**. These include thoughts of harming oneself or the baby, any expression of suicidal ideation, or severe, disorganized thinking (psychosis). These are medical emergencies.

**What Partners and Family Can Do:**

Recognition must be followed by compassionate action. **Initiate a gentle, non-judgmental conversation.** Use "I" statements: "I've noticed you seem really sad lately, and I'm worried about you. How are you feeling?" **Validate their experience**—avoid minimizing ("Everyone feels tired") or offering simplistic solutions. **Encourage and facilitate help** by offering to make a doctor's appointment or arranging childcare so they can attend therapy. Most importantly, **share the load** concretely: take on household chores, manage night feeds where possible, and provide consistent emotional support. Remember, PPD is a medical condition, not a personal failing. By educating themselves, observing with care, and responding with support, loved ones play an indispensable role in guiding the new parent toward recovery.

**10.Can fathers/partners experience postpartum depression too?**

Yes, absolutely. Fathers and partners can and do experience postpartum depression (PPD), a reality that has gained significant clinical recognition over the past two decades, though it often remains overlooked in public discourse. While maternal postpartum depression is more widely known, research confirms that paternal postpartum depression (PPPD) is a genuine and serious condition, affecting an estimated **8-10% of new fathers**, with some studies suggesting rates can be as high as 25% during the first year postpartum. This prevalence highlights that the transition to parenthood is a profound psychological event for all caregivers, not just the birthing parent.

The causes of paternal PPD are multifaceted, stemming from a complex interplay of biological, psychological, and social factors. **Biologically**, new fathers can experience hormonal shifts, including drops in testosterone and increases in estrogen and prolactin, which are associated with caregiving. **Psychologically**, the massive life transition—sleeplessness, financial pressure, shifts in identity and relationship dynamics—can be overwhelming. Many partners feel a pressure to be the unwavering "rock" of the family, leading to suppressed emotions. Furthermore, witnessing a partner struggle with her own postpartum challenges or birth trauma can be a significant trigger. Social isolation is also a key factor; while new mothers often have support networks, partners may feel sidelined or expected to simply return to work without space to process their own adjustment.

Symptoms in partners can sometimes manifest differently than in mothers. While shared symptoms include sadness, loss of interest, fatigue, and anxiety, men are more likely to exhibit **externalizing behaviors** such as irritability, anger, increased conflict, risk-taking, escapist behavior (like overworking), and somatic complaints. This different presentation can lead to misdiagnosis or simply being labeled as "stressed" rather than clinically depressed.

The impact of untreated paternal depression is significant. It can affect the partner's bonding with the infant, increase conflict in the relationship, and is a known risk factor for prolonged maternal depression. Perhaps most critically, research indicates that a father's mental health is a strong predictor of a child's emotional and behavioral development.

Recognizing paternal postpartum depression is a crucial step toward family well-being. Treatment is effective and similar to that for maternal PPD: therapy (particularly cognitive-behavioral or couples therapy), support groups for new fathers, and, in some cases, medication. Normalizing this conversation is essential. By acknowledging that the postpartum journey is a shared emotional terrain, we can better support all parents, fostering healthier outcomes for the entire family unit. Encouraging partners to seek help is not a sign of weakness, but an act of strength and commitment to their new role.

**11.Can postpartum depression affect lactation or breastfeeding success?**

Yes, postpartum depression (PPD) can significantly affect both lactation and breastfeeding success, creating a challenging cycle that can impact both maternal and infant well-being. The relationship is complex and often bidirectional, with breastfeeding difficulties also contributing to the development or worsening of PPD.

**Physiological Impact on Lactation:**

PPD is associated with dysregulation of the neuroendocrine system, particularly the hormones critical for milk production and ejection. Chronic stress and depression can elevate cortisol levels, which may indirectly suppress prolactin, the primary hormone for milk synthesis. More notably, PPD often disrupts the oxytocin reflex, or "let-down." Oxytocin, the hormone responsible for milk ejection, is highly sensitive to emotional state. Feelings of anxiety, sadness, or stress can inhibit its release, making it physically difficult for milk to flow even if it is present in the breast. This can lead to a perceived low milk supply, frustrating feeding sessions, and inadequate milk transfer, which then further reduces supply due to insufficient breast stimulation.

**Behavioral and Psychological Impact on Breastfeeding:**

The symptoms of PPD directly interfere with the consistent practice required to establish and maintain lactation. Anhedonia and fatigue can make the demanding schedule of newborn feeding feel overwhelming. A mother may lack the motivation to pump or nurse frequently, leading to skipped feeds and insufficient breast emptying. Intense anxiety or feelings of detachment can disrupt the bonding process, making breastfeeding sessions stressful rather than rewarding. Furthermore, mothers with PPD may have negative thought patterns, doubting their ability to produce enough milk or to breastfeed successfully, which can erode breastfeeding self-efficacy—a key predictor of continuation.

**The Vicious Cycle:**

The interaction often becomes a vicious cycle. For example, physical difficulties like painful latch or poor milk transfer—potentially exacerbated by a tense mother-baby dyad—can lead to nipple pain, engorgement, or a fussy, unsatisfied infant. These problems increase maternal stress and feelings of failure, deepening depressive symptoms. Conversely, the early cessation of breastfeeding or supplementation (often introduced due to perceived insufficiency) can bring on feelings of guilt and inadequacy, worsening PPD. Research consistently shows that mothers with PPD are at a higher risk of shorter breastfeeding duration and lower rates of exclusive breastfeeding.

**Breaking the Cycle:**

Importantly, this cycle can be broken with appropriate support. Treating PPD is paramount and can improve breastfeeding outcomes. Healthcare providers should adopt an integrated approach, addressing both the mental health condition and lactation needs simultaneously. Support may include therapy, medication compatible with breastfeeding (many are safe), alongside targeted lactation support from a certified consultant who can assess latch, milk transfer, and supply. Peer support groups can also combat isolation and build confidence.

In conclusion, postpartum depression exerts a profound negative influence on lactation physiology and the breastfeeding behavioral process. Recognizing this link is crucial for providing compassionate, effective care. By offering concurrent mental health treatment and skilled lactation support, healthcare providers can help mothers navigate these intertwined challenges, promoting better outcomes for both their psychological health and their breastfeeding goals.

**12.Can postpartum depression affect a baby’s development?**

Yes, postpartum depression (PPD) can significantly affect a baby’s development across multiple domains—emotional, social, cognitive, and even physical. When a primary caregiver experiences depression, the essential, reciprocal interactions that form the foundation of healthy development are disrupted, with potential long-term implications.

The primary mechanism of impact is through the alteration of the **mother-infant bond and caregiving environment**. A mother with PPD often struggles with emotional availability, responsiveness, and consistent caregiving. She may have difficulty reading and responding to her baby’s cues, exhibit less facial expressiveness, reduced eye contact, and speak in a flatter tone. This breakdown in "serve-and-return" interaction—where a baby "serves" a cue (a smile, a cry) and the caregiver "returns" an appropriate response—is critical for brain development. Without it, the infant’s stress response system can become chronically activated, affecting the architecture of the developing brain.

Specifically, research links exposure to untreated maternal PPD to several developmental areas:

1. **Emotional and Social Development:** Infants may become more irritable, withdrawn, or anxious themselves. They often have difficulty regulating their own emotions and may exhibit attachment insecurity. This can set a trajectory for later challenges in forming healthy relationships, increased shyness, and a higher risk of internalizing problems like anxiety and depression in childhood and adolescence.

2. **Cognitive and Language Development:** The reduced quantity and quality of verbal interaction ("parentese," narrative talk) can delay language acquisition. Furthermore, the lack of engaged, playful stimulation can impact cognitive development, with some studies showing lower scores on developmental assessments and, in severe cases, a correlation with academic difficulties later in childhood.

3. **Physical and Behavioral Development:** In some cases, inconsistent caregiving can affect feeding and growth patterns. As children grow older, they may exhibit more externalizing behaviors, such as hyperactivity, impulsivity, or aggression, stemming from early self-regulation difficulties.

Crucially, these effects are **not inevitable**. They are influenced by the severity and duration of the depression, the presence of other supportive caregivers, and crucially, **whether the mother receives treatment**. Early intervention is paramount. Treating the mother’s depression—through therapy, social support, and sometimes medication—directly benefits the baby by restoring healthy interactions. Partner involvement, family support, and infant-parent psychotherapy can help repair the bond and mitigate risks.

In conclusion, postpartum depression is far more than a maternal health issue; it is a key influencer of the infant’s developmental environment. By disrupting the sensitive, responsive caregiving an infant needs, PPD can cast a long shadow on a child's emotional, social, and cognitive growth. This underscores the profound importance of universal screening for PPD, reducing stigma, and ensuring accessible treatment—not just for the mother’s well-being, but for the lifelong health and development of her child.

**13.What treatments are available for postpartum depression (therapy/meds/combined)?**

Postpartum depression (PPD) is a serious but treatable medical condition affecting many new parents, and a multifaceted approach to treatment is often most effective. The primary evidence-based treatments fall into three categories: psychotherapy, medication, and a combination of both, with the choice depending on the severity of symptoms, individual preference, and whether the parent is breastfeeding.

**Psychotherapy (Talk Therapy)** is frequently a first-line treatment, especially for mild to moderate PPD or for those who wish to avoid medication. Several specialized modalities have proven highly effective:

*	**Cognitive Behavioral Therapy (CBT):** Helps individuals identify and change negative thought patterns and behaviors that contribute to depression. It is practical and skill-based, teaching coping strategies for distressing emotions.

*	**Interpersonal Therapy (IPT):** Focuses on improving relationships and addressing role transitions, making it particularly relevant for the profound identity shift that accompanies parenthood. It helps with issues like communication with a partner or adjusting to new demands.

*	**Other supportive therapies,** including group therapy, can be invaluable by reducing isolation and providing peer support from others going through similar experiences.

**Medication** is often recommended for moderate to severe PPD, or when therapy alone is insufficient. The most commonly prescribed medications are **antidepressants**, particularly Selective Serotonin Reuptake Inhibitors (SSRIs) like sertraline (Zoloft) and paroxetine (Paxil), which are generally considered first-choice due to their established efficacy and safety profile during lactation. Other classes, such as Serotonin-Norepinephrine Reuptake Inhibitors (SNRIs), may also be used. It is crucial to have a detailed discussion with a psychiatrist or prescriber about benefits, potential side effects, and the very low concentration of medication that passes into breast milk. For severe, treatment-resistant cases, **Brexanolone (Zulresso)**, a 60-hour intravenous infusion, is an FDA-approved treatment specifically for PPD that works rapidly by modulating GABA receptors. A newer oral medication, **Zuranolone (Zurzuvae)**, is also now approved as a rapid-acting, short-course treatment.

**Combination Therapy (Psychotherapy + Medication)** is widely regarded as the **gold standard** for treating moderate to severe postpartum depression. Research consistently shows that combining medication and therapy is more effective than either treatment alone for many individuals. Medication can provide relatively quicker relief from the biological symptoms (like sleep disturbance, appetite changes, and intense anxiety), which then allows the person to more fully engage in and benefit from the psychological work of therapy. This integrated approach addresses both the neurochemical and psychosocial dimensions of PPD.

Beyond these core treatments, **lifestyle and support measures** are essential adjuncts. These include enlisting help with childcare and household tasks, prioritizing rest and nutrition, engaging in gentle physical activity when possible, and joining support groups. For many, a comprehensive treatment plan that may include therapy, medication, and strong social support offers the best path to recovery. It is vital for anyone experiencing symptoms to consult a healthcare provider—such as an OB/GYN, primary care physician, or mental health specialist—to create a personalized and effective treatment plan. With appropriate intervention, full recovery is not only possible but expected.

**14.Will I get PPD with a future pregnancy, and how can I prevent it?**

The possibility of experiencing postpartum depression (PPD) with a future pregnancy is a valid and common concern, especially if you have a history of PPD or other mood disorders. While there is no absolute certainty, understanding your risk and implementing a proactive prevention plan can significantly reduce both the likelihood and severity of PPD.

**Assessing Your Risk**

A history of PPD is the single strongest predictor, increasing your risk to approximately 30-50% in a subsequent pregnancy. Other significant risk factors include a personal or family history of depression or anxiety, experiencing significant stress during pregnancy or postpartum, a lack of social support, and hormonal sensitivity. However, it is crucial to remember that **risk is not destiny**. Many women with these risk factors do not develop PPD, and conversely, some without any known risk factors do. The key is not to fear the future but to prepare for it thoughtfully.

**A Multi-Layered Prevention Strategy**

Prevention is best viewed as building a robust support system and a personalized care plan, ideally starting **before conception**. This plan should involve the following layers:

1. **Pre-Pregnancy and Prenatal Planning:** Discuss your history openly with your OB/GYN or a psychiatrist **before you become pregnant**. They can help you weigh the risks and benefits of medication if you are currently on or may need antidepressants. Maintaining a healthy lifestyle with regular exercise, balanced nutrition, and adequate sleep establishes a strong physical foundation for mental health. Begin practicing stress-reduction techniques like mindfulness, meditation, or prenatal yoga to build resilience.

2. **Build Your Support System:** Identify your support network early. This includes your partner, family, and friends. Have frank conversations with them about your history and what you might need—whether it's help with meals, childcare for older siblings, or simply a listening ear. Consider joining a support group for mothers who have experienced PPD; their understanding can be invaluable. If finances allow, research postpartum doulas who provide non-medical, practical and emotional support after birth.

3. **Mental Health Care During Pregnancy:** Treat pregnancy as a period of "mental health prophylaxis." Continue or begin therapy, such as Cognitive Behavioral Therapy (CBT) or Interpersonal Therapy (IPT), which are effective in preventing depression relapse. Your healthcare provider may recommend more frequent check-ins to monitor your mood throughout pregnancy.

4. **The Postpartum Plan:** Develop a written postpartum plan with your partner and healthcare team. This should detail:

*	**Sleep Protection:** This is critical. Can you and your partner schedule shifts to ensure each of you gets a 4-5 hour block of uninterrupted sleep?

*	**Help Logistics:** Who will handle household chores? Who can you call at 2 a.m. if you're feeling overwhelmed?

*	**Self-Care Mandates:** Schedule short, non-negotiable breaks for yourself, even just 15 minutes alone.

*	**Monitoring and Action Steps:** Decide who (your partner, a family member) will help monitor your mood using a simple checklist (like the Edinburgh Postnatal Depression Scale). Pre-determine the signs that mean you should call your doctor immediately.

5. **Medical Management:** For those at very high risk, a healthcare provider may recommend starting or resuming an antidepressant immediately after delivery or even during the third trimester to create a protective buffer against the sudden hormonal shift.

In conclusion, while a history of PPD increases your statistical risk, it also gives you the powerful advantage of foreknowledge. You are not powerless. By creating a comprehensive, personalized prevention plan that addresses medical, psychological, and practical supports, you empower yourself to navigate a future pregnancy with greater confidence and significantly reduce your risk. If, despite all precautions, symptoms arise, view it not as a failure of your plan, but as a sign to immediately activate the treatment component of that plan. Seeking help early is the strongest possible action for your health and your family's well-being.

**15.Do exercise, sunlight, diet, and social support help with PPD?**

Yes, research consistently shows that **exercise, sunlight exposure, a nutritious diet, and strong social support** all play significant and beneficial roles in both preventing and managing Postpartum Depression (PPD). These non-pharmacological interventions work through interconnected biological and psychosocial pathways to improve mood and resilience during the postpartum period. They are widely recommended as foundational components of a holistic approach to perinatal mental health, often used alongside professional treatment when needed.

**The Role of Exercise**

Engaging in regular **physical activity**, particularly **aerobic exercise**, is a well-established strategy for mitigating PPD symptoms. Evidence suggests that exercise programs totaling **150 minutes or more per week** can have a significant antidepressant effect. The benefits are attributed to the release of endorphins, reduction of systemic inflammation, and the psychological boost gained from self-care. For new mothers, even manageable routines like **moderate-intensity walking for 90-120 minutes weekly** have been shown to be effective and sustainable.

**Sunlight Exposure and Vitamin D**

**Sunlight** and **vitamin D** are closely linked to mood regulation. Natural sunlight helps synchronize circadian rhythms, which are often disrupted with a newborn, and can boost serotonin levels. Clinically, **vitamin D deficiency** is a notable risk factor for PPD. Some meta-analyses indicate that low vitamin D levels could be associated with a substantially higher risk of developing PPD. Therefore, for many new mothers, having vitamin D levels checked and following a doctor's guidance on sensible sun exposure and supplementation (commonly around 2000 IU daily) can be a simple yet powerful supportive measure.

**The Importance of Diet and Specific Nutrients**

Overall diet quality and specific nutrient intake directly influence perinatal mental health. Diets rich in **vegetables, fruits, whole grains, lean protein, and healthy fats** (such as those found in fish, nuts, and avocados) help reduce neuroinflammation and provide the building blocks for neurotransmitters like serotonin. Key nutrients of particular importance include:

*	**Vitamin D:** As highlighted, its deficiency is a major concern.

*	**Iron and Folate:** Low levels of these are frequently associated with depressive symptoms in the postpartum period.

*	**Omega-3 Fatty Acids and B Vitamins:** These are crucial for brain function and the synthesis of mood-regulating chemicals.

**The Power of Social Support**

Robust **social support** is arguably one of the most critical protective factors against PPD. It buffers stress, reduces feelings of isolation and being overwhelmed, and provides practical help. Support can be emotional (listening, validation), instrumental (help with chores or childcare), or informational (sharing knowledge). Research confirms that interventions like **psychoeducation for parents, peer support groups, and counseling from healthcare providers** are effective in reducing PPD risk and severity. Building this support network is vital both **during pregnancy and after childbirth**.

**Practical Integration of Strategies**

For new mothers, integrating these elements can be practical and synergistic:

1. **Combine Activities:** A daily **15-20 minute walk with the baby** outdoors provides exercise, sunlight exposure, and a break from the home environment.

2. **Prioritize Nutrient-Dense Foods:** Focus on incorporating a variety of colorful vegetables, fruits, whole grains, and lean proteins into meals. A continued postnatal vitamin can help fill nutritional gaps.

3. **Proactively Build Your Support System:** Clearly communicate needs with your partner, family, and friends before and after delivery. Seek out local or online **new parent groups** to connect with others.

4. **Consult Healthcare Professionals:** Always discuss persistent low mood or anxiety with a doctor, midwife, or mental health specialist. They can provide personalized advice, check for nutrient deficiencies, and recommend appropriate therapy or treatment if necessary.

In conclusion, while PPD is a complex condition that sometimes requires clinical treatment such as therapy or medication, **regular exercise, mindful sunlight exposure, a nutrient-rich diet, and strong social connections** are powerful, evidence-based tools that can significantly support emotional well-being and recovery in the postpartum period.

**16.What can family do most effectively to prevent and treat postpartum depression?**

Of all the factors influencing postpartum depression (PPD), a supportive family environment is arguably the most powerful. To prevent and treat PPD effectively, family members must move beyond general encouragement and adopt a proactive, practical, and sustained partnership with the new mother. Their role is not to diagnose or cure, but to create a scaffold of care that protects her well-being and facilitates professional help when needed.

For **prevention**, the family’s most effective tool is **practical and anticipatory support**. This means physically lightening her load before she breaks under it. Partners and family should co-create a "postpartum plan" that prioritizes rest, nutrition, and manageable expectations. This includes: sharing night feeds (using pumped milk or formula), taking over household chores, managing visitor schedules, and ensuring the mother has uninterrupted time to sleep. Crucially, family must also **validate her emotions** without judgment, listening to her fears and frustrations rather than dismissing them as "baby blues." Education is key; if the family learns the symptoms of PPD (persistent sadness, anxiety, withdrawal, irritability) they can monitor with compassion rather than alarm.

When it comes to **treatment**, the family’s role shifts to **active advocacy and unwavering support**. The most critical action is to **encourage and enable professional help**. This means helping her find a therapist or psychiatrist, taking on childcare so she can attend appointments, and normalizing medication if prescribed. Family must combat stigma by reiterating that PPD is a medical condition, not a character flaw.

Concurrently, they must **hold space for her recovery** by maintaining the practical support indefinitely. Recovery is not linear; a mother may have good days followed by difficult ones. Family should avoid phrases like "just snap out of it" and instead offer specific help: "I’ve got the baby for the next two hours—you can rest, shower, or take a walk." Partners should also prioritize their own mental health to avoid caregiver burnout and model healthy coping.

Ultimately, the most effective approach is a **family-wide strategy of "mothering the mother."** By assuming logistical and emotional burdens, the family creates a safe container for the mother to heal. This team-based care protects the maternal-infant bond, reduces feelings of isolation, and significantly improves outcomes. Preventing and treating PPD is not the mother’s solitary duty; it is a testament to a family’s collective resilience, where love is expressed not just in words, but in shared responsibility and informed, patient action.

**17.When should a patient with postpartum depression seek professional help?**

A patient with postpartum depression (PPD) should seek professional help **as soon as they suspect their experience has moved beyond the typical "baby blues."** While up to 80% of new mothers experience transient mood swings, fatigue, and weepiness (the baby blues) in the first two weeks, PPD is more intense, persistent, and debilitating. The overarching rule is: **if symptoms last longer than two weeks, significantly interfere with daily functioning, or include thoughts of harming oneself or the baby, immediate professional help is non-negotiable.**

Specific signs that warrant professional intervention include:

1. **Persistent Emotional Symptoms:** Overwhelming sadness, hopelessness, or emptiness that doesn’t lift. Intense irritability, anger, or rage—often directed at partners, other children, or even the baby. Severe anxiety, panic attacks, or constant worrying, especially about the baby’s health or one’s own competence as a mother.

2. **Behavioral and Cognitive Changes:** A marked inability to care for oneself or the baby. Withdrawing completely from family, friends, and activities once enjoyed. Extreme fatigue coupled with insomnia (even when the baby is sleeping). Significant changes in appetite. Difficulty concentrating, remembering details, or making decisions. Feelings of worthlessness, guilt, or shame, with intrusive thoughts like "I'm a bad mother."

3. **Loss of Connection:** A disturbing sense of emotional numbness or detachment from the baby, feeling as though you are just going through the motions without bonding.

4. **Thoughts of Harm:** **This is a critical emergency.** Having thoughts of harming oneself or, much more rarely, the infant. This includes passive thoughts like "Everyone would be better off without me," or active suicidal ideation. These symptoms require **immediate intervention** from a healthcare provider, a crisis line, or an emergency room.

It is also crucial to seek help earlier rather than later if there are risk factors, such as a history of depression or PPD, a lack of support system, or significant life stressors. The negative impact of untreated PPD extends beyond the mother; it can affect infant bonding, cognitive development, and family dynamics.

**How to Seek Help:** The first point of contact can be an obstetrician, midwife, or the baby’s pediatrician—all are trained to screen for PPD. They can provide referrals to mental health professionals specializing in perinatal mood disorders, such as psychiatrists, psychologists, or licensed therapists. Treatment is highly effective and typically involves psychotherapy (like Cognitive Behavioral Therapy or Interpersonal Therapy), sometimes combined with antidepressant medication considered safe during breastfeeding.

In summary, the threshold for seeking help is low by design. No symptom is too small, and suffering in silence is neither necessary nor safe. Seeking professional guidance is an act of profound strength and care, ensuring the well-being of both the mother and her new family. If in doubt, the safest course is always to reach out.

**18.Can postpartum depression go away without treatment?**

Postpartum depression (PPD) is a serious and complex medical condition that affects an estimated 1 in 7 new parents. The central question of whether it can go away without treatment does not have a simple yes or no answer. While it is **possible for some individuals to experience a gradual, natural remission of symptoms**, this is not the norm, nor is it a path without significant risks. Relying on spontaneous recovery is generally not advisable and can lead to prolonged suffering and potential complications.

In a minority of cases, particularly those with very mild, subclinical symptoms that may align more with the "baby blues," the depression may lift as the body undergoes massive hormonal adjustments in the first few weeks postpartum. Factors like a robust pre-existing support system, excellent self-care, stable circumstances, and the natural passage of time can contribute to an unaided improvement. However, what often appears to be "going away on its own" may instead be the individual learning to mask or function with the symptoms, which can lead to chronic, low-grade depression.

The significant dangers of leaving PPD untreated must be emphasized. **Untreated PPD can last for months or even years**, evolving into a chronic depressive disorder. The ramifications extend far beyond the affected parent. It can severely impair the crucial bonding process (attachment) between parent and infant, which is foundational for the child’s emotional, social, and cognitive development. Research indicates potential risks for the child, including increased likelihood of future behavioral, emotional, and learning difficulties. For the parent, the constant struggle can affect relationships, overall quality of life, and in severe cases, lead to suicidal or infanticidal ideation—a true medical emergency.

Furthermore, the notion of "waiting it out" disregards the profound personal cost. The postpartum period is a time that should involve connection and joy, albeit with challenges. PPD robs individuals of this experience, replacing it with guilt, sadness, and numbness. Every day spent waiting is a day lost.

Therefore, while spontaneous remission is a biological possibility, **active treatment is the safest, most effective, and fastest route to recovery.** Treatment is multifaceted and not limited to medication. Psychotherapy, particularly Cognitive Behavioral Therapy (CBT) and Interpersonal Therapy (IPT), has proven highly effective. Peer support groups can reduce isolation and provide practical coping strategies. In some cases, hormonal treatments may be considered. Crucially, treatment is also about building a toolkit to manage the challenges of new parenthood, making the individual more resilient in the long term.

In conclusion, one should not gamble with postpartum depression. The potential for it to resolve without professional intervention is uncertain and carries too high a stake for both the parent and the child. Seeking help is a sign of strength and a proactive step toward health. If you or someone you know is experiencing symptoms of PPD—such as persistent sadness, anxiety, hopelessness, irritability, or thoughts of harming oneself or the baby—**contact a healthcare provider, a mental health specialist, or a support hotline immediately.** Recovery is not only possible but probable with the right support, allowing families to thrive.

**19.Can postpartum depression relapse after stopping meds, and what’s the recurrence risk?**

Yes, postpartum depression (PPD) can absolutely relapse after stopping medication, and the recurrence risk is significant. This is a critical concern for individuals planning future pregnancies or managing their long-term mental health after an initial episode.

**Relapse After Stopping Medication**

Stopping antidepressant medication, especially abruptly or without professional guidance, is a common trigger for relapse. Postpartum depression is not simply a time-limited condition that resolves permanently; it is a major depressive episode that occurs in the perinatal period. When medication is effective, it manages the biological and neurological symptoms of depression. Discontinuing it removes that support, potentially allowing symptoms to re-emerge if the underlying vulnerability persists. This is particularly true if the medication was stopped before completing a full, clinically recommended course of treatment. For individuals with a history of PPD, the postpartum period itself—with its dramatic hormonal shifts, sleep deprivation, and new stressors—creates a high-risk environment for relapse, even years after an initial episode.

**Recurrence Risk: The Statistics**

The recurrence risk for postpartum depression is substantial and well-documented:

*	For any subsequent pregnancy, the risk of experiencing a new episode of PPD is estimated to be **30-50%**. This means a person with a prior history has a one-in-three to one-in-two chance of experiencing it again.

*	This risk increases dramatically with a personal history of **non-perinatal** major depression, raising the recurrence rate in the postpartum period to as high as **25-40%**.

*	The strongest predictor is the number of prior depressive episodes. Those with multiple episodes before or after previous pregnancies face the highest likelihood of recurrence.

**Key Risk Factors for Recurrence**

Several factors amplify the risk of relapse or recurrence:

1. **History of Depression:** A prior episode of PPD or any major depressive disorder is the single most significant risk factor.

2. **Discontinuation of Medication:** Stopping medication prematurely (often defined as less than 6-12 months of maintenance therapy after symptom remission) greatly increases relapse risk.

3. **Timing of Weaning:** For breastfeeding parents, the act of weaning can itself trigger a depressive episode due to the sudden drop in prolactin and other hormones, mimicking a mini-postpartum hormonal shift.

4. **Psychosocial Stressors:** Lack of social support, marital conflict, financial stress, and a high-needs infant continue to be major contributing factors.

5. **Anxiety Comorbidity:** The presence of significant anxiety symptoms during or after the initial PPD episode is linked to a more severe and chronic course.

**Clinical Recommendations and Prevention**

Given these risks, a proactive and preventive approach is essential:

*	**Maintenance Therapy:** Current guidelines strongly recommend continuing antidepressant medication for **at least 6 to 12 months after full remission of symptoms** to consolidate recovery and prevent relapse. For those with a history of multiple episodes, long-term maintenance may be advised.

*	**Gradual Tapering:** Any discontinuation of medication must be done slowly, under a doctor's supervision, to monitor for early warning signs of relapse.

*	**Preconception Planning:** Individuals with a history of PPD should discuss a prevention plan with their healthcare provider *before* a subsequent pregnancy. This may involve starting or resuming medication during pregnancy or immediately postpartum.

*	**Multimodal Support:** Medication should be combined with ongoing psychotherapy (e.g., CBT, IPT), strong social support networks, and lifestyle management to build resilience.

*	**Vigilant Monitoring:** Both the individual and their support system should be educated on early warning signs (irritability, sleep changes, anxiety) to enable rapid intervention.

In conclusion, while postpartum depression is treatable, it carries a high risk of recurrence, particularly if medication is stopped without a strategic plan. Recognizing this allows for informed decision-making, personalized treatment, and proactive measures to safeguard maternal mental health during the vulnerable postpartum period and beyond. Management should always be individualized in close consultation with a psychiatrist or specialized healthcare provider.

**20.How do you differentiate postpartum depression from postpartum PTSD—and can they co-occur?**

Distinguishing between postpartum depression (PPD) and post-traumatic stress disorder following childbirth (postpartum PTSD) is crucial for accurate diagnosis and effective treatment, as they have distinct core features despite some overlapping symptoms. They can and frequently do co-occur, creating a more complex clinical picture.

**Postpartum Depression (PPD)** is primarily characterized by persistent and pervasive low mood, anhedonia (loss of interest or pleasure), and a range of symptoms that align with major depressive disorder, but with onset during pregnancy or within the first year after childbirth. Its hallmark features include intense sadness, hopelessness, guilt, worthlessness, and emotional numbness. Physical symptoms like significant changes in appetite or sleep (unrelated to infant care), overwhelming fatigue, and irritability are common. A mother with PPD may have severe anxiety, but it often centers on generalized worries about the baby’s health or her own inadequacy as a parent. Critically, PPD is not necessarily linked to a specific traumatic event; it can arise from hormonal shifts, psychological adjustment, stress, or a personal history of depression.

**Postpartum PTSD**, on the other hand, is a trauma-specific anxiety disorder triggered by a real or perceived threat to the life or physical integrity of the mother or her infant during childbirth or the immediate postpartum period. Its symptoms cluster into four categories as defined by the DSM-5: 1) **Re-experiencing** the trauma through intrusive memories, flashbacks, or nightmares; 2) **Avoidance** of anything associated with the trauma (e.g., avoiding the hospital, not discussing the birth); 3) **Negative alterations in mood and cognition** (e.g., persistent fear, horror, anger, guilt, or feeling detached from the baby); and 4) **Hyperarousal**, including hypervigilance, exaggerated startle response, and irritability. The focus is persistently on the traumatic event itself.

**Key Differentiators:**

*	**Etiology:** PPD may not have a distinct "cause," while postpartum PTSD is explicitly linked to a subjectively traumatic event.

*	**Core Symptom:** PPD's core is depressed mood and loss of pleasure. Postpartum PTSD's core is fear and anxiety rooted in re-experiencing a trauma.

*	**Thought Content:** PPD thoughts are often about worthlessness ("I'm a bad mother"). Postpartum PTSD thoughts are about danger and the past trauma ("Something terrible happened/I am not safe").

*	**Relationship with Baby:** In PPD, bonding difficulties often stem from apathy or lack of energy. In postpartum PTSD, they may stem from avoidance or fear triggered by the baby (if the baby is associated with the trauma).

**Co-Occurrence**

These conditions can absolutely co-occur, and research suggests comorbidity is common. A traumatic birth experience can act as a direct trigger for both disorders simultaneously. The physiological stress response to trauma, combined with the drastic hormonal shifts postpartum, creates a fertile ground for both PTSD and depressive symptoms. For instance, a mother who experienced a life-threatening emergency delivery may develop PTSD with flashbacks and avoidance, while also sinking into a major depressive episode characterized by hopelessness and guilt about the birth outcome. The symptoms can exacerbate one another—avoidance and social withdrawal from PTSD can deepen depression, while the fatigue and negativity of depression can make it harder to process trauma.

In conclusion, while PPD is a mood disorder and postpartum PTSD is an anxiety disorder rooted in trauma, their shared context of the perinatal period and similar presentations (irritability, sleep disturbance, guilt) necessitate careful clinical assessment. Recognizing the difference is essential for targeted intervention—trauma-focused therapies for PTSD versus a combination of psychotherapy and possibly medication for PPD. When they co-occur, an integrated treatment approach addressing both the depressive symptoms and the traumatic memories is required for healing.

Gemini

**1.What is Postpartum Depression?**

Postpartum depression (PPD) is a complex mix of physical, emotional, and behavioral changes that occur in some women after giving birth. It is a serious form of major clinical depression that is distinct from the "baby blues," a condition affecting up to 80% of new mothers that typically involves mild mood swings, crying spells, and anxiety which resolve on their own within two weeks.

PPD, by contrast, involves more intense and longer-lasting symptoms that can interfere with a mother’s ability to care for herself and her baby. It generally emerges within the first few weeks after delivery, but symptoms can begin up to six months postpartum. It is critical to understand that PPD is a medical condition, not a character flaw or a weakness.

\### Common Symptoms

The experience of PPD varies, but it often encompasses a range of severe symptoms that persist for more than two weeks:

* **Emotional Instability:** Feelings of overwhelming sadness, emptiness, or hopelessness.

* **Anxiety:** Severe anxiety, panic attacks, or excessive worry about the baby's health.

* **Detachment:** Difficulty bonding with the baby or withdrawing from family and friends.

* **Physical Changes:** profound fatigue, changes in appetite, or sleep disturbances (insomnia or sleeping too much) unrelated to the baby’s schedule.

* **Intrusive Thoughts:** In severe cases, mothers may have thoughts of harming themselves or the baby.

\### Causes and Risk Factors

There is no single cause for postpartum depression; it is likely a combination of physical and emotional factors.

* **Hormonal Changes:** After childbirth, a dramatic drop in the hormones estrogen and progesterone may contribute to chemical changes in the brain that trigger mood swings.

* **Sleep Deprivation:** The exhaustion caused by caring for a newborn can culminate in physical discomfort and exhaustion, exacerbating depressive symptoms.

* **Emotional Factors:** The psychological adjustment to parenthood, identity changes, and feelings of loss of control can also play significant roles.

\### Treatment and Recovery

Postpartum depression is highly treatable. Recovery often involves a combination of strategies:

* **Psychotherapy:** Cognitive Behavioral Therapy (CBT) and interpersonal therapy are effective in helping mothers cope with feelings and solve problems.

* **Medication:** Antidepressants may be prescribed to help balance brain chemicals.

* **Support Networks:** Joining support groups can reduce feelings of isolation.

With proper diagnosis and treatment, most women recover completely and bond successfully with their babies.

> **Important Note:** If you or someone you know is experiencing symptoms of postpartum depression, especially thoughts of harm, please consult a healthcare provider immediately.

**Would you like me to explain the specific differences between "baby blues" and postpartum depression in more detail?**

**2.What are the typical symptoms of postpartum depression?**

Postpartum depression (PPD) is a complex mix of physical, emotional, and behavioral changes that happen in some women after giving birth. It is a form of major depression that typically arises within four weeks of delivery, though it can surface up to a year later. While it is widely recognized as a serious medical condition, the symptoms can vary significantly in intensity and duration from person to person.

Emotional and Psychological Symptoms

The hallmark of postpartum depression is a persistent feeling of sadness, emptiness, or hopelessness. Unlike the fleeting mood swings associated with the "baby blues," these feelings are intense and do not fade after a week or two. Common emotional indicators include:

- Severe Anxiety and Panic: Many individuals experience excessive worry or irrational fears, often concerning the baby's health or their ability to parent. This can escalate into panic attacks.
- Irritability and Anger: A short temper, agitation, or rage that seems out of character is a frequent symptom.
- Guilt and Worthlessness: Feelings of inadequacy or the belief that one is a "bad mother" are pervasive.
- Loss of Interest: A distinct lack of joy in activities that were previously pleasurable, often referred to as anhedonia.

Physical and Cognitive Changes

Depression affects the body and mind physically, often creating a cycle that exacerbates emotional distress.

- Sleep Disturbances: While new parents naturally lose sleep, those with PPD may have trouble sleeping even when the baby is asleep (insomnia) or, conversely, may sleep excessively (hypersomnia).
- Appetite Changes: Significant weight loss or gain unrelated to the pregnancy recovery, often driven by a loss of appetite or emotional eating.
- Fatigue: An overwhelming, bone-deep exhaustion that does not resolve with rest.
- Brain Fog: Difficulty concentrating, making decisions, or remembering details is common.

Behavioral Signs

PPD can also manifest in how a parent interacts with their infant and others.

- Withdrawal: Pulling away from partners, family, and friends.
- Difficulty Bonding: Feeling detached from the baby, or feeling as though the baby is not one's own.
- Intrusive Thoughts: In some cases, individuals may have frightening, unwanted thoughts about harming themselves or the baby. This is a medical emergency requiring immediate attention.

Distinction from "Baby Blues"

It is critical to distinguish PPD from the "baby blues," which affect up to 80% of new mothers. The blues typically involve mild mood swings, crying spells, and anxiety, but these symptoms usually peak around the fourth day after birth and resolve within two weeks. If symptoms persist longer than two weeks or interfere with daily functioning, it is likely postpartum depression.

> Medical Disclaimer: I am an AI, not a doctor. This information is for educational purposes and does not constitute medical advice. If you or someone you know is experiencing symptoms of postpartum depression, please consult a healthcare professional immediately.

Would you like me to look up resources or support organizations for postpartum depression?

**3.What are the hazards of postpartum depression?**

Postpartum depression (PPD) is a complex and serious mood disorder that extends far beyond the "baby blues." It is a significant medical condition that can have profound, multi-dimensional hazards affecting not only the mother but also the infant, the partner, and the entire family unit. Left untreated, the consequences can be severe and long-lasting.

Impact on Maternal Health and Safety

The most immediate hazard is to the mother’s well-being. PPD can be debilitating, causing extreme fatigue, anxiety, and a sense of hopelessness that makes daily functioning nearly impossible.

- Chronic Depression: If untreated, PPD can evolve into a chronic, recurring major depressive disorder that persists for years.
- Physical Health: The stress of the condition often leads to sleep disturbances (beyond normal newborn waking), poor nutrition, and a weakened immune system.
- Risk of Suicide: In its most severe forms, PPD is a life-threatening condition. Suicide is a leading cause of maternal mortality in the year following childbirth.
- Postpartum Psychosis: While rare, severe PPD can escalate to postpartum psychosis, a medical emergency characterized by hallucinations, delusions, and a risk of harm to self or the baby.

Hazards to the Infant

The relationship between a mother and her infant is critical for the child's development, and PPD can severely disrupt this bond.

- Attachment Issues: PPD can impair a mother's ability to respond to her baby's cues (crying, smiling, hunger). This lack of responsiveness can lead to insecure attachment, where the infant does not feel safe or understood.
- Developmental Delays: Studies suggest that children of mothers with untreated PPD may face higher risks of behavioral problems, cognitive delays, and emotional instability later in childhood.
- Physical Neglect: In cases where the depression causes severe apathy or withdrawal, there is a hazard of unintentional neglect regarding the infant's feeding, hygiene, or safety.

Strain on the Family Unit

PPD creates a ripple effect that touches everyone in the household. Partners of women with PPD are statistically at a much higher risk of developing depression themselves. The strain can lead to relationship breakdowns, separation, or divorce, further destabilizing the home environment. Older siblings may also experience confusion or anxiety due to the change in the family dynamic and the mother's withdrawal.

The Importance of Intervention

The hazards of postpartum depression are preventable and treatable. It is not a character flaw or a sign of weakness; it is a chemical and hormonal imbalance. Early intervention through therapy, medication, and support groups can mitigate these risks effectively, protecting the health of both the mother and the child.

Disclaimer: I am an AI, not a doctor. If you or someone you know is in crisis, please seek professional medical help immediately.

Would you like me to find specific contact information for maternal mental health hotlines or support organizations?

**4.When does postpartum depression usually start? How long does postpartum depression last?**

### Understanding Postpartum Depression: Onset and Duration

Postpartum depression (PPD) is a complex mood disorder associated with childbirth. Unlike the "baby blues," which are mild and short-lived, PPD is a more severe and persistent condition that requires attention. Understanding its timeline is crucial for recognizing symptoms and seeking appropriate support.

#### When Does It Usually Start?

The onset of postpartum depression is not always immediate. While many people assume it begins right after delivery, the timeline can vary significantly between individuals.

- The First Few Weeks: Most commonly, symptoms of PPD begin within the first few weeks after giving birth. This often overlaps with the "baby blues," making it difficult to distinguish initially. However, while the blues fade, PPD symptoms intensify.
- Delayed Onset: PPD does not always strike immediately. It can develop up to a year after childbirth. This is sometimes referred to as "late-onset" postpartum depression. A parent might feel fine for several months, only to experience symptoms later due to hormonal shifts, weaning from breastfeeding, or the return of a menstrual cycle.
- During Pregnancy: Recent research suggests that for many, symptoms actually begin during pregnancy (perinatal depression) and continue into the postpartum period.
- How Long Does It Last?

There is no single timeline for how long postpartum depression lasts, as it is highly dependent on the severity of the condition and, most importantly, whether treatment is sought.

1. Treated vs. Untreated

- With Treatment: With appropriate medical care—such as therapy, medication, or a combination of both—many people see a significant improvement in symptoms within weeks. Full recovery is common within a few months. Early intervention is the most significant factor in shortening the duration of the illness.
- Without Treatment: Left untreated, PPD can last for months or even years. It can evolve into a chronic depressive disorder that persists long after the postpartum period has ended. Some studies suggest that approximately 30% of women who do not seek treatment are still depressed up to three years after giving birth.

2. The "Baby Blues" Distinction

It is vital to distinguish PPD from the "baby blues." The baby blues typically start 2–3 days after birth and last for a maximum of two weeks. If feelings of sadness, anxiety, or emptiness persist beyond two weeks, it is clinically considered a marker for postpartum depression rather than just hormonal adjustment.

#### Factors Influencing Duration

Several factors can influence how long the condition persists:

History of Depression: A personal or family history of depression can lead to longer recovery times.

Social Support: A strong network of support can significantly shorten the duration of symptoms.

Stressors: ongoing life stressors, such as financial difficulties or relationship issues, can prolong the condition.

Medical Disclaimer: I am an AI, not a doctor. If you or a loved one are experiencing symptoms of postpartum depression, please consult a healthcare professional.

Would you like me to find a list of common symptoms to look out for, or perhaps resources for professional support?

**5.What are the risk factors for postpartum depression?**

Postpartum depression (PPD) is a complex condition influenced by a combination of physical, emotional, and lifestyle factors. It is not caused by a single event or action, and it can affect any parent regardless of age, race, income, or educational background. While the exact causes are not fully understood, research has identified several key risk factors that can increase susceptibility.

History of Mental Health Challenges

One of the strongest predictors for PPD is a personal or family history of mental health conditions.

- Previous PPD: Individuals who have experienced postpartum depression in a previous pregnancy are significantly more likely to experience it again.
- History of Depression or Anxiety: A pre-existing diagnosis of depression, anxiety, or bipolar disorder, either during the pregnancy (prenatal depression) or at any other time in life, raises the risk.
- Family History: Having family members who have struggled with depression or other mood disorders suggests a potential genetic vulnerability.
- Hormonal and Physical Changes

The rapid physiological changes associated with childbirth play a major role.

- Hormonal Fluctuations: After delivery, the dramatic drop in hormones like estrogen and progesterone can trigger chemical changes in the brain that may lead to mood swings.
- Thyroid Levels: A temporary drop in thyroid hormones postpartum can mimic symptoms of depression, causing fatigue, sluggishness, and irritability.
- Physical Recovery: The physical toll of labor, delivery complications, recovery from C-sections, or managing breastfeeding challenges can contribute to exhaustion and emotional strain.

Life Stressors and Social Support

External circumstances significantly impact mental health during the postpartum period.

- Lack of Support: Isolation or having a weak support system from partners, family, or friends is a major risk factor.
- Financial Stress: Worries about finances, job stability, or the cost of raising a child can be overwhelming.
- Relationship Issues: Conflict with a partner or marital instability can exacerbate feelings of sadness and anxiety.
- Complications: Health problems with the baby or a difficult pregnancy/delivery can add traumatic stress to the experience.

It is important to remember that having one or more of these risk factors does not guarantee that a person will develop postpartum depression, but being aware of them can help in early identification and seeking support.

Would you like me to find information on local support groups or resources for postpartum mental health?

**6.How do you tell postpartum blues from postpartum depression?**

Distinguishing between the "baby blues" and postpartum depression requires looking closely at the intensity of the emotions, how long they last, and how deeply they affect a parent's ability to function. While both experiences often stem from the dramatic hormonal shifts and exhaustion that follow childbirth, they are fundamentally different in their severity and their trajectory.

The "baby blues" are an incredibly common and transient response to birth, experienced by a majority of new parents. This phase typically begins two to three days after delivery. A parent might find themselves weeping over a commercial on television, feeling irritable with their partner, or experiencing sudden waves of anxiety or sadness. The defining characteristic of the blues is that, while uncomfortable, these feelings are temporary and manageable. They do not generally prevent a parent from bonding with the baby or taking care of daily needs. Crucially, the blues resolve on their own without medical treatment, usually fading within two weeks as hormones stabilize and routines are established.

Postpartum depression, on the other hand, is a distinct and treatable medical condition that is far more intense and persistent. It is not merely a "bad mood" but a pervasive mood disorder that can surface days or even months after birth. The symptoms go beyond tearfulness to include deep feelings of hopelessness, severe anxiety, and a frightening sense of detachment from the baby or others. A parent with postpartum depression may feel unable to experience joy, have trouble eating or sleeping regardless of the baby's schedule, or struggle with intrusive thoughts of harm. Unlike the blues, these feelings do not simply vanish after a few weeks; they linger and often worsen, significantly interfering with a parent's ability to care for themselves or their infant.

The most reliable way to tell the difference is to watch the calendar. The "two-week rule" is a standard guideline: if symptoms of sadness or anxiety persist longer than two weeks postpartum, or if they begin later than the immediate postpartum window, it is likely not just the blues. Additionally, if the feelings are intense enough to disrupt daily life—making it hard to get out of bed, shower, or care for the baby—this functional impairment is a strong indicator of depression rather than the blues. Recognizing these distinctions is vital because while the blues require patience and rest, postpartum depression requires professional support and care to resolve.

Would you like me to explain the different types of professional support available, such as therapy or medication options?

**7.Does a prior history of depression or anxiety increase PPD risk?**

The Connection Between Mental Health History and PPD Risk

Yes, a prior history of depression or anxiety is widely considered one of the strongest predictors for developing Postpartum Depression (PPD). While PPD can affect anyone regardless of their medical background, research consistently shows that individuals who have previously experienced mood or anxiety disorders are at a statistically higher risk than the general population.

Understanding this link is not about generating fear, but rather about empowering expecting parents and healthcare providers to create effective support plans.

Understanding the Risk Factors

The relationship between past mental health challenges and PPD is multifaceted, often involving a combination of biological, psychological, and environmental factors.

- History of Depression: Women with a history of Major Depressive Disorder (MDD) before pregnancy are significantly more likely to experience PPD. The risk is even higher if the depression was active during the pregnancy itself (antenatal depression). The physiological stress of childbirth, combined with the rapid drop in hormones like estrogen and progesterone, can act as a trigger for recurrence in those with a biological vulnerability to mood shifts.
- History of Anxiety: While depression often gets the spotlight, anxiety is an equally critical risk factor. A history of Generalized Anxiety Disorder (GAD) or high levels of anxiety during pregnancy can predispose a mother to PPD. Furthermore, PPD often presents with severe anxiety symptoms, meaning a history of anxiety can sometimes evolve into a complex depressive-anxious postpartum state.
- Previous PPD: The strongest single predictor is having had PPD in a previous pregnancy. In these cases, the recurrence rate is substantial, often estimated to be between 30% and 50%.

Why History Matters

The transition to parenthood is a period of intense vulnerability. Sleep deprivation, physical recovery from birth, and the immense responsibility of caring for a newborn act as major stressors. For an individual with a history of mental health struggles, these stressors can overwhelm coping mechanisms that might otherwise be sufficient.

However, it is crucial to note that risk is not destiny. Having a history of depression or anxiety does not guarantee that someone will develop PPD. Instead, it serves as a vital signal for proactive care.

The Value of Early Awareness

Knowing that this risk exists allows for preventative measures that can drastically alter the outcome. When healthcare providers are aware of a patient's history, they can:

- Monitor mood changes more closely during the third trimester and immediately after birth.
- Implement screenings earlier than the standard six-week checkup.
- Help establish a support network (therapy, medication, or social support) before the baby arrives.

Key Takeaways

- Significant Predictor: Past depression or anxiety is a major risk factor for PPD.
- Antenatal Health: Depression during pregnancy is also a strong indicator of postpartum risk.
- Preparation is Key: Identifying this risk early allows for the creation of a robust postpartum care plan.

Would you like to explore specific preventative strategies or how to build a postpartum support plan?

**8.What are early warning signs of postpartum depression?**

Postpartum depression (PPD) is a complex mix of physical, emotional, and behavioral changes that happen in some women after giving birth. It is a serious mental health condition that is distinct from the "baby blues," which are milder and typically resolve within a few weeks. Recognizing the early warning signs is crucial for ensuring that parents receive the support and treatment they need.

Distinguishing PPD from "Baby Blues"

It is common for new mothers to feel moody, tearful, or overwhelmed in the first few days after childbirth due to drastic hormonal shifts. These feelings, often called the "baby blues," usually peak around the fourth day and subside within two weeks.

Key Difference: If feelings of sadness or anxiety persist beyond two weeks, intensify, or interfere with your ability to care for yourself or your baby, it may be an early sign of postpartum depression.

Common Early Warning Signs

The symptoms of PPD can vary in intensity and may appear within a few weeks of delivery or up to six months later. Key early indicators include:

- Persistent Sadness or Empty Mood: A feeling of sadness, hopelessness, or emptiness that does not go away, even when there are happy moments.
- Difficulty Bonding with the Baby: Feeling indifferent towards the newborn, or feeling as though the baby is not "yours." This can lead to intense guilt, further fueling the depression.
- Withdrawal and Isolation: Pulling away from partners, family, and friends, and losing interest in activities that were previously enjoyable.
- Intense Irritability and Anger: Experiencing severe mood swings, distinct frustration, or uncontrollable anger, often directed at the partner or other children.
- Changes in Appetite and Sleep: Eating too little or too much, and struggling to sleep (insomnia) even when the baby is sleeping, or conversely, sleeping too much.
- Overwhelming Fatigue: While new parents are naturally tired, PPD can cause a level of exhaustion that does not improve with rest.
- Feelings of Worthlessness or Guilt: An unshakable belief that you are a "bad mother" or that you are failing your family.
- Severe Anxiety: Excessive worrying that something bad will happen to the baby, racing thoughts, or panic attacks.

Immediate Red Flags

In severe cases, a person may experience thoughts of harming themselves or the baby. This is a medical emergency. If these thoughts occur, it is vital to seek immediate assistance from a healthcare provider or emergency services.

When to Seek Professional Help

Postpartum depression is a medical condition, not a character flaw or a weakness. It is treatable with therapy, support groups, and sometimes medication. If you or a loved one notice these warning signs persisting for more than two weeks, or if they make it difficult to cope with daily life, consulting a healthcare professional is the most important step toward recovery.

Disclaimer: I am an AI, not a doctor. This information is for educational purposes and does not constitute medical advice. If you have concerns about your health, please consult a medical professional.

Would you like me to find resources for support groups or mental health hotlines?

**9.How can partners/family recognize signs of postpartum depression?**

Recognizing postpartum depression (PPD) is a crucial role for partners and family members, primarily because the person experiencing it may be too overwhelmed or exhausted to realize that what they are feeling goes beyond normal fatigue. While many new parents experience the "baby blues" (mood swings and tearfulness) for a week or two after birth, PPD is distinct in its intensity, duration, and impact on daily functioning.

Distinguishing PPD from the "Baby Blues"

The most significant indicator is the timeline. The "baby blues" typically resolve within two weeks. If symptoms persist longer than two weeks, intensify rather than improve, or begin several weeks or months after childbirth, it may be PPD.

Partners should look for emotional shifts that seem out of character. While sadness is a common symptom, PPD often presents as severe anxiety or rage. You might notice:

- Intense Irritability or Anger: Snapping at loved ones over minor issues or expressing sudden rage.
- Numbness: A lack of feeling or a sense of detachment, often described as "going through the motions" without joy.
- Persistent Anxiety: An inability to relax, even when the baby is sleeping or well-cared for.

Behavioral and Physical Signs

Changes in behavior are often easier to spot than internal feelings. Pay attention to sleep disturbances that aren’t related to the baby’s schedule. For example, if the parent cannot sleep even when the baby is sound asleep, this is a major red flag. Conversely, wanting to sleep all the time and struggling to get out of bed is also common.

Other behavioral signs include:

- Withdrawal: Pulling away from the partner, family, or friends, and avoiding social interactions they previously enjoyed.
- Difficulty Bonding: Showing little interest in the baby, or conversely, being obsessively worried about the baby's health to the point of panic.
- Cognitive Fog: Struggling to make simple decisions, concentrate, or remember details.

Scary or Intrusive Thoughts

One of the most distressing symptoms for parents is the presence of intrusive thoughts. A partner might not "see" this, but they might hear the parent express fears like, "I’m not a good parent," "My family would be better off without me," or fears of accidentally harming the baby. If a loved one voices these thoughts, it requires immediate supportive attention.

Important Note: PPD is a treatable medical condition, not a character flaw. Recognizing these signs early allows the family to encourage the parent to seek professional help, which is the most effective path to recovery.

Would you like me to suggest specific ways to start a supportive conversation if you suspect a loved one is struggling?

**10.Can fathers/partners experience postpartum depression too?**

Yes, fathers and partners can absolutely experience postpartum depression. While often discussed in the context of birthing mothers, Paternal Postpartum Depression (PPPD) is a very real and significant clinical condition that affects a substantial number of men and non-birthing partners. Research suggests that approximately 1 in 10 fathers experience depression during the perinatal period, a number that can rise to 50% if their partner is also struggling with depression.

How It Manifests Differently

One reason PPPD often goes undiagnosed is that it can look different from "classic" depression. While sadness and low mood are common, men specifically may exhibit symptoms that lean more towards behavioral changes rather than purely emotional ones. These can include:

- Irritability and Anger: A shorter fuse, sudden outbursts, or increased frustration.
- Withdrawal: Pulling away from social relationships, the partner, or the baby.
- Risk-Taking: Engaging in reckless behaviors, substance use, or working excessive hours to avoid being at home.
- Physical Symptoms: Unexplained headaches, digestion issues, or pain.

Why It Happens

The causes of PPPD are a complex mix of biological, environmental, and psychological factors. It is not "all in the head"; fathers also undergo hormonal changes during and after pregnancy, including drops in testosterone and increases in estrogen and prolactin. Combined with the severe sleep deprivation that comes with a newborn, the financial pressure of a growing family, and the shifting dynamics of the romantic relationship, the environment is ripe for mental health challenges.

The Importance of Recognition

Acknowledging PPPD is crucial for the health of the entire family unit. When a partner is depressed, they are less able to support the birthing parent and bond with the infant, which can impact the child's long-term emotional and behavioral development. Treatment is highly effective and similar to maternal depression, involving therapy, support groups, and sometimes medication.

Next Step

Would you like me to find a list of common resources or support organizations specifically for fathers dealing with mental health challenges?

**11.Can postpartum depression affect lactation or breastfeeding success?**

Yes, there is a significant and complex relationship between postpartum depression (PPD) and breastfeeding success. Research consistently suggests that the relationship is bidirectional, meaning that PPD can negatively impact breastfeeding, and conversely, difficulties with breastfeeding can increase the risk of developing PPD.

The Physiological Impact

From a biological standpoint, maternal mental health directly influences the hormones required for lactation. Breastfeeding relies heavily on two hormones: prolactin (which produces milk) and oxytocin (which triggers the "let-down" reflex that allows milk to flow).

When a mother experiences the high levels of stress, anxiety, or depression associated with PPD, her body releases higher levels of cortisol. Cortisol can inhibit the release of oxytocin, potentially delaying or suppressing the let-down reflex. This does not necessarily mean the body isn't producing milk, but it can make it difficult for the baby to access it effectively. This often leads to a distressed infant, which in turn increases the mother's stress, creating a difficult cycle that may lead to the perception of low milk supply.

The Behavioral and Psychological Impact

Depression affects a mother’s energy levels, motivation, and self-efficacy (belief in her own ability to succeed). Women suffering from PPD may struggle with the intense, round-the-clock demands of breastfeeding. They may experience:

- Lower Self-Efficacy: Mothers with PPD often report lower confidence in their ability to breastfeed, which is a strong predictor of early weaning.
- Reduced Interaction: Depression can sometimes dampen the emotional reward cues usually received during nursing, making the process feel more like a draining chore than a bonding experience.
- Disrupted Routines: The fatigue and sleep disturbances associated with depression can make maintaining a consistent feeding or pumping schedule difficult, which can eventually lead to a genuine decrease in milk supply due to lack of stimulation.

The "Vicious Cycle"

Perhaps the most critical aspect to understand is the feedback loop. When breastfeeding challenges arise—such as latch issues, pain, or concern about the baby's weight gain—a mother may feel intense guilt or a sense of failure. In vulnerable individuals, these feelings can trigger or worsen postpartum depression. Conversely, a mother already struggling with PPD may interpret normal newborn behaviors (like cluster feeding) as a sign that she is starving her baby, leading to unnecessary supplementation or early cessation of breastfeeding.

Breaking the Link

It is important to note that many women with PPD breastfeed successfully, and for some, the release of oxytocin during nursing can actually have a protective, mood-lifting effect. However, support is vital. Lactation consultants and healthcare providers now emphasize that treating the mother's mental health is a critical component of preserving the breastfeeding relationship.

Disclaimer: I am an AI, not a doctor. This information is for educational purposes only and does not constitute medical advice. If you or someone you know is struggling with postpartum depression or breastfeeding challenges, please consult a healthcare professional.

Would you like me to look up resources or helplines for postpartum support?

**12.Can postpartum depression affect a baby’s development?**

Yes, postpartum depression (PPD) can significantly affect a baby’s development. Research consistently shows that the mental health of a primary caregiver is intrinsically linked to an infant's growth, particularly in the realms of emotional, behavioral, and cognitive development.

It is important to approach this topic with compassion. PPD is a medical condition, not a choice or a parenting failure, and while the potential effects are real, they are often treatable and reversible with support.

The Mechanism: The "Serve and Return" Dynamic

Infants rely heavily on interaction to wire their brains. Development experts often refer to the "serve and return" interaction style: a baby coos or smiles (serves), and the parent responds with eye contact, a smile, or a voice (returns).

Mothers or parents suffering from PPD may struggle with symptoms like fatigue, emotional numbness, or irritability. This can lead to inconsistent responsiveness. If a parent is withdrawn or has a "flat" affect (lack of facial expression), the baby may not receive the social cues necessary to build secure attachment and emotional regulation skills.

Key Areas of Potential Impact

- Emotional and Behavioral Regulation: Babies are highly sensitive to their caregivers' emotional states. Infants of mothers with untreated PPD may be more prone to excessive crying, fussiness, or, conversely, withdrawal and passivity. As they grow into toddlers, they may exhibit higher levels of anxiety or difficulty managing stress.
- Cognitive and Language Development: Because early learning is social, a reduction in verbal interaction—such as singing, reading, or talking to the baby—can correlate with delays in language acquisition. Studies have suggested that children of mothers with severe, untreated depression may perform slightly lower on cognitive readiness tests upon entering school.
- Sleep and Feeding: The stress associated with PPD can sometimes disrupt routine establishment. This may manifest as difficulties with breastfeeding or irregular sleep patterns for the infant, which in turn can exacerbate the mother's exhaustion, creating a challenging cycle.

Resilience and Recovery

Crucially, these effects are not inevitable. The impact on the child is largely dependent on the severity and duration of the depression.

- Buffering Factors: The presence of other loving, engaged caregivers (like a partner, grandparent, or sibling) can buffer the baby against negative effects.
- Treatment Works: When a mother seeks treatment—whether through therapy, medication, or support groups—her symptoms often improve, and the developmental trajectory of the child can align with their peers. Treating the mother is treating the child.

If you or someone you know is concerned about PPD, professional support can make a profound difference for the entire family.

Would you like me to find resources for PPD support or information on early infant bonding activities?

**13.What treatments are available for postpartum depression (therapy/meds/combined)?**

Treatment for postpartum depression (PPD) typically falls into three primary categories: psychotherapy, medication, and combination approaches. The choice of treatment often depends on the severity of symptoms, the mother's medical history, and personal preferences regarding breastfeeding.

1. Psychotherapy (Counseling)

For mild to moderate PPD, psychotherapy is often the first line of defense. It provides a safe space to express feelings and develops coping strategies without the potential side effects of medication.

- Cognitive Behavioral Therapy (CBT): This is one of the most effective forms of therapy for PPD. It focuses on identifying and changing negative thought patterns and behaviors. For a new mother, this might involve challenging unrealistic expectations about "perfect" parenting.
- Interpersonal Therapy (IPT): This therapy focuses on your relationships and social support systems. It helps navigate the massive role transition of becoming a parent and addresses relationship conflicts that may be contributing to the depression.

2. Medications

Medication is often recommended for moderate to severe cases, or when therapy alone hasn't provided relief.

- SSRIs (Selective Serotonin Reuptake Inhibitors): These are the most commonly prescribed antidepressants (e.g., sertraline/Zoloft, fluoxetine/Prozac). Sertraline is frequently the first choice for breastfeeding mothers because very little of the drug passes into breast milk. However, SSRIs typically take 2–4 weeks to start working.
- Neurosteroids (Rapid-Acting Options):
- Zuranolone (Zurzuvae): Approved by the FDA in 2023, this is the first oral pill specifically for PPD. Unlike SSRIs, it is a 14-day course designed to provide rapid relief (often within days). Note: Current guidelines generally recommend suspending breastfeeding during the 14-day treatment.
- Brexanolone (Zulresso): This was the first drug approved specifically for PPD but requires a 60-hour intravenous (IV) infusion under medical supervision in a hospital.

3. Combined Treatment

For moderate to severe PPD, a combination of medication and therapy is often considered the "gold standard." This approach attacks the condition from two angles: medication helps regulate the biological imbalances (making you feel stable enough to engage in therapy), while therapy addresses the psychological triggers and life changes. Clinical guidelines generally suggest that combined treatment offers a higher chance of remission than either method alone for severe symptoms.

Other Treatments

In cases where PPD is resistant to standard treatments or involves psychosis (a rare emergency), doctors may consider Electroconvulsive Therapy (ECT). While it carries stigma, it is a safe and highly effective medical procedure for severe, refractory depression. Additionally, support groups are a vital adjunct to clinical treatment, reducing the isolation that often fuels PPD.

Would you like me to find a list of support resources or helplines specifically for postpartum mental health?

**14.Will I get PPD with a future pregnancy, and how can I prevent it?**

Disclaimer: I am an AI, not a doctor or mental health professional. The following information is for educational purposes only and should not replace professional medical advice, diagnosis, or treatment. Please consult with your healthcare provider regarding your specific medical history and risks.

Predicting whether you will experience Postpartum Depression (PPD) in a future pregnancy is not possible with 100% certainty, as every pregnancy and postpartum experience is unique. However, understanding the risk factors—particularly your personal medical history—can help you and your healthcare provider create a robust plan to mitigate those risks.

### Understanding the Risk of Recurrence

The strongest predictor of PPD is a history of depression. Medical research indicates that if you have experienced PPD in a previous pregnancy, the risk of recurrence is significantly higher than that of the general population. Estimates suggest that women with a history of PPD have a recurrence risk ranging from 25% to 50% (and potentially higher if there is a history of chronic depression outside of pregnancy).

Other factors that may influence your risk include:

- Hormonal Sensitivity: Some individuals are more sensitive to the rapid drop in estrogen and progesterone that occurs after birth.
- Life Stressors: Financial strain, relationship difficulties, or lack of a strong support system can exacerbate risk.
- Sleep Deprivation: Severe sleep loss is a major trigger for mood disorders.
- Strategies for Prevention and Management

While you may not be able to completely eliminate the risk, you can significantly lower the likelihood or severity of PPD through proactive planning.

#### 1. Proactive Medical Planning

Don't wait until the baby arrives to address mental health.

- Pre-Conception/Prenatal Consult: Speak to your OB-GYN or a psychiatrist before or during pregnancy. Discuss your history openly.
- Preventative Therapy: Engaging in psychotherapy, such as Cognitive Behavioral Therapy (CBT) or Interpersonal Therapy (IPT), during pregnancy has been shown to reduce the incidence of PPD.
- Medication Plan: For some, starting medication immediately after delivery (or continuing it during pregnancy) is recommended by doctors to prevent a crash.

#### 2. The "Postpartum Pact"

Create a written plan with your partner and support network that outlines specific logistics. This reduces mental load when you are exhausted.

- Protected Sleep: Sleep fragmentation is a primary driver of PPD. Work out a shift system with your partner or a family member so you can get at least one unbroken stretch of 4–5 hours of sleep.
- Delegation: assign specific tasks (cooking, cleaning, grocery runs) to friends or family members before the baby is born.

#### 3. Nutrition and Physical Wellness

- Nutrient Support: Focus on a diet rich in Omega-3 fatty acids (found in fish or supplements), which are linked to brain health.
- Gentle Movement: Once cleared by a doctor, light exercise like walking can boost endorphins and regulate mood.

### Summary

While the possibility of recurrence is real, it is not a guarantee. Many women who go on to have subsequent pregnancies do not experience PPD again, especially when they have a preventative care plan in place. By treating your mental health with the same priority as your physical health, you build a safety net that protects both you and your baby.

Would you like me to help you draft a list of questions to ask your doctor at your next appointment regarding preventative mental health care?

**15.Do exercise, sunlight, diet, and social support help with PPD?**

Yes, research indicates that lifestyle factors such as exercise, exposure to sunlight, a balanced diet, and strong social support can play a significant role in managing Postpartum Depression (PPD). While these strategies are rarely considered a standalone cure for moderate to severe PPD, they are powerful adjunctive treatments—meaning they work best when paired with professional care like therapy or medication.

Here is a breakdown of how each factor contributes to recovery:

1. Exercise

Physical activity is one of the most effective non-medical interventions for depression. For new mothers, exercise does not necessarily mean intense gym sessions; even low-impact activities like walking with a stroller can be beneficial.

- Mechanism: Exercise stimulates the release of endorphins and neurotransmitters like serotonin and dopamine, which help regulate mood.
- Impact: It reduces stress hormones (cortisol), improves sleep quality (often a major struggle for new parents), and provides a sense of routine and accomplishment.

2. Sunlight and Light Exposure

Getting outside, particularly in the morning, helps regulate the body's internal clock (circadian rhythm), which is often disrupted by caring for a newborn.

- Vitamin D: Sunlight triggers the skin to produce Vitamin D. Low levels of Vitamin D have been linked to higher risks of depression and anxiety.
- Serotonin Boost: Exposure to bright natural light increases serotonin levels in the brain, which is associated with improved mood and focus. For those in darker climates, bright light therapy lamps are sometimes recommended as an alternative.

3. Diet and Nutrition

The postpartum period places high nutritional demands on the body, especially if recovering from surgery (C-section) or breastfeeding.

- Blood Sugar Stability: A diet high in processed sugars can lead to energy crashes and mood swings. Complex carbohydrates and proteins help maintain stable blood sugar levels.
- Key Nutrients: Research suggests that Omega-3 fatty acids (found in fatty fish, walnuts, and flaxseeds) may have a protective effect against PPD. Adequate hydration is also critical, as dehydration can mimic symptoms of fatigue and anxiety.

4. Social Support

Perhaps the most critical non-biological factor is social connection. PPD thrives in isolation.

- Emotional Validation: Speaking with other parents or supportive friends helps normalize the difficulties of early parenthood, reducing feelings of shame or inadequacy.
- Practical Help: "Social support" isn't just talking; it is also receiving help with chores, cooking, or holding the baby so the mother can sleep. This practical assistance directly lowers stress levels.

Important Note: While these lifestyle changes are helpful, PPD is a medical condition, not a failure of will or lifestyle. If symptoms are severe or interfering with daily functioning, professional medical advice is essential.

Would you like me to find information on professional resources or support groups for PPD?

**16.What can family do most effectively to prevent and treat postpartum depression?**

Family members play a crucial, often primary, role in both the prevention and treatment of postpartum depression (PPD). Because the partner and close relatives are typically the "first responders" in a new mother's life, their actions can significantly mitigate risk factors and accelerate recovery.

1. Proactive Practical Support (Prevention)

One of the most effective ways family can help prevent PPD is by alleviating the sheer physical exhaustion that triggers mental health struggles. Sleep deprivation is a major contributor to mood disorders.

- Protect Her Sleep: Partners or family members should take over specific night feeds or early morning shifts to ensure the mother gets uninterrupted blocks of sleep (ideally 4–5 hours).
- Manage the Household: Instead of asking "What can I do?", family should proactively handle cooking, cleaning, and laundry. Removing the mental load of household management allows the mother to focus solely on recovery and bonding.
- Gatekeeping Visitors: Well-meaning visitors can be draining. A partner can act as a gatekeeper, limiting visits to ensure the mother isn't overwhelmed by the social obligation to "host."

2. Emotional Observation and Validation (Detection)

Family members are often the first to notice the subtle shifts that differentiate the "baby blues" from PPD.

- Watch for Red Flags: Be alert for signs such as withdrawal from family, lack of interest in the baby, excessive irritability, or expressions of hopelessness. If these persist beyond two weeks, it is likely more than just the blues.
- Validate Feelings: Create a safe space where she can express negative emotions without judgment. Avoid toxic positivity phrases like "At least the baby is healthy" or "You should be happy." Instead, use validating language: "It makes sense that you are exhausted and overwhelmed. This is really hard."

3. Encouraging Professional Treatment (Intervention)

If PPD develops, family support is vital in ensuring treatment is sought and maintained. Depression can make the logistical steps of finding a doctor feel impossible.

- Facilitate Care: Family members should take the initiative to research therapists, make appointments, and drive the mother to them.
- Normalize Help: Combat stigma by treating mental healthcare as just another part of postpartum recovery, no different than a physical check-up.
- Medication Support: If medication is prescribed, support her decision and help monitor for side effects or improvements, reassuring her that taking care of her mental health is taking care of her baby.

Key Takeaway

The most effective strategy is a combination of relentless practical help to reduce stress and non-judgmental emotional vigilance to catch symptoms early. By functioning as a protective buffer against exhaustion and isolation, the family creates the best possible environment for mental wellness.

Would you like to know more about the specific warning signs that differentiate the "baby blues" from postpartum depression?

**17.When should a patient with postpartum depression seek professional help?**

Recognizing the difference between the "baby blues" and postpartum depression (PPD) can be difficult, but knowing when to reach out is critical for the health of both the parent and the child.

A patient should seek professional help immediately if they experience any of the following scenarios:

1. Symptoms Persist Beyond Two Weeks

It is common to feel moody, tearful, or overwhelmed in the first few days after delivery due to hormonal shifts. However, these "baby blues" typically resolve within 10 to 14 days. If feelings of sadness, emptiness, or hopelessness last longer than two weeks, it is a primary indicator of PPD, and a healthcare provider should be consulted.

2. Symptoms Interfere with Daily Functioning

Professional help is necessary if the depression affects the ability to perform basic daily tasks or care for the infant. Warning signs include:

- Inability to care for the baby: Feeling disconnected, indifferent, or resentful toward the infant, or feeling incapable of meeting the baby's needs.
- Inability to care for oneself: Skipping meals, refusing to shower, or severe sleep disturbances (sleeping too much or being unable to sleep even when the baby is sleeping).
- Social Withdrawal: Isolating completely from partners, family, and friends.

3. Intensity of Anxiety or Hopelessness

If the feelings are intense rather than mild, help is needed. This includes severe anxiety, panic attacks, racing thoughts, or an overwhelming sense of guilt and worthlessness. If a patient feels like they are "going crazy" or that the family would be better off without them, this requires professional intervention.

4. Immediate Emergency Situations

Immediate emergency care (calling emergency services or visiting an ER) is mandatory if there are:

- Thoughts of harm: Any thoughts of harming oneself or the baby.
- Signs of Postpartum Psychosis: This is a rare medical emergency characterized by hallucinations (seeing or hearing things), delusions (false beliefs), paranoia, or extreme confusion.

Summary

Postpartum depression is a medical condition, not a character flaw. It is highly treatable with therapy, support groups, and medication. Early intervention leads to faster recovery, so if a patient is in doubt, it is always safer to schedule an appointment with an OB-GYN, primary care doctor, or mental health professional to discuss their symptoms.

Would you like me to list specific international or local hotlines and support resources for postpartum mental health?

**18.Can postpartum depression go away without treatment?**

While it is technically possible for postpartum depression (PPD) to improve over time without formal medical intervention, relying on it to "go away" on its own is risky and generally not recommended by medical professionals. To understand why, it is essential to distinguish between the "baby blues" and clinical PPD, and to understand the potential consequences of leaving the condition untreated.

Distinguishing PPD from the "Baby Blues"

The belief that postpartum depression will resolve naturally often stems from confusion with the "baby blues." The baby blues affect up to 80% of new mothers and include mood swings, crying spells, and anxiety. These symptoms typically begin a few days after delivery and resolve on their own within two weeks as hormone levels stabilize.

Postpartum depression is different. It is a serious mental health condition that is more intense and lasts longer. If symptoms persist beyond two weeks, or if they interfere with the ability to care for the baby or handle daily tasks, it is classified as PPD. Unlike the blues, PPD is caused by a complex combination of physical, emotional, and lifestyle factors that rarely resolve quickly without support.

The Risks of Untreated PPD

While some studies suggest that a depressive episode might eventually remit (subside) on its own after many months or even a year, "waiting it out" carries significant dangers:

- Chronicity: Untreated PPD can evolve into chronic Major Depressive Disorder. A mother may continue to struggle with depression for years after the birth of her child if the cycle is not broken.
- Impact on Child Development: PPD can affect the mother-infant bond. Babies need consistent emotional interaction for healthy brain development. When a mother is withdrawn or unresponsive due to depression, it can lead to emotional, behavioral, and cognitive delays in the child.
- Family Strain: The condition often places immense strain on partnerships and the wider family unit, potentially leading to isolation and relationship breakdown.
- Escalation: In severe cases, untreated PPD can escalate. It may lead to thoughts of self-harm or, in rare instances, progress to postpartum psychosis, which is a medical emergency.

Treatment Speeds Recovery

The primary argument for treatment is not just safety, but the reduction of suffering. With professional help—which can range from therapy (like Cognitive Behavioral Therapy) and support groups to medication—recovery is often much faster and more complete. Treatment helps restore the mother’s quality of life and her ability to bond with her infant during a critical window of time.

In summary, while spontaneous remission is theoretically possible over a long timeline, the risks to the mother's long-term health and the baby's development make "waiting and seeing" a dangerous approach.

Disclaimer: I am an AI, not a doctor. If you or a loved one are struggling with symptoms of postpartum depression, please consult a healthcare provider. If you are in crisis, please contact emergency services or a dedicated crisis line immediately.

Would you like me to find resources for PPD support groups or crisis lines in your specific region?

**19.Can postpartum depression relapse after stopping meds, and what’s the recurrence risk?**

Yes, postpartum depression (PPD) can absolutely relapse after stopping medication. In fact, the risk of recurrence is significant, particularly if the medication is stopped abruptly or too soon after symptoms have improved.

It is common to feel "cured" once symptoms vanish, leading many to believe they no longer need treatment. However, antidepressants often act as a protective buffer, and removing them prematurely can leave the brain vulnerable to the hormonal fluctuations and stressors inherent in early motherhood.

### The Risk of Recurrence

Research indicates that the risk of relapsing is substantially higher for women who discontinue medication compared to those who stay on it.

- High Relapse Rates: Studies suggest that women who stop antidepressants—especially during or shortly after pregnancy—face a relapse rate as high as 60% to 70%. In contrast, those who maintain their treatment often see significantly lower relapse rates (closer to 25–30%).
- Severity Matters: The risk is not uniform for everyone. Women with a history of severe depression, multiple prior episodes, or "treatment-resistant" depression are at the highest risk. If you have had previous episodes of depression before pregnancy, the likelihood of PPD returning after stopping meds increases.
- The "Cliff" Effect: Relapse often happens relatively quickly after discontinuation. One study found that the majority of relapses occurred within the first few months of stopping the medication.

### Why Does Relapse Happen?

Several factors drive this recurrence risk beyond just the chemical changes:

- Withdrawal vs. Relapse: Stopping medication suddenly can cause "discontinuation syndrome," which mimics depression (irritability, crying spells, fatigue). This can sometimes trigger a full depressive episode.
- Biological Vulnerability: Postpartum bodies are still undergoing massive hormonal regulation (estrogen, progesterone, and thyroid levels) for months after birth. Medication often helps stabilize mood during this chaotic biological window.
- Environmental Triggers: The postpartum period is uniquely stressful due to sleep deprivation, physical recovery, and the demands of childcare. Medication often provides the resilience needed to handle these stressors; without it, the threshold for becoming overwhelmed drops.

### Reducing the Risk

If you are considering stopping your medication, it is vital to do so under medical supervision.

- Tapering is key: Slowly lowering the dose over weeks or months can drastically reduce the shock to your system.
- Wait for stability: Guidelines often suggest remaining on medication for at least 6–12 months after you feel fully recovered to ensure the remission is stable.
- Therapy as a bridge: deeply engaging in psychotherapy (like CBT) before and during the weaning process can act as a protective scaffold, lowering relapse risk.

Disclaimer: I am an AI, not a doctor. Please consult your healthcare provider before making any changes to your medication regimen, as sudden changes can be dangerous.

**20.How do you differentiate postpartum depression from postpartum PTSD—and can they co-occur?**

While both Postpartum Depression (PPD) and Postpartum Post-Traumatic Stress Disorder (PTSD) are serious mental health conditions that can arise after childbirth, they stem from different psychological roots and present with distinct symptom profiles. Understanding the nuances between them is vital for receiving the correct support and treatment.

The Core Distinction: Mood vs. Trauma

The primary difference lies in the central feature of the distress: Postpartum Depression is primarily a mood disorder, whereas Postpartum PTSD is an anxiety disorder rooted in a specific traumatic event.

- Postpartum Depression (PPD): PPD is characterized by pervasive feelings of sadness, hopelessness, and worthlessness. Mothers may feel a severe disconnect from their baby, struggle with intense guilt, or experience a loss of interest in things they once enjoyed. The dominant emotions are often heaviness, lethargy, and despair. It is roughly analogous to Major Depressive Disorder but occurs during the perinatal period.
- Postpartum PTSD: This condition is explicitly triggered by a traumatic birth experience (real or perceived). This could involve an emergency C-section, a baby being whisked away to the NICU, feelings of powerlessness during labor, or a fear of death for oneself or the child. The dominant emotions are fear, hyperarousal, and anxiety.

Symptom Overlap and Differences

While both conditions can cause sleep disturbances and irritability, the content of these symptoms differs significantly:

- Flashbacks vs. General Rumination: A hallmark of PTSD is intrusive re-experiencing of the trauma. This includes flashbacks, nightmares about the birth, and physical panic reactions when reminded of the event. In PPD, a mother might ruminate on her perceived failure as a parent, but she does not typically "relive" a specific moment of physical danger.
- Avoidance: Women with Postpartum PTSD often go to great lengths to avoid triggers associated with the birth. They might avoid the hospital where they delivered, refuse to talk about the birth, or even struggle to bond with the baby because the infant is a reminder of the trauma. PPD avoidance is usually due to a lack of energy or overwhelming apathy rather than fear.
- Hypervigilance: PTSD often manifests as being "on edge" or constantly scanning for danger, fearing something terrible will happen again. PPD is more likely to manifest as a "shut down" response or mental fog.

Can They Co-Occur?

Yes, Postpartum Depression and Postpartum PTSD frequently co-occur.

Research indicates a high rate of comorbidity between the two. A traumatic birth experience can be the catalyst for PTSD, and the subsequent difficulty in healing, bonding, or sleeping caused by PTSD symptoms can precipitate a depressive episode. Conversely, the exhaustion and isolation of depression can make a person more vulnerable to the lasting impacts of trauma.

When they co-occur, the clinical picture can be complex. A mother might feel the "flatness" of depression while simultaneously experiencing the heart-racing panic of PTSD flashbacks. This combination can be particularly debilitating because the avoidance behaviors of PTSD can prevent a mother from seeking help for her depression, and the lethargy of depression can make the active work of trauma therapy feel impossible.

Disclaimer: I am an AI, not a doctor. If you or someone you know is experiencing these symptoms, please consult a healthcare professional for a proper diagnosis and treatment plan.

Would you like me to explain the different treatment approaches typically used for these conditions?

Claude

**1.What is postpartum depression?**

Postpartum depression (PPD) is a serious mental health condition that affects some people after childbirth. Unlike the "baby blues"—which involve mild mood swings, crying spells, and anxiety that typically resolve within two weeks—postpartum depression is more severe, longer-lasting, and can significantly interfere with daily functioning and bonding with the baby.

PPD typically develops within the first few weeks after delivery, though it can emerge anytime during the first year postpartum. The condition involves persistent feelings of sadness, hopelessness, and emptiness. People with postpartum depression may experience intense anxiety, irritability, or anger. They might feel overwhelmed, unable to cope with caring for their baby, or worry excessively about the infant's health and wellbeing. Some experience difficulty bonding with their newborn or have intrusive, frightening thoughts about harming themselves or the baby.

Physical symptoms often accompany the emotional ones, including changes in sleep patterns (sleeping too much or being unable to sleep even when the baby sleeps), appetite changes, fatigue, and loss of interest in activities once enjoyed. Concentration and decision-making can become difficult, and feelings of worthlessness or guilt are common.

The causes of postpartum depression are complex and multifaceted. Dramatic hormonal changes after delivery play a role, as levels of estrogen and progesterone drop sharply. However, other factors contribute significantly: sleep deprivation, the physical demands of recovery from childbirth, previous history of depression or anxiety, lack of social support, stressful life events, and the overwhelming adjustment to new responsibilities.

Postpartum depression is treatable. Treatment typically involves therapy (particularly cognitive-behavioral therapy or interpersonal therapy), support groups, and sometimes medication. It's important to seek help from healthcare providers, as untreated PPD can affect both the parent's wellbeing and the child's development. With proper treatment, most people recover fully.

**2.What are the typical symptoms of postpartum depression?**

Postpartum depression (PPD) is a serious mental health condition that affects many new parents, typically developing within the first few weeks to months after childbirth. Understanding its symptoms is crucial for early recognition and treatment

**Emotional Symptoms**

The emotional manifestations of PPD go far beyond typical "baby blues." Individuals may experience persistent sadness, emptiness, or hopelessness that doesn't lift. Many describe feeling emotionally numb or disconnected from their baby, which can be particularly distressing. Severe anxiety is common, often manifesting as excessive worry about the baby's health or one's ability to care for them. Some people experience intense irritability, anger, or mood swings that feel disproportionate to the situation. Feelings of worthlessness, guilt, or shame about not feeling the "expected" maternal joy are also characteristic.

**Physical and Behavioral Symptoms**

PPD affects the body as well as the mind. Sleep disturbances are common—either difficulty sleeping even when the baby sleeps, or sleeping excessively. Appetite changes may occur, with some people eating much less or more than usual. Energy levels typically plummet, making even basic tasks feel overwhelming. Many people experience difficulty concentrating, making decisions, or remembering things. Some withdraw from family and friends, losing interest in activities they once enjoyed.

**Severe Symptoms**

In more severe cases, individuals may have recurrent thoughts about death or suicide, or thoughts of harming themselves or their baby. These thoughts require immediate professional attention.

**Duration and Impact**

Unlike the "baby blues," which resolve within two weeks, PPD symptoms persist for weeks or months and significantly interfere with daily functioning. The severity and combination of symptoms vary widely between individuals.

It's important to note that experiencing PPD doesn't reflect personal weakness or inadequacy as a parent. It's a medical condition that responds well to treatment, including therapy, medication, or both. Anyone experiencing these symptoms should reach out to a healthcare provider promptly, as early intervention leads to better outcomes for both parent and child.

**3.What are the hazards of postpartum depression?**

Postpartum depression (PPD) is a serious mental health condition that poses significant hazards to both mothers and their families. Understanding these risks is crucial for early identification and intervention.

**Hazards to Maternal Health**

The most severe hazard is the increased risk of suicide and self-harm. Women with PPD experience profound despair, hopelessness, and intrusive thoughts that can become dangerous. This represents a leading cause of maternal mortality in the postpartum period. Additionally, PPD can trigger or worsen other mental health conditions, including anxiety disorders, panic attacks, and in severe cases, postpartum psychosis—a psychiatric emergency characterized by delusions, hallucinations, and disorganized thinking.

Physical health suffers as well. Mothers with PPD often neglect self-care, skip medical appointments, have poor nutrition, and experience sleep disturbances beyond normal newborn-related sleep deprivation. These factors can impair recovery from childbirth and exacerbate underlying health conditions.

**Impact on Infant Development**

PPD significantly affects mother-infant bonding, which is critical for healthy child development. Mothers may struggle to respond to their baby's cues, have difficulty breastfeeding, or feel emotionally detached. Research shows that children of mothers with untreated PPD face increased risks of developmental delays, behavioral problems, emotional difficulties, and cognitive impairments. The lack of responsive caregiving during this crucial developmental window can have lasting effects on attachment patterns and emotional regulation.

**Family and Relationship Consequences**

PPD strains romantic relationships, often leading to increased conflict, decreased intimacy, and sometimes separation or divorce. Partners may experience depression themselves. Siblings may receive less attention and support, potentially developing behavioral or emotional issues.

**Long-term Complications**

Without treatment, PPD can persist for months or years, increasing vulnerability to future depressive episodes and chronic mental health problems. The cumulative effect on family functioning can be profound and intergenerational.

Fortunately, PPD is treatable through therapy, medication, and support systems, making early recognition and intervention essential.

**4.When does postpartum depression usually start? How long does postpartum depression last?**

Postpartum depression (PPD) typically begins within the first few weeks after childbirth, though its onset can vary considerably. Most commonly, symptoms emerge within the first 2-4 weeks postpartum, but PPD can develop anytime during the first year following delivery. Some women experience symptoms as early as during pregnancy (which may be classified as perinatal depression), while others don't notice significant symptoms until several months after giving birth.

It's important to distinguish postpartum depression from the "baby blues," which affects up to 80% of new mothers. The baby blues typically start within 2-3 days after delivery and resolve on their own within about two weeks. These involve mild mood swings, crying spells, anxiety, and difficulty sleeping. Postpartum depression, however, is more severe, persistent, and interferes with daily functioning.

**Duration of Postpartum Depression**

The duration of PPD varies significantly from person to person. Without treatment, postpartum depression can last for many months or even years. Some studies suggest that untreated PPD may persist for 6 months or longer in many cases, and some women continue experiencing symptoms well into their child's first year or beyond.

However, with appropriate treatment, the outlook is much more positive. Treatment typically includes therapy (particularly cognitive-behavioral therapy or interpersonal therapy), medication (such as antidepressants that are generally safe during breastfeeding), support groups, or a combination of approaches. With proper care, many women begin feeling better within a few weeks to months of starting treatment.

Early intervention is crucial for shortening the duration and reducing the severity of symptoms. If you or someone you know is experiencing persistent sadness, anxiety, difficulty bonding with the baby, changes in appetite or sleep beyond normal newborn disruptions, or thoughts of harming oneself or the baby, it's essential to reach out to a healthcare provider immediately. Postpartum depression is treatable, and seeking help is a sign of strength, not weakness.

**5.What are the risk factors for postpartum depression?**

Postpartum depression (PPD) is a complex condition influenced by multiple interconnected risk factors spanning biological, psychological, and social domains.

**Biological and hormonal factors** play a significant role. The dramatic drop in estrogen and progesterone following childbirth can affect neurotransmitter systems regulating mood. Women with a personal history of depression or anxiety, or a family history of mood disorders, face elevated risk. Previous episodes of postpartum depression increase the likelihood of recurrence in subsequent pregnancies. Thyroid dysfunction, which can occur postpartum, may also contribute to depressive symptoms.

**Psychosocial stressors** represent another major category of risk. Lack of social support—whether from a partner, family, or friends—is strongly associated with PPD. Relationship difficulties or domestic violence create additional vulnerability. Financial strain, housing instability, and employment concerns can compound stress during an already challenging transition. Unplanned or unwanted pregnancies may increase risk, as can ambivalent feelings about motherhood.

**Pregnancy and birth-related factors** also matter. Complications during pregnancy or delivery, premature birth, or having a baby requiring intensive care can be traumatic. Difficulties with breastfeeding often contribute to feelings of inadequacy and distress. Sleep deprivation, nearly universal among new parents, significantly impacts mental health and can exacerbate depressive symptoms.

**Individual characteristics** including young maternal age, single parenthood, and a history of childhood trauma or abuse increase susceptibility. Perfectionist tendencies or unrealistic expectations about motherhood can set women up for disappointment and self-criticism.

Understanding these risk factors is crucial because postpartum depression is treatable, and early identification allows for timely intervention. Women experiencing multiple risk factors deserve particular attention and support from healthcare providers, and anyone experiencing symptoms should seek help without shame or delay.

**6.How do you tell postpartum blues from postpartum depression?**

The distinction between postpartum blues and postpartum depression is primarily about severity, duration, and impact on functioning.

**Postpartum Blues ("Baby Blues")**

This is extremely common, affecting 50-80% of new mothers. It typically begins within the first few days after delivery, peaks around day 5, and resolves on its own within two weeks. The symptoms are relatively mild and include mood swings, tearfulness, anxiety, irritability, feeling overwhelmed, and difficulty sleeping even when the baby sleeps. Despite these feelings, mothers with baby blues can still function, bond with their baby, and experience moments of joy. No treatment is usually needed beyond rest, support, and reassurance.

**Postpartum Depression**

This is a more serious condition affecting about 10-20% of new mothers. It can begin anytime within the first year after delivery, though it often starts within the first few weeks to months. The symptoms are more intense and persistent, lasting weeks or months without treatment. These include:

- Persistent sadness, hopelessness, or emptiness

- Loss of interest or pleasure in activities, including caring for the baby

- Severe anxiety or panic attacks

- Difficulty bonding with the baby or intrusive scary thoughts about the baby

- Feelings of worthlessness or excessive guilt

- Significant changes in appetite and sleep (beyond normal newborn disruption)

- Difficulty concentrating or making decisions

- Thoughts of harming oneself or the baby

The key differences are **duration** (blues resolve within 2 weeks; depression persists), **severity** (depression significantly impairs daily functioning), and **ability to cope** (blues are manageable; depression feels overwhelming and unmanageable).

If symptoms persist beyond two weeks, worsen over time, or interfere with caring for yourself or your baby, it's important to contact a healthcare provider. Postpartum depression is treatable with therapy, support groups, and sometimes medication, and seeking help is crucial for both mother and baby's wellbeing.

**7.Does a prior history of depression or anxiety increase PPD risk?**

Yes, a prior history of depression or anxiety significantly increases the risk of postpartum depression (PPD). This is one of the most consistently documented risk factors in the research literature.

**Depression History**

Women who have experienced depression before pregnancy face a substantially elevated risk of developing PPD. Those with a history of major depressive disorder are approximately 2-3 times more likely to experience PPD compared to women without such history. The risk is even higher for women who have previously experienced postpartum depression—studies suggest that up to 40-50% of women who had PPD after one pregnancy will experience it again with subsequent pregnancies.

**Anxiety Disorders**

Pre-existing anxiety disorders, including generalized anxiety disorder, panic disorder, obsessive-compulsive disorder, and social anxiety disorder, also significantly increase PPD vulnerability. Anxiety and depression frequently co-occur, and prenatal anxiety is a particularly strong predictor of postpartum mood disturbances. Women with anxiety disorders may find the uncertainties and demands of new motherhood especially challenging, potentially triggering or exacerbating symptoms.

**Why This Connection Exists**

Several factors explain this increased risk. Biological vulnerability plays a role—individuals with mood disorder histories may have neurochemical sensitivities that make them more susceptible to the hormonal fluctuations following childbirth. Additionally, the stress of pregnancy, childbirth, and caring for a newborn can act as triggers in those already predisposed to mood disorders.

**Clinical Implications**

Healthcare providers should carefully screen pregnant women for mental health history during prenatal care. Women with prior depression or anxiety should receive closer monitoring during pregnancy and postpartum, potentially including preventive interventions such as therapy, increased social support, or in some cases, medication. Early identification and proactive management can significantly reduce the likelihood and severity of PPD in high-risk women.

If you have a history of depression or anxiety and are pregnant or planning pregnancy, discussing this openly with your healthcare provider can help ensure appropriate support and monitoring.

**8.What are early warning signs of postpartum depression?**

Postpartum depression (PPD) affects approximately 1 in 7 new mothers and can develop anytime within the first year after childbirth, though it most commonly emerges within the first few weeks to months. Recognizing early warning signs is crucial for getting timely support and treatment.

**Emotional and Mood Changes**

One of the most prominent signs is persistent sadness, emptiness, or hopelessness that doesn't lift after a few days. Unlike the "baby blues" that typically resolve within two weeks, PPD involves mood changes that intensify or persist. You might experience severe mood swings, crying spells that seem excessive or occur without clear reason, or feelings of worthlessness and inadequacy about your ability to care for your baby.

Irritability and anger are also common warning signs that are sometimes overlooked. You may find yourself feeling unusually short-tempered with your partner, older children, or even the baby, or experiencing anger that feels disproportionate to the situation.

**Changes in Connection and Interest**

Difficulty bonding with your baby or feeling emotionally disconnected is a significant warning sign. This might manifest as a lack of interest in the baby, difficulty feeling love or attachment, or intrusive thoughts about the baby being harmed. You might also withdraw from your partner, family, and friends, or lose interest in activities you previously enjoyed.

**Physical and Cognitive Symptoms**

PPD often includes physical manifestations such as significant changes in appetite (eating much more or less than usual), sleep disturbances beyond normal newborn care disruptions (inability to sleep even when the baby sleeps, or sleeping excessively), fatigue and loss of energy that feels overwhelming, and physical aches without clear cause.

Cognitive symptoms include difficulty concentrating, making decisions, or remembering things. You might experience racing thoughts or find your mind going blank. Severe anxiety, panic attacks, or obsessive thoughts can also occur.

**Concerning Thoughts**

Thoughts of harming yourself or the baby require immediate attention. This also includes feeling that your family would be better off without you or having recurrent thoughts about death.

If you're experiencing several of these symptoms for more than two weeks, or if any symptom is severe, reaching out to your healthcare provider is important. PPD is treatable, and seeking help is a sign of strength, not weakness.

**9.How can partners/family recognize signs of postpartum depression?**

Postpartum depression (PPD) affects approximately 1 in 7 new mothers, and early recognition by partners and family members can be crucial for getting timely support. Here are key signs to watch for:

**Emotional and Behavioral Changes**

Partners and family should be alert to persistent sadness, crying spells that seem excessive or occur without clear triggers, and a notable loss of interest in activities the new mother previously enjoyed. Unlike the "baby blues" which typically resolve within two weeks, PPD symptoms persist and often worsen over time.

Watch for expressions of hopelessness, worthlessness, or excessive guilt—particularly around feelings of being a "bad mother." The person may seem emotionally numb or detached, unable to feel joy even during positive moments with the baby.

**Interaction with the Baby**

One concerning sign is difficulty bonding with the baby or expressing little interest in the newborn. This might manifest as avoiding eye contact with the baby, seeming disengaged during feeding or care, or expressing thoughts that she doesn't love or feel connected to her child. Conversely, some mothers develop excessive anxiety about the baby's wellbeing, checking on them constantly or expressing unfounded fears.

**Physical and Cognitive Symptoms**

Look for significant changes in sleep patterns beyond normal new-parent exhaustion—either sleeping too much when the baby sleeps or experiencing insomnia even when given the opportunity to rest. Appetite changes, whether eating much more or much less than usual, are also common.

Family members may notice the new mother has difficulty concentrating, making decisions, or remembering things. She may seem unusually irritable, agitated, or conversely, move and speak more slowly than normal.

**Social Withdrawal and Daily Functioning**

A mother experiencing PPD may withdraw from friends, family, and her partner. She might stop responding to messages, cancel plans repeatedly, or seem disinterested in social connection. There may be neglect of personal hygiene or appearance, or difficulty completing routine household tasks that were previously manageable.

**Concerning Thoughts**

Any mention of thoughts about harming herself or the baby requires immediate attention. This includes statements about the baby being "better off without me," feeling trapped, or expressing wishes to escape or disappear.

**What Partners and Family Can Do**

If you notice several of these signs persisting for more than two weeks, approach the conversation with compassion and without judgment. Acknowledge that what she's experiencing is a real medical condition, not a personal failing. Encourage her to speak with her healthcare provider, and offer to help make or attend appointments. Provide practical support with childcare, meals, and household tasks to reduce her burden.

Remember that postpartum depression is treatable, and with proper support—which may include therapy, medication, or both—most mothers fully recover.

**10.Can fathers/partners experience postpartum depression too?**

Yes, fathers and partners absolutely can experience postpartum depression. This condition is often called paternal postpartum depression (PPND) or peripartum depression when referring to non-birthing partners.

**How Common Is It?**

Research indicates that approximately 8-10% of fathers experience postpartum depression, with rates potentially higher during the 3-6 month period after birth. Partners of mothers who have postpartum depression face even higher risks, with rates sometimes reaching 25-50%.

**Why Does It Happen?**

The causes are multifaceted and differ somewhat from maternal postpartum depression. Contributing factors include:

- Significant sleep deprivation and exhaustion

- Major lifestyle changes and loss of previous routines

- Relationship strain and decreased intimacy with their partner

- Financial pressures and new responsibilities

- Feeling unprepared or inadequate as a parent

- Reduced social connections and support

- Hormonal changes (fathers do experience drops in testosterone and changes in cortisol and other hormones around the birth of a child)

- History of depression or anxiety

**What Does It Look Like?**

Symptoms in fathers and partners can manifest differently than in mothers. They may include:

- Increased irritability, anger, or hostility

- Withdrawal from family and relationships

- Working excessively or avoiding home

- Increased risk-taking behaviors

- Substance use

- Difficulty bonding with the baby

- Feelings of being overwhelmed or trapped

- Physical symptoms like headaches or digestive issues

**Why Recognition Matters**

Paternal postpartum depression often goes unrecognized because there's less awareness, fewer screening protocols for non-birthing parents, and societal expectations that men should be stoic or "tough it out." However, untreated PPND can significantly impact the entire family system, affecting partner relationships, child development, and long-term family wellbeing.

If you or someone you know is struggling, reaching out to a healthcare provider, therapist, or support group specializing in perinatal mental health is an important first step. Treatment is effective and seeking help is a sign of strength, not weakness.

**11.Can postpartum depression affect lactation or breastfeeding success?**

Yes, postpartum depression (PPD) can significantly affect both lactation and breastfeeding success, creating a complex bidirectional relationship between mental health and feeding outcomes.

**Impact on Milk Production*

Postpartum depression can interfere with the hormonal processes necessary for lactation. The stress hormone cortisol, which is often elevated in depression, can inhibit prolactin and oxytocin—the key hormones responsible for milk production and let-down reflex. Women with PPD may experience delayed lactogenesis (the onset of copious milk production), reduced milk supply, or difficulty with milk ejection. The persistent stress and anxiety associated with depression can create a cycle where worrying about milk supply actually contributes to decreased production.

**Behavioral and Practical Challenges**

Depression affects motivation, energy levels, and the ability to engage in demanding tasks. Breastfeeding, especially in the early weeks, requires frequent feeding sessions, often around the clock. Women with PPD may struggle with:

- Reduced motivation to initiate or continue breastfeeding

- Difficulty responding to infant feeding cues due to emotional numbing

- Decreased confidence in their ability to nourish their baby

- Physical exhaustion that makes frequent feeding feel overwhelming

- Impaired mother-infant bonding, which can affect the breastfeeding relationship

**The Bidirectional Relationship**

Interestingly, the relationship works both ways. Breastfeeding difficulties can contribute to or worsen postpartum depression. Women who experience pain, low milk supply, or feel they're "failing" at breastfeeding may develop or intensify depressive symptoms. Conversely, some research suggests successful breastfeeding may have protective effects against PPD due to the calming effects of oxytocin.

**Support and Treatment Considerations**

Healthcare providers need to screen for PPD and address both mental health and feeding concerns simultaneously. Treatment options include:

- Therapy (particularly cognitive-behavioral therapy)

- Antidepressant medications (many are compatible with breastfeeding)

- Lactation consultant support to address practical feeding challenges

- Partner and family support to reduce caregiving burden

It's crucial that women understand that prioritizing their mental health—whether that means continuing to breastfeed with support, combination feeding, or using formula—is essential for both maternal and infant wellbeing. The goal should be supporting the mother's recovery while respecting her feeding choices.

**12.Can postpartum depression affect a baby’s development?**

Yes, postpartum depression (PPD) can significantly affect a baby's development, though it's important to know that with proper support and treatment, these effects can often be minimized or reversed.

**How PPD Affects Baby Development**

When a mother experiences postpartum depression, it can impact her ability to engage with her baby in the responsive, attuned way that infants need for optimal development. Babies thrive on consistent emotional connection, eye contact, vocal interaction, and physical touch. Depression can make these interactions more difficult, as mothers may feel emotionally numb, exhausted, or overwhelmed.

**Specific Developmental Areas Affected**

*Emotional and social development* can be impacted when babies don't receive consistent emotional responsiveness. Infants learn to regulate their own emotions partly through their caregiver's reactions, so reduced maternal responsiveness may affect their ability to self-soothe and understand emotional cues.

*Cognitive development* may also be affected. The back-and-forth interactions between mother and baby—talking, playing, responding to the baby's sounds—are crucial for language development and cognitive growth. Depression can reduce the frequency and quality of these interactions.

*Attachment* between mother and baby can be disrupted. Secure attachment forms when caregivers consistently respond to a baby's needs, and this foundation is essential for healthy relationships throughout life.

**The Importance of Context**

The severity and duration of PPD matter significantly. Brief or mild depression may have minimal lasting impact, especially if the baby has other supportive caregivers. Severe, untreated depression that persists for months poses greater risks.

**Hope and Recovery**

It's crucial to emphasize that these effects are not inevitable or irreversible. When mothers receive appropriate treatment—whether therapy, medication, support groups, or combination approaches—they can fully recover their ability to bond with and nurture their babies. Partners, family members, and other caregivers can also provide crucial support during this time. Early intervention is key, so mothers experiencing PPD symptoms should seek help from healthcare providers without shame or delay.

**13.What treatments are available for postpartum depression (therapy/meds/combined)?**

Postpartum depression (PPD) is a serious but treatable condition, and there are several evidence-based approaches available. The most effective treatment often depends on the severity of symptoms and individual circumstances.

**Psychotherapy**

Therapy is highly effective for PPD and is often recommended as a first-line treatment, especially for mild to moderate cases. Cognitive Behavioral Therapy (CBT) helps identify and change negative thought patterns and behaviors that contribute to depression. Interpersonal Therapy (IPT) focuses on relationship issues and life transitions related to new parenthood. These approaches can be conducted individually or in group settings, and many new mothers find validation and support in connecting with others experiencing similar challenges.

**Medication**

Antidepressants, particularly selective serotonin reuptake inhibitors (SSRIs) like sertraline and paroxetine, are commonly prescribed for moderate to severe PPD. These medications are generally considered safe during breastfeeding, though it's important to discuss the risks and benefits with a healthcare provider. In 2019, the FDA approved brexanolone (Zulresso), the first medication specifically designed for PPD, which is administered intravenously over 60 hours in a medical facility. More recently, zuranolone (Zurzuvae), an oral medication for PPD, was approved in 2023, offering a more accessible option.

**Combined Approaches**

Research consistently shows that combining therapy and medication is often more effective than either treatment alone, particularly for moderate to severe PPD. This integrated approach addresses both the biological and psychological aspects of depression.

**Additional Supports**

Lifestyle interventions can complement formal treatment, including regular exercise, adequate sleep (when possible), social support, and peer support groups. Some women also find benefit from omega-3 supplements, though evidence is still emerging.

It's crucial to work with healthcare providers to develop an individualized treatment plan, as PPD is highly treatable and recovery is absolutely possible.

**14.Will I get PPD with a future pregnancy, and how can I prevent it?**

I understand you're concerned about postpartum depression (PPD) in a future pregnancy. This is a really important question to think about proactively.

**Your Risk Level**

Whether you'll experience PPD depends on several factors. If you've had PPD before, you do have an increased risk of recurrence—studies suggest around 30-50% chance in subsequent pregnancies. However, this also means many people who've experienced it once don't get it again. Other risk factors include a personal or family history of depression or anxiety, lack of social support, high stress levels, sleep deprivation, complications during pregnancy or birth, and hormonal sensitivity.

**Prevention Strategies**

While there's no guaranteed way to prevent PPD, there are meaningful steps you can take:

**Before pregnancy:** Talk with your healthcare provider about your history and concerns. They may recommend starting therapy preventatively or discuss medication options if you have a history of depression. Building a strong support network before you conceive can also be invaluable.

**During pregnancy:** Continue or start therapy, particularly cognitive-behavioral therapy (CBT) or interpersonal therapy, which have shown effectiveness. Maintain regular prenatal care and communicate openly about your mental health. Some studies suggest omega-3 supplementation might help, though you should discuss this with your doctor.

**After birth:** Have a postpartum plan in place that includes regular mental health check-ins, either with your provider or a therapist. Prioritize sleep as much as possible—this might mean accepting help with nighttime feedings. Don't isolate yourself; maintain connections with supportive people. If you're breastfeeding and need medication, know that many antidepressants are compatible with nursing.

**Early Intervention**

Perhaps most importantly, recognize that early treatment makes a significant difference. If you notice symptoms like persistent sadness, anxiety, difficulty bonding with your baby, or intrusive thoughts, reach out immediately rather than waiting to see if things improve.

Your awareness and proactive approach are already protective factors. Having honest conversations with your healthcare team throughout the process will give you the best chance at a healthy postpartum period.

**15.Do exercise, sunlight, diet, and social support help with PPD?**

Yes, exercise, sunlight, diet, and social support can all be helpful components in managing postpartum depression (PPD), though they typically work best as complementary approaches alongside professional treatment rather than replacements for it.

**Exercise** has shown promise in reducing PPD symptoms. Physical activity releases endorphins and can help regulate mood, improve sleep quality, and boost energy levels—all of which tend to be disrupted during postpartum depression. Even gentle activities like walking with the baby in a stroller or postpartum yoga can make a difference. However, new mothers should start slowly and consult with their healthcare provider before beginning any exercise routine.

**Sunlight exposure** can be particularly valuable, as it helps regulate circadian rhythms and supports vitamin D production, both of which influence mood. Many women with PPD experience disrupted sleep-wake cycles, and getting natural light exposure during the day can help recalibrate these patterns. Some research suggests that seasonal timing of birth may influence PPD risk, potentially related to sunlight availability.

**Diet** plays an important role in overall mental health. Nutritious eating supports brain function and energy levels, which are crucial when managing depression while caring for a newborn. Omega-3 fatty acids (found in fish, walnuts, and flaxseeds) have been studied for their potential mood-stabilizing effects. However, preparing healthy meals can be challenging for exhausted new parents, so practical support with meal preparation can be valuable.

**Social support** is particularly crucial for PPD recovery. Isolation often worsens depression, while connection with understanding friends, family, or other new mothers can provide emotional validation, practical help, and reduced feelings of loneliness. Support groups specifically for postpartum mothers can be especially beneficial.

That said, PPD is a serious medical condition, and these lifestyle factors should complement—not replace—professional treatment. If you or someone you know is experiencing PPD symptoms, speaking with a healthcare provider about therapy, medication, or other treatments is essential.

**16.What can family do most effectively to prevent and treat postpartum depression?**

Postpartum depression (PPD) affects about 1 in 7 new mothers and can significantly impact both the mother and family. Family members play a crucial role in both prevention and treatment.

**Prevention Strategies**

The most effective preventive measure families can provide is **practical and emotional support**. This means taking on household responsibilities—cooking, cleaning, laundry, and caring for older children—so the new mother can focus on recovery and bonding with the baby. Sleep deprivation is a major risk factor for PPD, so families should arrange shifts to allow the mother uninterrupted sleep periods of at least 3-4 hours.

Emotional support is equally vital. Family members should create a non-judgmental environment where the mother feels safe expressing doubts, fears, or negative feelings without shame. Simply listening without offering unsolicited advice can be powerful. Regular check-ins asking "How are *you* doing?" rather than only focusing on the baby helps mothers feel seen as individuals.

Families should also encourage and facilitate social connection, as isolation increases PPD risk. This might mean inviting supportive friends over, accompanying the mother to new parent groups, or arranging virtual connections when in-person visits aren't possible.

**Treatment Support**

When PPD develops, **early recognition is critical**. Family members often notice symptoms before the mother does—persistent sadness, excessive anxiety, difficulty bonding with the baby, or loss of interest in previously enjoyed activities. Gently expressing concern and encouraging professional help is essential.

Families can actively support treatment by helping schedule and attend therapy appointments, ensuring the mother takes prescribed medications consistently, and monitoring for worsening symptoms. They should also watch for warning signs that require immediate intervention, such as thoughts of self-harm or harming the baby

**What Not to Do**

Avoid minimizing her feelings with phrases like "everyone feels this way" or "just be grateful for your baby." Don't take her symptoms personally or make her feel guilty. Avoid overwhelming her with visitors or unrealistic expectations about "bouncing back."

Recovery takes time, often several months with proper treatment. Patience, consistent support, and validation throughout the journey make family involvement truly effective in combating postpartum depression.

**17.When should a patient with postpartum depression seek professional help?**

A patient with postpartum depression should seek professional help as soon as they recognize persistent symptoms that interfere with daily functioning or their ability to care for themselves or their baby. While some mood changes after childbirth are normal ("baby blues"), professional intervention becomes necessary when symptoms are more severe or last beyond two weeks postpartum.

**Immediate professional help should be sought if:**

- There are thoughts of harming oneself or the baby

- Intrusive, frightening thoughts about the baby's safety occur repeatedly

- The person feels unable to care for the baby or themselves

- There's a complete loss of interest in the baby or overwhelming fear of being alone with the baby

- Hallucinations or delusions are present (which may indicate postpartum psychosis, a medical emergency)

**Professional evaluation is also warranted when experiencing:**

- Persistent sadness, hopelessness, or crying that lasts most of the day

- Severe anxiety, panic attacks, or constant worry

- Difficulty bonding with the baby or feelings of detachment

- Significant changes in sleep (beyond normal newborn sleep disruption) or appetite

- Exhaustion that isn't relieved by rest

- Feelings of worthlessness, guilt, or inadequacy as a parent

- Inability to experience pleasure or joy

- Difficulty concentrating or making decisions

- Withdrawal from family and friends

- Physical symptoms like headaches or body aches without clear cause

It's important to understand that postpartum depression is a medical condition, not a character flaw or sign of weakness. Early intervention leads to better outcomes for both parent and baby. Healthcare providers—including obstetricians, primary care doctors, psychiatrists, or therapists—can offer various treatments including therapy, medication, support groups, or combination approaches.

Partners and family members should also encourage seeking help if they notice concerning symptoms. Delaying treatment can worsen the condition and impact the parent-child relationship and family wellbeing.

**18.Can postpartum depression go away without treatment?**

Postpartum depression (PPD) can sometimes resolve on its own, but this isn't something anyone should count on or wait for. The condition affects each person differently, and while some cases may improve naturally over time, others can persist or worsen without intervention.

Some milder cases of PPD do gradually lift within a few months, particularly when the person has strong social support, adequate rest, and the baby begins sleeping more regularly. The hormonal fluctuations that contribute to PPD also stabilize over time, which can help. However, there's no reliable way to predict whose symptoms will improve spontaneously and whose will linger.

The significant concern with waiting for PPD to resolve on its own is the potential cost of that approach. Untreated postpartum depression can:

**Impact the parent's wellbeing**: The condition can persist for months or even years, affecting quality of life, relationships, and the ability to function. In severe cases, it can lead to suicidal thoughts or actions.

**Affect the baby**: PPD can interfere with parent-infant bonding and may impact the child's emotional and developmental outcomes. Infants benefit from engaged, emotionally available caregivers.

**Strain relationships**: Depression often creates distance between partners and can add stress to an already demanding time.

**Worsen over time**: Without treatment, symptoms can become more severe or develop into chronic depression.

Treatment for PPD is highly effective and comes in various forms—therapy, support groups, medication, lifestyle changes, or combinations of these. Many people see improvement within weeks of starting treatment. Given that effective help is available and the risks of leaving PPD untreated are real, seeking professional support is strongly recommended rather than hoping symptoms will fade on their own.

If you or someone you know is experiencing symptoms of postpartum depression, reaching out to a healthcare provider is an important step toward feeling better.

**19.Can postpartum depression relapse after stopping meds, and what’s the recurrence risk?**

Postpartum depression (PPD) can indeed relapse after discontinuing medication, and understanding both relapse and recurrence risks is important for long-term treatment planning.

**Relapse After Stopping Medication**

When women discontinue antidepressants after recovering from PPD, there is a significant risk of relapse. Studies suggest that approximately 50-70% of women who stop antidepressants may experience a return of depressive symptoms, particularly if medication is discontinued abruptly or too soon after symptom resolution. The risk is highest when:

- Medication is stopped within the first 6-12 months of treatment

- Discontinuation happens rapidly rather than through gradual tapering

- There's a history of severe or treatment-resistant depression

- Other risk factors remain present (lack of support, stress, sleep deprivation)

Most clinical guidelines recommend continuing antidepressant treatment for at least 6-12 months after symptoms resolve before considering discontinuation, with careful monitoring during and after the tapering process.

**Recurrence Risk in Subsequent Pregnancies**

The recurrence risk of PPD in future pregnancies is substantial. Women who've experienced PPD have approximately a 30-50% chance of developing it again with subsequent births—significantly higher than the general population risk of 10-15%. Some studies suggest the risk may be even higher (up to 60-80%) for women with:

- Severe previous episodes

- Multiple previous episodes

- Early onset PPD (within the first few weeks postpartum)

- History of depression outside the postpartum period

**Risk Mitigation Strategies**

For women with a history of PPD planning future pregnancies, preventive approaches include:

- Prophylactic medication starting in late pregnancy or immediately postpartum

- Enhanced psychotherapy and monitoring throughout pregnancy and postpartum

- Ensuring robust social support systems

- Prioritizing sleep and self-care

- Early intervention at first symptom emergence

Close collaboration with healthcare providers is essential for developing individualized plans that balance the risks of untreated depression against medication concerns during pregnancy and breastfeeding.

**20.How do you differentiate postpartum depression from postpartum PTSD—and can they co-occur?**

Postpartum depression (PPD) and postpartum PTSD are distinct conditions, though they can absolutely co-occur and share some overlapping features.

**Postpartum Depression** typically involves persistent low mood, loss of interest in activities, feelings of worthlessness or guilt, difficulty bonding with the baby, changes in sleep and appetite, and sometimes thoughts of harming oneself or the baby. It generally develops within the first few weeks to months after delivery and represents a depressive episode triggered by childbirth.

**Postpartum PTSD**, on the other hand, stems from a traumatic birth experience or pregnancy complication. The key differentiating features include:

- **Intrusive memories** of the traumatic event—flashbacks, nightmares, or distressing thoughts about the birth

- **Avoidance behaviors**—actively avoiding reminders of the trauma, including medical settings, discussions about birth, or sometimes even the baby if they're associated with the trauma

- **Hypervigilance and hyperarousal**—being constantly on edge, easily startled, having difficulty sleeping due to feeling unsafe

- **Negative alterations in mood and cognition**—but these are specifically trauma-related, often involving feelings of detachment, blame, or persistent negative beliefs about the event

What constitutes a traumatic birth varies by individual but may include emergency cesarean sections, severe complications, feeling unheard or mistreated by medical staff, fears for the baby's or mother's life, or experiences of loss of control or dignity.

**Co-occurrence is common**—research suggests that 25-50% of women with postpartum PTSD also experience depression. The trauma can trigger depressive symptoms, while the depression can amplify the distress from traumatic memories. Additionally, both conditions can impair mother-infant bonding and require professional treatment.

Treatment approaches differ somewhat: PPD typically responds to antidepressants and supportive therapy, while PTSD often requires trauma-focused therapies like EMDR (Eye Movement Desensitization and Reprocessing) or trauma-focused CBT. When conditions co-occur, integrated treatment addressing both is essential for recovery.
